# A substrate binding model for the KEOPS tRNA modifying complex

**DOI:** 10.1038/s41467-020-19990-5

**Published:** 2020-12-04

**Authors:** Jonah Beenstock, Samara Mishelle Ona, Jennifer Porat, Stephen Orlicky, Leo C. K. Wan, Derek F. Ceccarelli, Pierre Maisonneuve, Rachel K. Szilard, Zhe Yin, Dheva Setiaputra, Daniel Y. L. Mao, Morgan Khan, Shaunak Raval, David C. Schriemer, Mark A. Bayfield, Daniel Durocher, Frank Sicheri

**Affiliations:** 1 The Lunenfeld-Tanenbaum Research Institute, Mount Sinai Hospital, Toronto, ON Canada; 2 Department of Molecular Genetics, University of Toronto, Toronto, ON Canada; 3 Department of Biology, York University, Toronto, ON Canada; 4 Department of Biochemistry, University of Toronto, Toronto, ON Canada; 5 Department of Chemistry, University of Calgary, Calgary, AB Canada; 6 Department of Biochemistry and Molecular Biology, University of Calgary, Calgary, AB Canada

**Keywords:** Chemical modification, RNA modification, tRNAs, X-ray crystallography

## Abstract

The KEOPS complex, which is conserved across archaea and eukaryotes, is composed of four core subunits; Pcc1, Kae1, Bud32 and Cgi121. KEOPS is crucial for the fitness of all organisms examined. In humans, pathogenic mutations in KEOPS genes lead to Galloway–Mowat syndrome, an autosomal-recessive disease causing childhood lethality. Kae1 catalyzes the universal and essential tRNA modification N^6^-threonylcarbamoyl adenosine, but the precise roles of all other KEOPS subunits remain an enigma. Here we show using structure-guided studies that Cgi121 recruits tRNA to KEOPS by binding to its 3’ CCA tail. A composite model of KEOPS bound to tRNA reveals that all KEOPS subunits form an extended tRNA-binding surface that we have validated in vitro and in vivo to mediate the interaction with the tRNA substrate and its modification. These findings provide a framework for understanding the inner workings of KEOPS and delineate why all KEOPS subunits are essential.

## Abstract (web summary)

KEOPS is an evolutionary conserved complex with a core of four core subunits—Pcc1, Kae1, Bud32 and Cgi121—that catalyzes the universal and essential tRNA modification N^6^-threonylcarbamoyl adenosine (t^6^A). Here the authors describe a Cgi121-tRNA crystal structure and new composite model of the KEOPS holo-enzyme-substrate complex that shed light on the t^6^A catalytic cycle and its regulation.

## Introduction

The Kae1 enzyme family is part of a small group of ~60 genes found in the genomes of all autonomous cellular life forms^1^. Reflecting their phylogenetic ubiquity and high conservation, Kae1 enzymes are essential for the fitness of practically all cells and organisms^2,3^ with the exception of the bacterium *Streptococcus mutans*^4^. In archaea and in the cytoplasm of eukaryotes, Kae1 resides within the KEOPS (kinase, endopeptidase and other proteins of small size) complex, together with the atypical protein kinase/ATPase Bud32 and the small proteins Cgi121 and Pcc1^5,6^ (for a schematic view of KEOPS architecture, see Supplementary Fig. 1a). A fifth KEOPS subunit, Gon7, is specific to eukaryotes^5,7^. In humans, pathogenic mutations have been identified in all five KEOPS genes. These mutations give rise to Galloway–Mowat syndrome (GAMOS), a severe autosomal-recessive disease that manifests in developmental defects including renal dysfunction and microcephaly, leading to early childhood mortality^8–10^.

The central biochemical function of Kae1 enzymes is to catalyze the final step of the universal tRNA modification N^6^-threonylcarbamoyl adenosine (t^6^A)^11–13^. The t^6^A modification is found specifically at position A37 of ANN-decoding tRNAs (tRNAs that pair with codons starting with an A)^14,15^. t^6^A promotes translational fidelity by facilitating the structural stability of the tRNA anticodon loop and by increasing the binding energy between tRNAs and their cognate codons in mRNA^16,17^.

Consistent with the discovery of pathogenic mutations in all KEOPS genes, genetic and biochemical studies have shown that the full composition of the KEOPS complex is required for Kae1 to carry out its essential biochemical function in archaea, yeast, fruit flies, zebrafish and mice^5,9,18–20^. Interestingly, despite being required for tRNA modification, the precise roles of Bud32, Cgi121, and Pcc1 are barely known. The ATPase activity of Bud32 is potentiated by tRNA^21,22^ and by direct binding to Cgi121^23^ and is essential for t^6^A catalytic activity^22^. Pcc1, which binds directly to Kae1, can mediate the dimerization of KEOPS^23,24^. However, this activity of Pcc1 is not required for t^6^A catalytic activity^24^ and is obstructed in eukaryotes by Gon7 binding to Pcc1^7,8,25^. Remarkably, the Kae1 orthologs in bacteria and in the mitochondria reside in protein complexes that vary considerably in size and composition from KEOPS^14^. TsaD, the bacterial ortholog, functions in a complex with TsaB and TsaE, proteins that are unrelated in structure or apparent function to any of the auxiliary subunits in KEOPS^18^. Qri7, the mitochondrial ortholog, represents the most simplified family member and functions without additional subunits^26,27^. These differences in holo-enzyme architecture highlight the mystery of the t^6^A catalytic mechanism and its regulation.

In this work, we have identified Cgi121 as a tRNA-binding subunit required for efficient substrate recruitment to KEOPS. Structural analysis of the Cgi121–tRNA complex revealed that Cgi121 binds tRNA via the 3′ CCA tail, an element universally common to mature tRNAs that serves as the accepting site for aminoacylation. Integration of the Cgi121–tRNA crystal structure into a composite model of KEOPS surprisingly implicates all four KEOPS subunits in the positioning of the A37 modification site of tRNA into the catalytic cleft of Kae1. Detailed functional characterizations validate these findings and shed light on the t^6^A catalytic cycle, its regulation, and the inner workings of an intricate evolutionarily conserved molecular machine.

## Results

### *mj*Cgi121 is a tRNA-binding protein

**Fig. 1:**
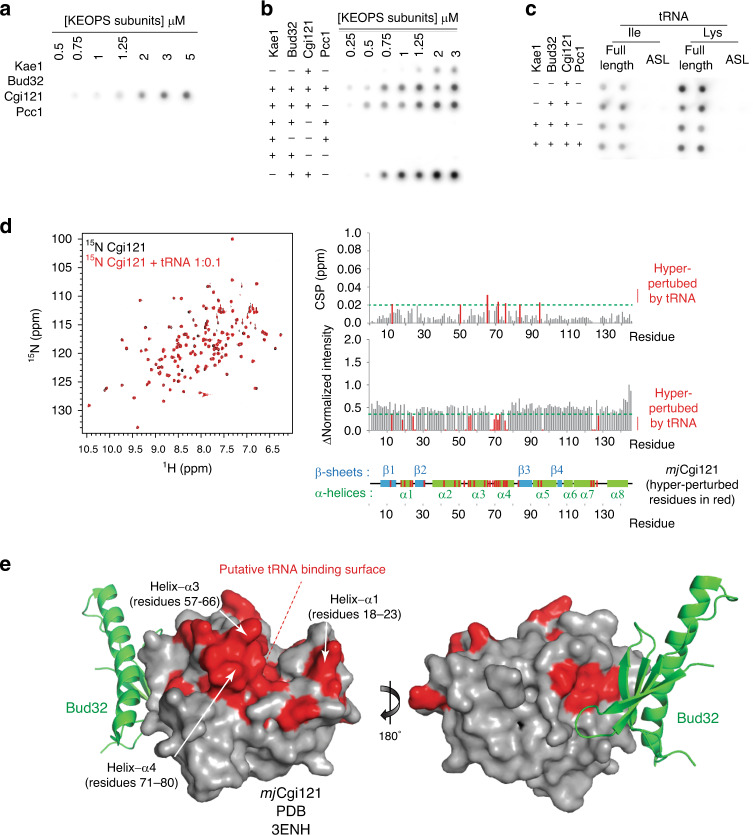
*mj*Cgi121 is the tRNA-binding subunit of *ar*KEOPS. **a**, **b** Binding activity of *ar*KEOPS, individual subunits, and subcomplexes towards *mj*tRNA^Lys^ assessed using a filter binding assay. Protein concentrations and the specific *ar*KEOPS proteins tested are indicated. **c** Binding activity of *ar*KEOPS *mj*Cgi121-containing complexes at 1 µM concentration towards full length *mj*tRNA^Lys^, *sc*tRNA^Ile^, or their respective anticodon stem loops (ASL) assessed using the filter binding assay. **d** (Left) Nuclear magnetic resonance ^1^H-^15^N-HSQC analysis of ^15^N-labeled *mj*Cgi121 in the presence or absence of 1:0.1 molar ratio of *mj*tRNA^Lys^. (Right) Chemical shift perturbations (CSPs) and the peak intensity changes of assigned resonances are shown. Residues with CSP ≥ 0.02 ppm (top graph) or peak intensity changes of ≤0.4 (bottom graph) were considered hyper-perturbed by the tRNA. Thresholds are highlighted on graphs by horizontal dashed green lines. Hyper-perturbed residues and their projections on a schematic representation of the secondary structure of *mj*Cgi121 (bottom) are highlighted in red. **e** Projection of hyper-perturbed residues from (**d**) in red on the surface structure of *mj*Cgi121 (PDB 3ENH) identifies a putative tRNA-binding surface. Bud32 (green ribbon) was shown previously to bind to a site remote from the identified tRNA-binding surface. For clarity, only *mj*Cgi121 and part of *mj*Bud32 are shown (residues 344-419 of the naturally occurring *mj*1130 Kae1-Bud32 fusion protein).

We reconstituted the archaea (*ar*) KEOPS complex with purified *Methanocaldococcus jannaschii* (*mj*) Kae1, Bud32, and Cgi121 proteins together with *Pyrococcus furiosus* (*pf*) Pcc1 (Supplementary Fig. 1a). The ability of *mj*Kae1 to modify a tRNA substrate (in this case *mj*tRNA^Lys^_UUU_) depended on the presence of the auxiliary *pf*Pcc1, *mj*Bud32, and *mj*Cgi121 subunits (Supplementary Fig. 1b). We hypothesized that one or more auxiliary subunits would function to bind tRNA. Remarkably, in contrast to previous reports^22^, using a filter binding assay we observed that only *mj*Cgi121 in isolation could detectably bind tRNA (Fig. 1a). Furthermore, subcomplexes containing *mj*Cgi121 demonstrated tRNA binding, while those lacking *mj*Cgi121 did not (Fig. 1b). Interestingly, *mj*Cgi121 in complex with *mj*Bud32 manifested the strongest binding signal (a finding probed further below). Anticodon stem loops of *mj*tRNA^Lys^ and *Saccharomyces cerevisiae* (*sc*) tRNA^Ile^, which are not sufficient for t^6^A modification (Supplementary Fig. 1c)^28,29^, were also not competent for binding to KEOPS (Fig. 1c), suggesting that the detected binding interactions are functionally relevant for tRNA modification.

### *mj*Cgi121 binding to tRNA is directed primarily at the 3′ CCA tail

Using ^15^N-*mj*Cgi121 and 1 mole equivalence of tRNA, we confirmed a direct binding interaction by NMR, as evidenced by a complete loss of resonance peaks in the HSQC spectrum (Supplementary Fig. 1d). Interestingly, sub-stoichiometric titration of tRNA with ^15^N*-mj*Cgi121 identified a cohort of residues that were especially sensitive to tRNA binding (Fig. 1d). These residues mapped to a discrete surface on *mj*Cgi121 composed primarily of helices-α1, -α3, and -α4 (Fig. 1e).

**Fig. 2:**
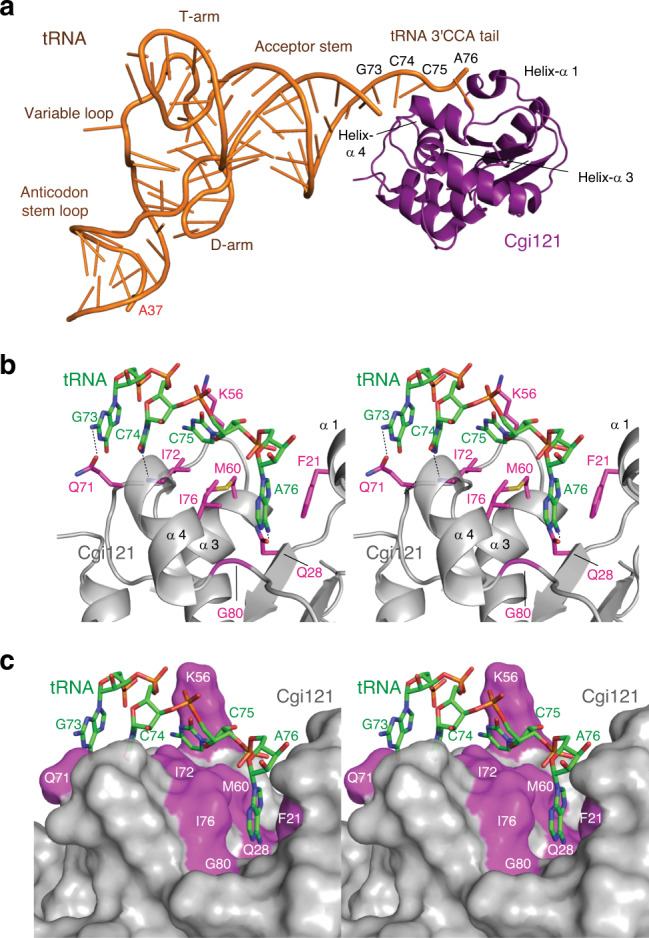
Crystal structure of the *mj*Cgi121–*mj*tRNA^Lys^_UUU_ complex. **a** Ribbon representation of the *mj*Cgi121 (purple) bound to *mj*tRNA^Lys^_UUU_ (orange). **b**, **c** Zoom-in stereo views of the binding interface between *mj*Cgi121 and *mj*tRNA^Lys^_UUU_. For ease of viewing, only the tRNA tail region encompassing 5′-^73^GCCA^76^-3′ tail (green) and side chains of Cgi121 mediating direct contacts are shown in stick representation.

To determine the precise binding mode, we solved the X-ray crystal structures of the *mj*tRNA^Lys^_UUU_–*mj*Cgi121 complex (Fig. 2a) and of *mj*tRNA^Lys^_UUU_ alone (apo) (Supplementary Fig. 2a) at 3.4 Å and 3.1 Å resolution, respectively (see also Supplementary Table 1 for data collection and refinement statistics). Overall, *mj*Cgi121 engaged the tRNA in a manner that sterically caps its free 5′ and 3′ termini. *mj*Cgi121 contacts the tRNA through an extensive surface (1162 Å^2^) involving helices-α1, -α3, and -α4, in agreement with the NMR analysis (Fig. 2a). Contacts with the tRNA are directed largely at the universal 3′ CCA tail (C74, C75, and A76) (see Supplementary Fig. 2b for an unbiased electron density map), and to a lesser degree at G73. Binding to tRNA induces minor structural changes to *mj*Cgi121 compared to the structure of *mj*Cgi121 without tRNA (RMSD = 1.6 Å over 136 residues)^23^ (Supplementary Fig. 2c).

The *mj*Cgi121–tRNA interface involves a mixture of hydrophobic, hydrogen-bonding and salt interactions (Fig. 2b, c). The base of A76 engages a deep hydrophobic pocket formed by Gly80 and the side chains of Phe21, Met60, and Ile76. N^6^ of the A76 base forms a hydrogen-bond to the side chain of Gln28. The ribose 3′ hydroxyl group of A76 is tightly packed against Cgi121, which may disfavor the binding of aminoacylated tRNAs (Supplementary Fig. 2d). The base of C75 forms hydrophobic contacts with the side chains of Lys56, Met60, and Ile72. The base of C74 forms hydrophobic contacts with the Ile72 side chain, and a hydrogen-bond with the peptide backbone of Ile72. The phosphate group of C74 is positioned to interact with the side chain of Lys56. G73 is positioned to hydrogen-bond with the side chain of Gln71. Beyond G73 few contacts of significance are made with *mj*Cgi121 (for an overview of the conservation of residues that interact with the CCA tail, see Supplementary Fig. 2e and ref. ^23^).

Apo-*mj*tRNA^Lys^_UUU_ displays a canonical L-shaped fold that is essentially unchanged in binding to *mj*Cgi121 (RMSD = 1 Å over 64 nucleotides) with two exceptions (Supplementary Fig. 2f). Firstly, the conformation of the single-stranded 3′ terminus (G73-A76), which is known to be highly flexible (see Supplementary Fig. 2g), adopts a specific conformation in response to direct binding to *mj*Cgi121. Secondly, a portion of the anticodon loop is disordered in the tRNA–Cgi121 co-structure (Supplementary Fig. 2f). This region is normally stabilized by t^6^A^17^ and its ordered nature in the apo–tRNA structure appears to be due to crystal packing interactions. In conclusion, these crystallographic analyses revealed a binding mode of *mj*Cgi121 to tRNA that is counter-intuitively directed at a universal element of tRNAs that is maximally distant (>65 Å) from the A37 site of t^6^A modification.

### CCA tail binding by *mj*Cgi121 is required for tRNA binding and t^6^A modification activity by archaeal KEOPS

**Fig. 3:**
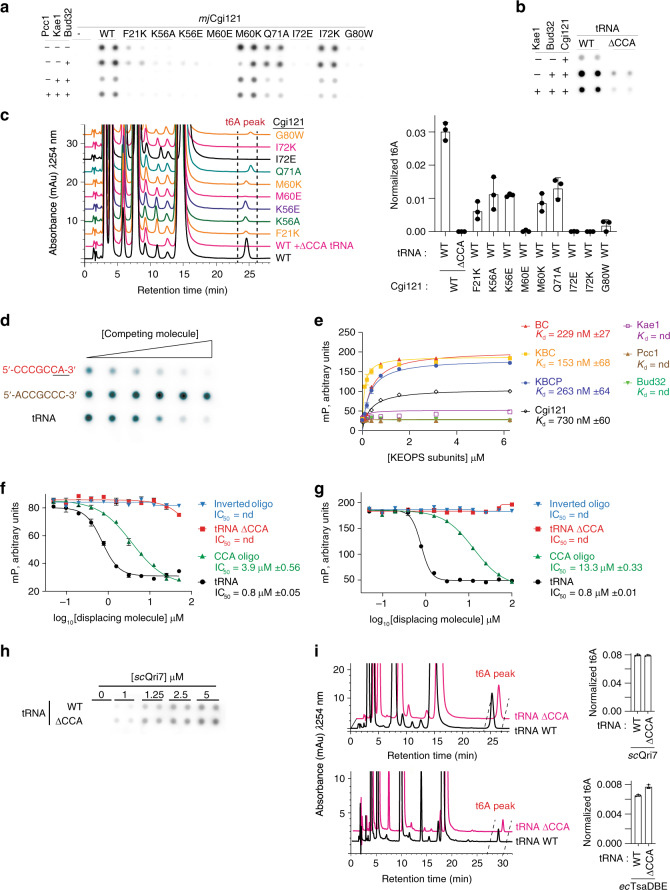
The CCA tail of tRNA is required for binding to Cgi121 and for the t^6^A tRNA modifying function of KEOPS in vitro. **a** Effect of mutations in *mj*Cgi121 on the tRNA-binding activity of *mj*Cgi121 alone and in complex with the indicated KEOPS subunits. *mj*tRNA^Lys^ was incubated with Cgi121 in isolation (10 µM) or with the indicated *ar*KEOPS subcomplexes (1 µM) using a filter binding assay. **b** Effect of deletion of the CCA tail of tRNA on binding to *mj*Cgi121 alone and in complex with the indicated KEOPS subunits. Wild-type *mj*tRNA^Lys^ and *mj*tRNA^Lys, ΔCCA^ were tested as in (**a**). **c** t^6^A modification activity analysis of *ar*KEOPS complexes reconstituted with the indicated WT and mutant *mj*Cgi121 proteins. *mj*tRNA^Lys^ or *mj*tRNA^Lys, ΔCCA^ were used as substrates. Representative HPLC profiles of nucleoside composition for each reaction are shown at left. Quantification of average t^6^A content normalized to the content of uridine is shown at right (*n* = 3 independent experiment samples, ±SD). **d** An oligonucleotide encompassing the CCA tail of tRNA (5′-CCCGCCA-3′), but not an oligo with inverted sequence (5′-ACCGCCC-3′), can competitively displace wild-type radiolabeled tRNA from binding to *mj*Cgi121 (5 µM) in a filter binding assay. Unlabeled oligonucleotides or *mj*tRNA^Lys^ (positive control) were added at final concentrations of 31.25, 62.5, 125, 250, 500, or 1000 nM to reactions with 50 nM ^32^P-labeled tRNA. **e** Binding of the indicated *ar*KEOPS components to the 647-CCA probe measured by fluorescence polarization (BC = *mj*Cgi121–*mj*Bud32; KBC = *mj*Cgi121–*mj*Bud32–*mj*Kae1; KBCP = *mj*Cgi121–*mj*Bud32–*mj*Kae1–*pf*Pcc1). Representative curves and the average *K*_d_ are shown (*n* = 3 independent experiment samples, ±SD). nd, not determined. **f**, **g** Competitive displacement of a 647-CCA binding probe to *mj*Cgi121 (**f**) or *mj*Cgi121–*mj*Bud32 (**g**) by increasing concentration of unlabeled *mj*tRNA^Lys^, *mj*tRNA^Lys, ΔCCA^, 5′-CCCGCCA-3′ or 5′-ACCGCCC-3′ oligonucleotides as measured by fluorescence polarization (*n* = 3 independent experiment samples, ±SD). **h** tRNA-binding activity of *sc*Qri7 to *mj*tRNA^Lys^ and *mj*tRNA^Lys, ΔCCA^ assessed using the filter binding assay. **i** In vitro t^6^A modification activity of *sc*Qri7 (upper) and the *ec*TsaD–TsaB–TsaE complex (lower) towards *mj*tRNA^Lys^ and *mj*tRNA^Lys, ΔCCA^ substrates. Representative HPLC profiles of nucleoside composition for each reaction (left) and quantification (right) of average t^6^A content normalized to the content of uridine (*n* = 3 independent experiment samples, ±SD).

We next mutated a selection of tRNA contact surface residues on *mj*Cgi121 to alter either the charge or size properties (Phe21Lys, Lys56Ala/Glu, Met60Glu/Lys, Gln71Ala, Ile72Glu/Lys, and Gly80Trp) to examine effects on tRNA binding (Fig. 3a). All mutations (with exception of *mj*Cgi121^Met60Lys^) had an inhibitory effect on tRNA binding in the filter binding assay (Fig. 3a). Although *mj*Cgi121^Gln71Ala^ and *mj*Cgi121^Ile72Lys^ maintained tRNA-binding activity on their own, they did not support binding in larger KEOPS subcomplexes (e.g. with Bud32–Kae1–Pcc1). Importantly, all *mj*Cgi121 mutants tested maintained the ability to bind *mj*Bud32 at a level comparable to that of *mj*Cgi121^WT^ (Supplementary Fig. 3a). Similarly, deletion of the tRNA CCA tail (denoted tRNA^ΔCCA^) greatly reduced binding to *mj*Cgi121 alone or in its complexes with *mj*Bud32 and *mj*Bud32–*mj*Kae1 (Fig. 3b). Furthermore, even deletion of A76 was sufficient to decrease tRNA binding by the *mj*Cgi121–*mj*Bud32 complex (Supplementary Fig. 3b). In total, these results support the notion that the crystal structure reflects the mode by which *mj*Cgi121 and larger KEOPS complexes bind tRNA in solution.

Interestingly, *mj*Cgi121^WT^ (MW = 17 kDa) purified from *Escherichia coli* eluted from a size exclusion column as two distinct peaks with estimated sizes of 20 and 40 kDa (Supplementary Fig. 3c). PAGE analysis revealed an enrichment of RNA species with sizes suggestive of tRNA (~24 kDa/70–90 bps) in the earlier-eluting peak. In contrast, *mj*Cgi121^Met60Glu^ displayed greatly reduced co-purification with RNA (Supplementary Fig. 3c). These findings indicated that similar to the full *P. abyssi* KEOPS complex^21^, *mj*Cgi121 is sufficient for binding tRNA in a heterologous cell system in a manner consistent with the determined X-ray crystal structure.

Paralleling the effect on tRNA binding, disruption of the *mj*Cgi121–CCA tail interface greatly inhibited *ar*KEOPS t^6^A activity (Fig. 3c). Most notably, *ar*KEOPS did not detectably modify tRNA^ΔCCA^. Likewise, *ar*KEOPS containing *mj*Cgi121^Met60Glu^, *mj*Cgi121^Ile72Glu^, or *mj*Cgi121^Ile72Lys^, did not detectably modify tRNA^WT^ (Fig. 3c). We conclude that the ability of *mj*Cgi121 to recognize the CCA tail is essential for the tRNA binding and t^6^A modification activity of *ar*KEOPS in vitro.

### The CCA tail is sufficient for binding to *mj*Cgi121

We next investigated if the 3′ tail of the tRNA is sufficient to bind *mj*Cgi121. In support, we observed that an oligonucleotide comprising the 3′ tail (5′-CCCGCCA-3′; denoted CCA-oligo) but not a control inverted oligo (5′-ACCGCCC-3′), was an effective competitor of tRNA binding to *mj*Cgi121 in the filter binding assay (Fig. 3d). Supporting these observations, further analysis using NMR confirmed binding of the CCA-oligo, but not the inverted oligonucleotide to ^15^N*-mj*Cgi121 (Supplementary Fig. 3d).

To enable quantitative measurement of binding affinities, we labeled a 5-CGCCA-3′oligonucleotide with Alexa-647 at the 5′ terminus (denoted 647-CCA). Using fluorescence polarization as a readout, we found that *mj*Cgi121 bound to 647-CCA (*K*_d_ = 730 nM), while *mj*Bud32, *mj*Kae1 and *pf*Pcc1 did not. Interestingly, the *mj*Cgi121–*mj*Bud32, and *mj*Cgi121–*mj*Bud32–*mj*Kae1 subcomplexes as well as the full KEOPS complex all bound 647-CCA with ~3.2–4.8 fold enhanced affinity relative to *mj*Cgi121 alone (*K*_d_ of 229 nM, 153 nM and 263 nM, respectively). Since none of the other individual KEOPS subunits interacted directly with this probe, the enhanced affinity is most likely through an allosteric effect on *mj*Cgi121 (Fig. 3e).

Confirming a shared binding mode between tRNA and the 647-CCA probe, *mj*Cgi121 mutations that inhibited tRNA binding also perturbed 647-CCA binding (Supplementary Fig. 3e). Furthermore, tRNA^WT^ competitively displaced 647-CCA from *mj*Cgi121, while tRNA^ΔCCA^ did not (Fig. 3f). Similar results were observed with the *mj*Cgi121–*mj*Bud32 complex (Fig. 3g). However, tRNA was ~17-fold more potent at displacing the 647-CCA probe than an unlabeled CCA-oligo, suggesting that features beyond the CCA tail may contribute to the binding of tRNA when Cgi21 is complexed with other KEOPS subunits.

In agreement with predictions that *mj*Cgi121 would not efficiently bind aminoacylated tRNAs due to steric considerations (Supplementary Fig. 2d), CCA-oligos that harbor ribose 3′ chemical modifications (Supplementary Table 2) displayed markedly reduced potency in displacing the 647-CCA probe (Supplementary Fig. 3g). Lastly, 647-CCA binding to *mj*Cgi121 hinted that *mj*Cgi121 may be able bind tRNAs that are not KEOPS substrates. Indeed *mj*tRNA^Val^_CAC_, displaced the 647-CCA probe from *mj*Cgi121, albeit with 7.3-fold reduced efficiency relative to *mj*tRNA^Lys^_UUU_ (Supplementary Fig. 3f, compare to Fig. 3f).

Together, these results demonstrate that the CCA tail is necessary and sufficient for tRNA binding to *mj*Cgi121 and that *mj*Bud32 enhances the binding of *mj*Cgi121 to tRNA, possibly through allostery and by reinforcing secondary interactions.

### The catalytic function of the Kae1-family enzymes Qri7 and TsaD does not depend on the CCA tail

Unlike Kae1, Qri7 functions in the absence of binding partners to modify tRNA in the mitochondria^27,30^ and TsaD functions with the KEOPS-unrelated TsaB–TsaE proteins to modify tRNA in bacteria^18^. Thus, Qri7 and TsaD function independent of a Cgi121-like subunit. We therefore hypothesized that the activities of Qri7 and TsaD will not depend on the tRNA CCA tail. In the filter binding assay *S. cerevisiae* (*sc*) Qri7 did not discriminate between tRNA^WT^ and tRNA^ΔCCA^ for binding (Fig. 3h). Likewise, *sc*Qri7 did not detectably bind the 647-CCA probe (Supplementary Fig. 3h) and furthermore displayed comparable ability to t^6^A modify tRNA^WT^ and tRNA^ΔCCA^ (Fig. 3h). Similar to *sc*Qri7, the *E. coli* (*ec*) TsaD–TsaB–TsaE complex also displayed comparable ability to t^6^A-modify tRNA^WT^ and tRNA^ΔCCA^ (Fig. 3i). These results suggest that recognition of tRNA through the CCA tail is an adaptation specific to KEOPS and is not relevant for the catalytic function of Qri7 and TsaD.

### Human KEOPS is dependent on the tRNA-binding function of Cgi121

To determine if the tRNA-binding mechanism is evolutionary conserved, we tested the functional dependency of human KEOPS (*h*sKEOPS) on the CCA tail. *h*sKEOPS consists of a core of four subunits; TPRKB, PRPK/TP53RK, OSGEP, and LAGE3 (orthologs of Cgi121, Bud32, Kae1, and Pcc1, respectively) (Supplementary Fig. 4a), as well as C14orf142/*hs*Gon7^7,8^. The latter subunit is absent from *ar*KEOPS^14^ and non-essential for *hs*KEOPS catalytic activity in vitro^7^ and thus was excluded from our analysis.

In the filter binding assay, TPRKB did not detectably bind tRNA (likely due to assay sensitivity limitations) whereas the TPRKB–PRPK complex displayed robust activity (Supplementary Fig. 4b). Importantly, the binding activity of TPRKB–PRPK and *h*sKEOPS was appreciably reduced toward tRNA^ΔCCA^ (Supplementary Fig. 4c). TPRKB bound 647-CCA with modest affinity (*K*_d_ = 28 µM), which was enhanced by PRPK (*K*_d_ = 0.45 µM, ~60 fold). This enhancement is similar to the effect of *mj*Bud32 on *mj*Cgi121 but more pronounced, highlighting both similarities between species as well as species-specific features. *h*sKEOPS also displayed enhanced binding affinity towards 647-CCA (*K*_d_ = 4.3 µM, ~6.5 fold) relative to TPRKB alone (Supplementary Fig. 4d). Further analysis revealed that tRNA^WT^ could displace 647-CCA from *h*sKEOPS while tRNA^ΔCCA^ could not (Supplementary Fig. 4e). Using the superimposed structures of *mj*Cgi121 and TPRKB^23^ as a guide, we generated mutations on the predicted tRNA-binding surface of TPRKB (Supplementary Fig. 4f; see also Supplementary Table 3 for list of residue equivalence). As expected, all TPRKB mutants tested were greatly deficient for binding 647-CCA (Supplementary Fig. 4g). Lastly, tRNA^ΔCCA^ had ~90% reduced efficiency as a substrate for t^6^A modification by *hs*KEOPS (Supplementary Fig. 4h). Together, these observations support a conserved tRNA-binding mechanism for KEOPS that is likely to be general to all eukaryotic and archaeal species.

### A composite KEOPS–tRNA model implicates Cgi121 in substrate presentation to Kae1

**Fig. 4:**
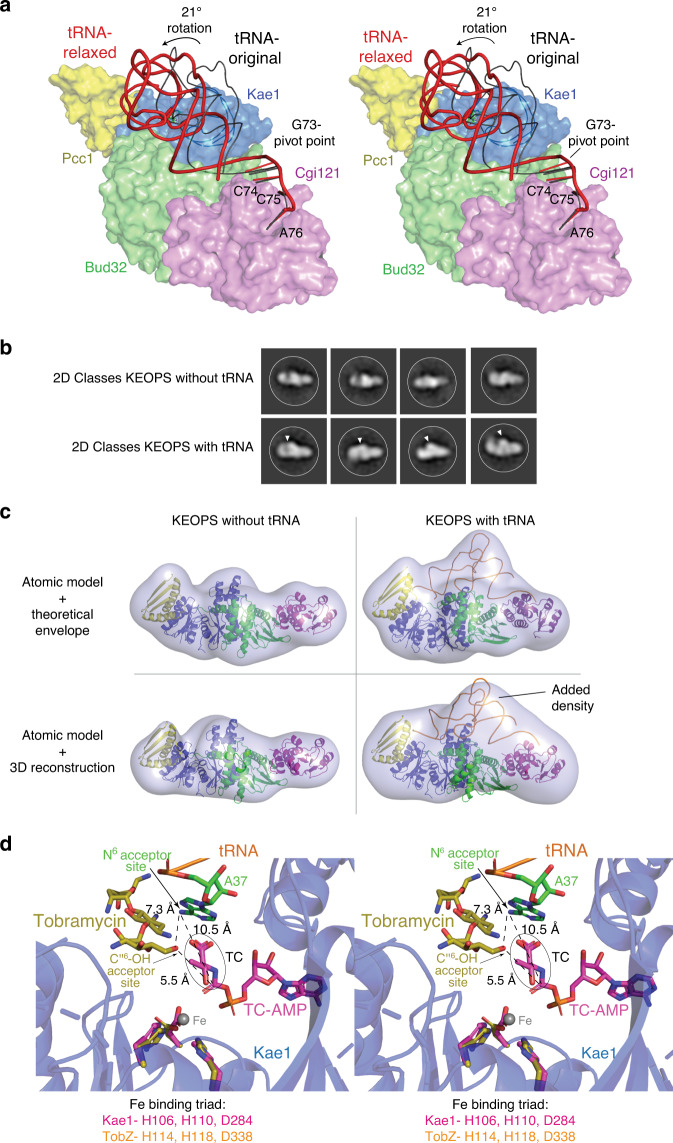
Composite model of the *ar*KEOPS holo-enzyme bound to tRNA is suggestive of an enzyme–substrate complex. **a** Stereo surface representation of *ar*KEOPS bound to *mj*tRNA^Lys^ (see methods for model construction). Black ribbon represents the position of tRNA prior to energy minimization. Note the severe steric clash between tRNA and the Kae1 subunit. Red ribbon represents the position of tRNA following energy minimization (see Methods). The steric clash between tRNA and Kae1 is relieved by a 21° rigid body pivot of tRNA centered at the junction between tRNA nucleotides G73 and C74. For clarity, only the positions of the last four bases of the tRNA are shown. **b** Representative two-dimensional class averages of KEOPS with and without tRNA determined by single particle negative stain electron microscopy. Additional density observed in the tRNA-bound specimen is indicated by the white triangles. **c** Three-dimensional reconstructions of KEOPS with and without tRNA derived from the two-dimensional-classes. (Top) atomic models and their superimposed theoretical envelopes of KEOPS alone (left) and KEOPS bound to tRNA (right). (Bottom) atomic models fitted into the 3D reconstructions of KEOPS alone (left) and KEOPS bound to tRNA (right). **d** Zoom-in stereo view of the active site region of *mj*Kae1 with the KEOPS–tRNA model (blue) highlighting the distance between the modeled position of the A37 modification acceptor site and the modification acceptor site of tobramycin as well as the predicted distances to the position of the donor atom of TC-AMP. The TobZ-tobramycin enzyme–substrate complex (PDB 3VET) was superimposed on the Kae1 subunit in the composite model shown in (**a**) by alignment of metal binding residues using Pymol.

To understand the implications of tRNA binding by Cgi121, we generated a composite model of the KEOPS holo-enzyme complex bound to tRNA. Superimposition of available structures for KEOPS subcomplexes (PDB: 3ENH and 3ENO) with the Cgi121–tRNA structure resulted in a model with clashes between the tRNA and Kae1 subdomain II (Fig. 4a). However, using in silico modeling, we generated an energetically feasible model, achieved mainly by a 21° rigid body rotation centered at G73 between the highly structured region of tRNA (nucleotides 1–72) and the rigidified CCA tail (nucleotides 74–76) tethered to *mj*Cgi121 (Fig. 4a). This rotation is within the range of flexibility of the CCA tail displayed in other tRNA structures (Supplementary Fig. 2g). Remarkably, the new model positions the tRNA in a meandering groove on KEOPS that is highly complementary to the shape of tRNA. Furthermore, all four KEOPS subunits are well positioned to contact tRNA directly and the anticodon loop that harbors the A37 modification site resides in the catalytic cleft of Kae1.

To corroborate this model, we analyzed KEOPS with and without tRNA using single-particle negative stain electron microscopy. Two-dimensional classifications from captured images revealed the expected elongated structure of KEOPS (Fig. 4b). Comparing the class averages of the two KEOPS specimens revealed clear additional density that likely corresponds to bound tRNA. Three-dimensional reconstructions revealed molecular envelopes that were consistent in size and shape with the theoretical envelopes of the corresponding atomic models, with clear added density at the expected position of tRNA (Fig. 4c). These findings support the validity of the tRNA-binding mode derived by molecular modeling.

### The KEOPS–tRNA composite model resembles an enzyme–substrate complex

To assess if our KEOPS–tRNA model is predictive of an enzyme–substrate relationship, we compared it to the structure of the TobZ–tobramycin complex. TobZ is a Kae1-like enzyme that carbamoylates the small molecule antibiotic tobramycin and thus, the co-structure of the TobZ–tobramycin complex serves as a model for the catalytic mechanism of Kae1^31^. Aligning the Kae1 and TobZ active sites by superimposition of the conserved metal binding residues showed that the N^6^ acceptor site of A37 lies 7.3 Å away from the corresponding acceptor site (hydroxyl group of C6”) of tobramycin^32^ and 10.5 Å away from the expected position of the donor atom of TC-AMP (Fig. 4d). While the distances are too large to support catalysis, they can be accommodated by local conformational changes to the anticodon loop region of tRNA, which is inherently flexible in the absence of t^6^A modification^16^ (see also Supplementary Fig. 2f). These comparisons suggest that the composite model may indeed have relevance to how *ar*KEOPS engages a tRNA substrate during the t^6^A catalytic cycle.

### Mutational analysis of the KEOPS tRNA holo-enzyme–substrate model

**Fig. 5:**
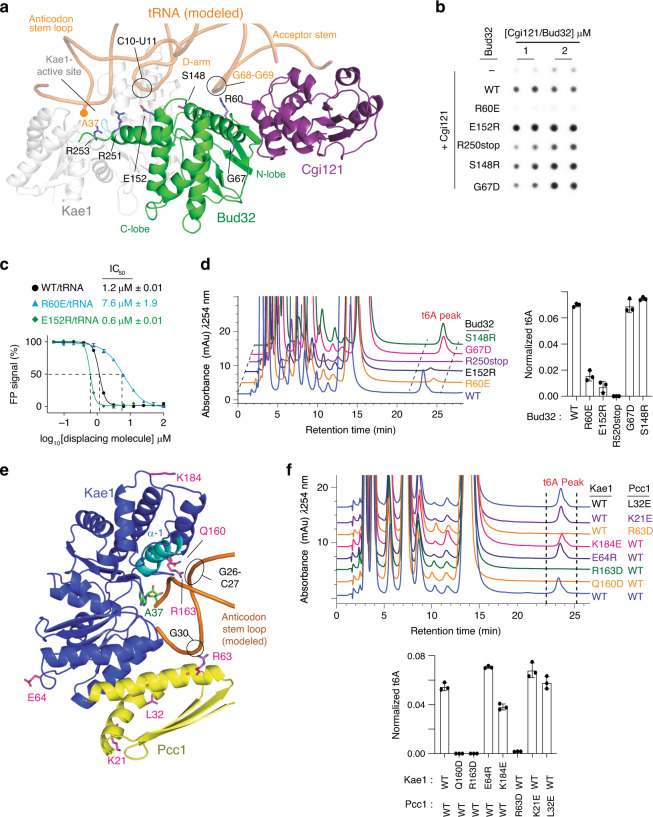
All KEOPS subunits contribute to a binding surface for tRNA substrate. **a** Ribbon representation of the *mj*Cgi121–*mj*Bud32–*mj*Kae1 complex bound to modeled tRNA. Residues in close proximity to the tRNA-binding surface of Bud32 that were selected for mutational analysis are shown in stick representation. The Pcc1 subunit was omitted for clarity. **b** Analysis of tRNA binding by *mj*Cgi121–*mj*Bud32 subcomplexes using a filter binding assay. *mj*Cgi121 was tested alone or in complex with *mj*Bud32 wild-type or the indicated mutants. **c** Competitive displacement of a 647-CCA probe from binding to the wild-type *mj*Cgi121–*mj*Bud32 complex or a complex containing the indicated Bud32 mutants by increasing concentration of *mj*tRNA^Lys^. Displacement of the 647-CCA probe was monitored by fluorescence polarization (FP) (*n* = 3 independent experiment samples, ±SD). **d** In vitro t^6^A modification activity analysis of reconstituted *ar*KEOPS complexes with wild-type *mj*Bud32 and the indicated Bud32 mutants. Representative HPLC profiles of nucleoside composition for each reaction (left) and quantification (right) showing the average t^6^A content normalized to the content of uridine (*n* = 3 independent experiment samples, ±SD). **e** Ribbon representation of the *mj*Cgi121–*mj*Bud32–*mj*Kae1–*pf*Pcc1 complex bound to modeled tRNA. Residues in close proximity to the tRNA-binding surface on Kae1 and Pcc1 that were selected for mutational analysis are shown in stick representation. *mj*Cgi121 and *mj*Bud32 were omitted for clarity. **f** In vitro t^6^A modification activity analysis of reconstituted *ar*KEOPS complexes with WT or the indicated Pcc1 and Kae1 mutants. Representative HPLC profiles of nucleoside composition for each reaction (top) and quantification (bottom) showing the average t^6^A content normalized to the content of uridine (*n* = 3 independent experiment samples, ±SD).

To explore the functional relevance of the KEOPS–tRNA model, we performed a systematic mutational analysis of conserved residues (see sequence alignments of KEOPS subunits in different species in ref. ^23^) on the predicted tRNA contact surfaces. Based on modeling, in *mj*Bud32, Arg60 in helix-αC is predicted to make attractive interactions with G68–G69 while Glu152 in the activation loop is predicted to make repulsive interactions with C10-U11 of the tRNA (Fig. 5a). In addition, Arg251 and Arg253 in the C-terminal tail of Bud32 are predicted to interact with the D-arm and the anticodon loop of the tRNA, respectively. In contrast, Gly67 and Ser148 lie outside of the predicted contact surface with the tRNA. In agreement with predictions, *mj*Bud32^Arg60Glu^ inhibited while *mj*Bud32^Glu152Arg^ enhanced tRNA binding by the *mj*Cgi121–*mj*Bud32 complex (Fig. 5b). Furthermore, deletion of the C-terminal tail of Bud32 (*mj*Bud32^Arg250stop^) led to a small but reproducible reduction in tRNA binding. Consistent results were also observed using the competitive displacement assay. Specifically, tRNA was a less effective competitor of 647-CCA from the *mj*Cgi121–*mj*Bud32^Arg60Glu^ complex (IC_50_ = 7.6 µM) compared to the *mj*Cgi121–*mj*Bud32^WT^ complex (IC_50_ = 1.2 µM), whereas the opposite was observed for the *mj*Cgi121–*mj*Bud32^Glu152Arg^ complex (IC_50_ = 0.6 µM) (Fig. 5c).

Paralleling their strongly perturbed tRNA-binding activities (either positively or negatively), *mj*Bud32^Arg60Glu^ or *mj*Bud32^Glu152Arg^ inhibited the t^6^A modification activity of *ar*KEOPS, whereas *mj*Bud32^Gly67Asp^ or *mj*Bud32^Ser148Arg^ did not (Fig. 5d). Interestingly, *mj*Bud32^Arg250stop^ completely inhibited *ar*KEOPS t^6^A modification activity suggesting that the C-terminal tail of Bud32 plays an especially important role in the t^6^A catalytic cycle (Fig. 5d). Importantly, all *mj*Bud32 mutants maintained the ability to bind *mj*Cgi121 to a similar degree as *mj*Bud32^WT^ (Supplementary Fig. 5a). Thus, mutation of the predicted tRNA-binding surface on *mj*Bud32 has a measurable effect on both the tRNA-binding properties of the *mj*Cgi121–*mj*Bud32 complex and on the catalytic function of KEOPS.

In *mj*Kae1, Gln160, and Arg163 in helix α′−1 are predicted to interact with G26 and C27. Glu64 and Lys184, in contrast, reside outside of the predicted contact surface for tRNA (Fig. 5e). In *pf*Pcc1, Arg63 in helix α-2 is predicted to interact with G30, while Lys21 and Leu32 lie outside of the predicted contact surface for tRNA (Fig. 5e). Interestingly, *mj*Kae1 and *pf*Pcc1 mutations had no apparent impact on KEOPS binding to tRNA as assessed by the filter binding assay (Supplementary Fig. 5b, c) and by the competitive displacement assay using the 647-CCA probe (Supplementary Fig. 5d). However, mutations on the predicted tRNA contact surface of *mj*Kae1 and *pf*Pcc1 eliminated t^6^A modification activity, while control mutations outside of this surface did not (Fig. 5f). Importantly, all *mj*Kae1 and *pf*Pcc1 mutants associated with their corresponding binding partners in KEOPS with comparable abilities relative to their wild-type counterparts (Supplementary Fig. 5e-f). Therefore, while the predicted tRNA contact surfaces on Kae1 and Pcc1 do not perceptibly contribute to the overall affinity of KEOPS for tRNA, their integrity is critically important to support tRNA modifying activity. Together these data strongly support the notion that the KEOPS–tRNA composite model shown in Fig. 4 is representative of an enzyme–substrate complex.

Interestingly, an Arg203Asp mutation in *sc*Qri7 (analogous to the Arg163Glu mutation in *mj*Kae1 characterized above) also abolished t^6^A modification activity (Supplementary Fig. 5g). Furthermore, a Lys166Ala mutation in TsaD (analogous to the Gln160Asp mutation in *mj*Kae1 characterized above) was previously shown to inhibit tRNA binding^33^. These results suggested that the predicted tRNA contact surface in Kae1 may also be relevant for tRNA binding by its bacterial and mitochondrial orthologs. With this inference in mind, we generated models for the tRNA-bound state of a *sc*Qri7 homo-dimer (Supplementary Fig. 5h) and the bacterial *Thermotoga maritima* TsaD–TsaB–TsaE complex (Supplementary Fig. 5i) by superimpositions with Kae1 from the KEOPS–tRNA model (RMSD of 3.9 Å over 248 residues and 2.8 Å over 280 residues, respectively). The predicted tRNA-binding modes were sterically compatible with the *sc*Qri7 homo-dimer and the TsaD–TsaB heterodimer, both of which are necessary and sufficient for t^6^A catalytic function^27,34^. In contrast, the binding mode of tRNA was sterically incompatible with TsaE, which has been shown to compete with tRNA for binding to TsaD–TsaB^34^ (see “Discussion”). Therefore, although the mitochondrial and bacterial Kae1 orthologues do not rely on the CCA tail, other aspects of their tRNA-binding mechanism are likely shared in common.

Defective tRNAs are marked in cells by addition of a second CCA tail^35^ (denoted tRNA^CCAx2^) which would alter the Cgi121 binding site. Indeed, tRNA^CCAx2^ bound less efficiently than tRNA^WT^ to *mj*Cgi121 in the filter binding assay (Supplementary Fig. 6a) and was correspondingly ~4-fold less efficient at displacing the 647-CCA probe from *mj*Cgi121 (Supplementary Fig. 6b). Paralleling these results, tRNA^CCAx2^ was a less efficient substrate (~40% t^6^A modified relative to tRNA^WT^) for t^6^A modification by *ar*KEOPS and *hs*KEOPS but was an equally good substrate for *sc*Qri7 (Supplementary Fig. 6c). These observations further support the notion that recognition of the CCA tail by Cgi121 is critically important for the proper positioning of the tRNA substrate for modification by KEOPS.

### Structural stability analysis of the KEOPS–tRNA holo–enzyme complex by hydrogen–deuterium exchange (HDX)

To further explore the effect of tRNA binding on KEOPS, we carried out differential HDX experiments. tRNA induced four notable changes in KEOPS (Supplementary Fig. 6d, e). First, strongest stabilization was detected at the tRNA-binding surface of Cgi121, which supports Cgi121 as the primary tRNA-binding site. Second, weaker stabilization was observed at the Kae1–Pcc1 interface (in both proteins), which is in proximity to the predicted position of the tRNA anticodon loop. Using a fluorescence polarization assay, we did not observe an appreciable effect of tRNA on the binding affinity between Pcc1 and Kae1–Bud32–Cgi121 (Supplementary Fig. 6f). This suggested that tRNA affects an alternate property of the Kae1–Pcc1 interaction, possibly its conformation. Third, HDX de-protection was observed in the αC-β4 loop of Bud32 adjacent to the predicted contact surface for tRNA and the ATP binding pocket. Fourth, the majority of the predicted contact surfaces between tRNA and KEOPS showed no evidence of altered HDX. This may reflect the predominantly electrostatic nature of the Bud32, Kae1, and Pcc1 contacts with tRNA, which are more accessible to solvent exchange. Overall these results reveal clear effects on the extreme ends of the complex and on the more central Bud32 subunit, consistent with a tRNA-binding mode that spans the whole complex.

### Disruption of the tRNA-binding surfaces in yeast KEOPS impacts on function in vivo

**Fig. 6:**
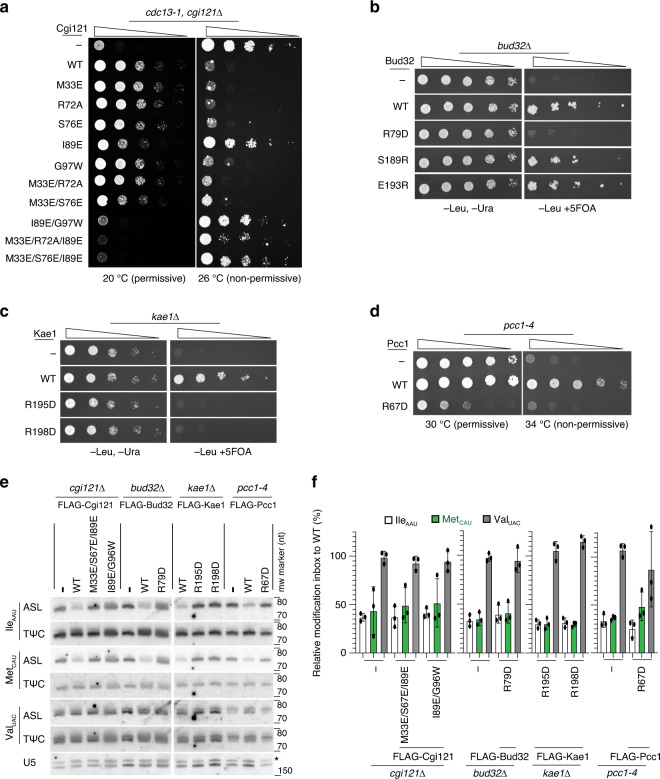
The in vivo function of yeast KEOPS depends on the predicted tRNA-binding surfaces on all four core subunits. **a** Growth analysis of a *cdc13-1*, *cgi121*Δ yeast strain transformed with an expression plasmid encoding the wild type or indicated mutant *sc*Cgi121 fused to a 2XFLAG tag. Yeast transformed with the empty parental plasmid is included as a negative control. Cells were diluted to OD_600_ = 1, subjected to five-fold serial dilutions before plating and then grown for 4 days at 20 °C or 6 days at 26 °C. **b** Growth analysis of a *bud32*Δ yeast strain transformed with an expression plasmid encoding the wild type or indicated mutant *sc*Bud32 fused to a FLAG tag. Yeast transformed with the empty parental plasmid is included as a negative control. The viability of the *bud32*Δ yeast strain is maintained by a cover plasmid encoding untagged Bud32^WT^ with a *URA3* selection marker. Cells were diluted to OD_600_ = 1, subjected to five-fold serial dilutions before plating on -leu, -ura or -leu, 5-FOA plates to counterselect for the cover plasmid. Plates were incubated for 4 days at 30 °C. **c** Growth analysis of a *kae1*Δ yeast strain transformed with an expression plasmid encoding the wild type or indicated mutant *sc*Kae1 fused to a 2XFLAG tag. Yeast transformed with the empty parental plasmid is included as a negative control. The viability of the *kae1*Δ yeast strain is maintained by a cover plasmid encoding untagged Kae1^WT^ with a *URA3* selection marker. Cells were diluted to OD_600_ = 1, subjected to five-fold serial dilutions before plating on –leu, -ura or -leu, 5-FOA plates to counterselect for the cover plasmid. Plates were incubated for 4 days at 30 °C. **d** Growth analysis of a *pcc1-4* thermosensitive yeast strain transformed with an expression plasmid encoding the wild type or indicated mutant *sc*Pcc1 fused to a FLAG tag. Yeast transformed with the empty parental plasmid is included as a negative control. Cells were diluted to OD_600_ = 1, subjected to five-fold serial dilutions before plating and then grown for 4 days at 30 °C (permissive) or at 34 °C (non-permissive). **e**, **f** Analysis of the in vivo levels of t^6^A. RNA was extracted from the cells used in panels (**a–d**) of this figure. **e** RNA was subjected to northern blot analysis using a complimentary probe targeting the anticodon stem loop (ASL) or the T loop (TΨC) of *sc*tRNA^Met^_CAU_, *sc*tRNA^Ile^_AAU_ and *sc*tRNA^Val^_CAC_. mw- molecular weight, nt- nucleotide. **f** The relative modification index was quantified by the ASL/TΨC ratio of each protein variant compared to the same ratio of cells expressing WT protein. Hybridization of a probe complementary to the U5 snRNA (U5) was used as a loading control. Shown are representative blots and quantification averages (n = 3 experiments with biologically independent samples, ±SD).

To assess the functional relevance in vivo of the KEOPS–tRNA-binding surfaces, we generated a set of mutations in *sc*KEOPS, analogous to those in *ar*KEOPS (Supplementary Table 3). The loss-of-function of individual *sc*KEOPS genes leads to a slow-growth phenotype that is a consequence of low cellular t^6^A levels^27^. *cgi121* deletion also suppresses the temperature-sensitive (ts) growth arrest phenotype of the *cdc13-1* allele by an unknown mechanism. Thus, *cdc13-1*/*cgi121*Δ cells manifest slow growth at 20 °C but can grow well at the non-permissive temperature of 26 °C. This phenotype is reversed upon expression of *sc*Cgi121^WT^ such that cells display improved growth at 20 °C but a decrease of growth at 26 °C relative to cells carrying an empty plasmid (Fig. 6a). Analysis of five single-site *sc*Cgi121 mutants (Supplementary Fig. 7a) revealed a weak change in growth pattern (slower growth at 20 °C and faster growth at 26 °C) only for the *sc*Cgi121^Ile89Glu^ mutant relative to *sc*Cgi121^WT^ (Fig. 6a). However, strong perturbations of growth were observed for the multisite mutants *sc*Cgi121^Ile89Glu/Gly97Trp^, *sc*Cgi121^Met33Glu/Arg72Ala/Ile89Glu^, and *sc*Cgi121^Met33Glu/Ser76Glu/Ile89Glu^ at both temperatures (Fig. 6a). Importantly, all of the *sc*Cgi121 mutants tested were expressed at levels equal to or higher than *sc*Cgi121^WT^ (Supplementary Fig. 7b).

Two *sc*Bud32 mutations, Arg79Asp and Glu193Arg, were predicted to disrupt protein function (Supplementary Fig. 7c). Following loss of a covering plasmid on 5-FOA—containing medium, *bud32*Δ cells expressing *sc*Bud32^Arg79Asp^ but not scBud32^Glu193Asp^ displayed a strong growth defect relative to cells expressing *sc*Bud32^WT^ (Fig. 6b). Cells expressing the *sc*Bud32^Ser189Arg^ mutant, predicted not to alter tRNA binding, displayed growth similar to cells expressing *sc*Bud32^WT^. Importantly, all three *sc*Bud32 mutants were expressed at levels comparable to *sc*Bud32^WT^ (Supplementary Fig. 7d).

Two *sc*Kae1 mutations, Arg195Asp and Arg198Asp (Supplementary Fig. 7e), and the *sc*Pcc1 mutation Arg67Asp (Supplementary Fig. 7f) were predicted to disrupt protein function. Fully consistent with expectations, following loss of a covering plasmid on 5-FOA—containing medium, *kae1*Δ cells expressing *sc*Kae1^Arg195Asp^ or *sc*Kae1^Arg198Asp^, displayed strong growth defects compared to cells expressing *sc*Kae1^WT^ (Fig. 6c), despite comparable expression levels (Supplementary Fig. 7g). Likewise, *pcc1-4* ts cells^6^ expressing *sc*Pcc1^Arg67Asp^ displayed growth defects at the non-permissive temperature of 34 °C (Fig. 6d) despite expression levels comparable to *sc*Pcc1^WT^ (Supplementary Fig. 7h). Furthermore, expression of *sc*Pcc1^Arg67Asp^ also led to a growth defect at the permissive temperature of 30 °C, suggesting that the *sc*Pcc1^Arg67Asp^ mutant acts in a dominant negative manner over the endogenous allele.

We next used a “positive hybridization in the absence of modification at A37” (PHA37) assay^4,36^ to correlate growth defect phenotypes to t^6^A catalytic activity. In this assay, the t^6^A modification is detected by reduced annealing of complementary oligonucleotide probes directed at the anticodon loop of substrate tRNAs, normalized to signal from probes that anneal to the TΨC loop. Fully consistent with the growth phenotypes, we observed that *cgi121*Δ cells, *bud32*Δ cells, *kae1*Δ cells and also *pcc1-4* cells that express proteins with mutations on the tRNA contact surfaces, exhibited reduced t^6^A modification levels of endogenous *sc*tRNA^Ile^_AAU_ and *sc*tRNA^Met^_CAU_ substrates relative to the corresponding parental cells expressing wild-type proteins (Fig. 6e-f). The reduced t^6^A modification levels were comparable to cells carrying an empty plasmid, with the exception of *kae1*Δ cells that we were not able to isolate sufficient RNA for analysis (due to the slow growth phenotype). Importantly, hybridization of probes to the anticodon region of *sc*tRNA^Val^_UAC_, which is not a KEOPS substrate, was unaffected by the presence or absence of functional KEOPS subunits (Fig. 6e). HPLC nucleoside analysis of total tRNA extracts corroborated the findings of diminished t^6^A levels observed for mutant KEOPS subunits in the PHA37 experiments (Supplementary Fig. 7i). Overall, the close correlation of the growth defects and t^6^A modification phenotypes led us to conclude that the tRNA-binding mechanism uncovered for *ar*KEOPS has predictive relevance for the function of eukaryotic KEOPS subunits in vivo.

### Cgi121 can bind to tRNA in yeast independent of KEOPS

The finding that archaeal Cgi121 as an individual subunit expressed in bacteria co-purifies with tRNA led us to question whether the orthologous *sc*Cgi121 protein could also bind tRNA independent of KEOPS in vivo. Due to a linear binding architecture, removal of Bud32 disrupts Cgi121 association with KEOPS. FLAG-*sc*Cgi121^WT^ immune-precipitated from *bud32*Δ cells was able to pull down tRNA^Ile^_AUU_, while the *sc*Cgi121^I89E/G96W^ mutant deficient for CCA tail binding was not (Supplementary Fig. 7j-k). This result supports the possibility that Cgi121 can exist in complex in vivo with tRNA independent of other KEOPS subunits.

### The binding of ANN-decoding and Ala tRNAs to KEOPS regulates the ATPase activity of Bud32

**Fig. 7:**
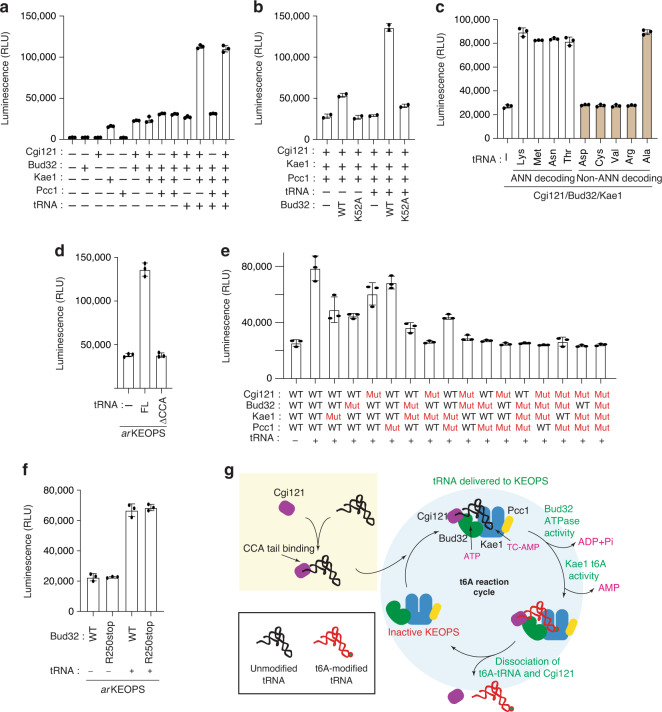
The ATPase activity of Bud32 is potentiated by tRNA binding. **a**–**f** ATPase activity analysis of the indicated archaea KEOPS proteins in the presence and absence of *mj*tRNA^Lys^_UUU._ Activity was monitored using the ADP Glo assay. Displayed results represent the average luminescence (*n* = 3 independent experiment samples, ±SD) for each reaction condition. **c** In addition to *mj*tRNA^Lys^_UUU_, the effect the alternate KEOPS substrates *mj*tRNA^Met^_CAU,_ *mj*tRNA^Asn^_GUU_, *mj*tRNA^Thr^_GGU_, and the KEOPS non-substrates *mj*tRNA^Asp^_GUC_, *mj*tRNA^Cys^_GCA_ *mj*tRNA^Val^_CAC,_ *mj*tRNA^Arg^_GCG_, and *mj*tRNA^Ala^_GGC_ on ATPase activity were also examined. **d** In addition to wild-type *mj*tRNA^Lys^_UUU_, the effect of *mj*tRNA^Lys^_UUU_^ΔCCA^ on ATPase activity was also examined. **e** The mutant KEOPS proteins denoted by Mut (in red) are *mj*Cgi121^Met60Glu^, *mj*Bud32^Arg60Glu^, *mj*Kae1^Arg163Asp^ and *pf*Pcc1^Arg63Asp^. **g** A model for the KEOPS t^6^A catalytic cycle. Cgi121 can bind tRNA independent of other KEOPS subunits (yellow box). Binding of the Cgi121–tRNA complex to the Bud32–Kae1–Pcc1 complex delivers the tRNA substrate to KEOPS (blue circle). Binding of tRNA to KEOPS (and minimally the Cg121–Bud32–Kae1 subcomplex) activates the ATPase activity of Bud32 which in turns activates the t^6^A modification activity of Kae1. Dissociation of Cgi121 and t^6^A-modified tRNA from KEOPS completes a catalytic cycle.

Bud32 enhances tRNA binding in a non-catalytic manner (Figs. 1b, 3e, and 3g). However, its ATPase activity is also required for KEOPS function in vitro and in vivo^11,22,23^. To better understand the role of Bud32 in KEOPS, we investigated the effect of tRNA on Bud32 ATPase activity. Consistent with prior findings^23^, *mj*Bud32 alone displayed very low ATPase activity that was robustly potentiated by *mj*Cgi121 (~10 fold) (Fig. 7a), while *mj*Kae1 and *pf*Pcc1 had marginal additional effects on ATPase activity. Interestingly, while tRNA did not affect the ATPase activity of the *mj*Bud32–*mj*Cgi121 complex, it caused a marked (~5 fold) potentiation of the ATPase activity of both *mj*Kae1*–mj*Bud32*–mj*Cgi121 and the full KEOPS complex (Fig. 7a). Importantly, *ar*KEOPS containing a catalytically dead *mj*Bud32^Lys52Ala^ mutant displayed background ATPase activity that was not potentiated by tRNA, indicating that the measured elevated activity resulted from *mj*Bud32 (Fig. 7b).

Next, we tested if *mj*Bud32 activation is restricted to KEOPS substrates (ANN-decoding tRNAs). Interestingly, *mj*Bud32 ATPase activity was potentiated by all four ANN-decoding tRNAs verified as KEOPS substrates (Supplementary Fig. 8a) to a similar level but only by one of five non-ANN-decoding tRNAs (specifically *mj*tRNA^Ala^_GGC_) (Fig. 7c). Thus, KEOPS substrate tRNAs appear more likely to potentiate the ATPase activity of Bud32. We therefore examined if mutation of the 36-UAA-38 element, a motif in the tRNA anticodon loop that is required for t^6^A modification^28,37^, would adversely affect the activation of Bud32 ATPase by *mj*tRNA^Lys^ (Supplementary Fig. 8b). Surprisingly, no effect on ATPase activity was observed suggesting that determinants for Bud32 activation lie elsewhere in the tRNA structure. Sequence alignment of the 5 *M. jannaschii* tRNAs that activated Bud32 ATPase activity (Lys, Met, Asn, Thr, and Ala tRNAs) revealed conservation of a common CU element at positions 10 and 11, an element that is not present in the tRNAs that did not activate ATPase activity (Supplementary Fig. 8c left panel). Expansion of the comparison to all 9 available ANN-decoding tRNA sequences from *M. jannaschii* together with three Ala tRNA sequences revealed a strong conservation of the CU element (C10 in 12/12 and U11 in 10/12 sequences) that is clearly lacking in the other 23 tRNA sequences (C10 in 6/23 sequences; U11 in 2/23 sequences; a CU combination in 0/23 sequences) (Supplementary Fig. 8c middle panel). A similar analysis with yeast tRNAs revealed a very strong conservation of C10 within ANN-decoding and Ala tRNAs (C10 in 108/108 sequences relative to C10 in 72/173 of all other tRNA sequences) while position 11 showed no clear conservation for any particular nucleotide (Supplementary Fig. 8c right panel). Interestingly, the 10-CU-11 element is predicted to contact Bud32 in the KEOPS–tRNA model (Supplementary Fig. 8d). Demonstrating a potential functional role for the 10-CU-11 element in Bud32 ATPase activation, reversal 10-UC-11 mutations in all five tRNAs tested inhibited ATPase activation (Supplementary Fig. 8e) as well as inhibition of t^6^A modification of the four ANN-decoding tRNAs (Supplementary Fig. 8f). Suggesting that the 10-UC-11 reversal mutations did not adversely affect tRNA fold, two of the 10-UC-11 mutants examined (namely *mj*tRNA^Lys^ and *mj*tRNA^Asn^) maintained the ability to displace the 647-CCA probe from KEOPS, (Supplementary Fig. 8g).

### Bud32 activation and t^6^A modification rely on the same tRNA-binding mechanism

We hypothesized that the t^6^A activity of Kae1 and the activation of the ATPase activity of Bud32 within KEOPS (or minimally the *mj*Kae1*–mj*Bud32*–mj*Cgi121 subcomplex) rely on the same higher order binding mode of tRNA illustrated in Fig. 4a. In support of this idea, tRNA^ΔCCA^, which cannot bind to KEOPS, did not potentiate the ATPase activity of *ar*KEOPS (Fig. 7d) and *hs*KEOPS (Supplementary Fig. 9a) and the CCA-oligo, which only contacts Cgi121, did not potentiate the ATPase activity of *ar*KEOPS (Supplementary Fig. 9b). Also, KEOPS reconstituted with tRNA contact surface mutants (either *mj*Cgi121^Met60Glu^, *mj*Bud32^Arg60Glu^, or *mj*Kae1^Arg163Asp^) displayed reduced ATPase activation by tRNA (Fig. 7e). Furthermore, *ar*KEOPS reconstituted with a combination of mutated subunits were completely refractory to ATPase activation by tRNA. Thus, we conclude that the ATPase and t^6^A modification activities rely on a shared tRNA-binding mechanism. Interestingly, the ATPase activity of KEOPS with a Bud32 mutant lacking its C-terminal tail was still fully responsive to tRNA (Fig. 7f) but KEOPS with this Bud32 variant displayed no t^6^A activity in vitro (Fig. 5d) and could not support biological function in vivo^23^. The significance of this outlier behavior is elaborated on below (see Discussion).

Previous reports suggested that Bud32 ATPase activity serves to regulate substrate release from KEOPS^22^. Accordingly, *ar*KEOPS harboring catalytically dead *mj*Bud32 mutants should produce a mole equivalence of t^6^A-modified tRNA. Likewise, *mj*Bud32 binding to a non-hydrolysable ATP analog (AMP-PnP) or to ADP (in contrast to ATP) should in principle stabilize KEOPS in a tRNA-bound state. Contrary to expectations, *ar*KEOPS with catalytically dead *mj*Bud32 mutants (Lys52Ala or Asp161Arg) had no detectable t^6^A activity (Supplementary Fig. 9c-d). Furthermore, ADP and AMP-PnP, had no discernable effect on the tRNA-binding activity of KEOPS or the Cgi121–Bud32–Kae1 subcomplex relative to ATP, as determined by the filter binding assay and by the 647-CCA probe displacement assay (Supplementary Fig. 9e-f). These results suggest that the ATPase activity of Bud32 is not involved in substrate release but rather serves an alternate essential function in the catalytic cycle of KEOPS, prior to the t^6^A reaction (Fig. 7g).

## Discussion

We have discovered that Cgi121 plays an evolutionarily conserved role in the efficient recruitment of substrates to KEOPS through binding to the 3′ CCA tail of tRNAs. We show that the recruited tRNA is engaged through an extended binding surface, which involves all four core KEOPS subunits, that is remarkably complementary in shape to that of the tRNA. The tRNA-binding surface plays an essential role in regulating the ATPase activity of Bud32 and in the t^6^A modification reaction.

Based on our mechanistic findings, we propose the following model of the KEOPS t^6^A catalytic cycle (Fig. 7g): Step 1: Cgi121 can form a complex with tRNA independent of other KEOPS subunits. Step 2: Cgi121 delivers tRNA to the remaining KEOPS subunits (Bud32–Kae1–Pcc1). We cannot rule out the possibility that KEOPS is preassembled prior to tRNA binding or that the Cgi121–Bud32 binary complex, which binds tRNA with higher avidity than Cgi121, also acts to independently bind tRNA. However, since Bud32 is fused to Kae1 in some species (for example *M. jannaschii*), it is unlikely to function independently of Kae1. Within KEOPS, Bud32 reinforces tRNA binding by Cgi121 and all four core KEOPS subunits assist to position the anticodon loop into the catalytic cleft of Kae1. Step 3: Kae1 carries out the t^6^A modification reaction on the correctly oriented tRNA. Step 4: t^6^A-modified tRNA, alone or in complex with Cgi121, dissociates from KEOPS allowing a new catalytic cycle to begin.

How does the Bud32 ATPase activity fit into the t^6^A catalytic cycle of KEOPS? A model consistent with the data presented here envisions that the ATPase activity of Bud32 serves to regulate the catalytic function of its binding partner Kae1. Thus, Bud32 catalytic activity occurs before that of Kae1 in the t^6^A catalytic cycle and ATPase activity would be required for every t^6^A turnover event. How is Bud32 ATPase activity communicated to influence the t^6^A modification activity of Kae1 remains an open question. One tantalizing possible mechanism involves the highly conserved^23,38^ C-terminal tail of Bud32. In Bud32–Kae1 structures, this tail resides in the catalytic cleft of Kae1^23,39^ and is thus well placed to influence t^6^A modifying activity. This tail element is dispensable for the potentiation of Bud32 ATPase activity by tRNA (Fig. 7f) but is required for the catalytic function of Kae1 in vitro (Fig. 5d) and in vivo^23^. We therefore reason that the tail of Bud32 exerts a positive influence on Kae1 but only in response to Bud32 ATPase activity following tRNA binding. The details of how exactly this could occur awaits further study. Interestingly, similar to the tail region of Bud32, Pcc1 is not required for tRNA binding by KEOPS or for the activation of Bud32 by tRNA, but it is required for tRNA modification by KEOPS (Supplementary Fig. 1b). While the precise role of this subunit also remains an open question, we speculate that based on the proximity to both Kae1 and tRNA, Pcc1 and perhaps the Bud32 tail may help guide A37 to the active site of Kae1. The tRNA-binding model presented here may provide a useful framework for further mechanistic analyses.

Our data also illustrate intricate allosteric interactions between KEOPS subunits and substrate tRNA (summarized in Supplementary Fig. 9g). Accordingly: (i) Binding between Cgi121 and Bud32 reciprocally leads to the activation of Bud32 ATPase activity and to the potentiation of Cgi121 tRNA-binding activity. (ii) Binding of tRNA to the Cgi121–Bud32–Kae1 subcomplex or to the Cgi121–Bud32–Kae1–Pcc1 complex further activates the ATPase activity of Bud32. (iii) Binding of tRNA to KEOPS alters the dynamics of the Pcc1–Kae1 interface and within Bud32 in close proximity of the ATPase active site. (iv) The ATPase activity of Bud32 is somehow coupled to the ability of Kae1 to modify tRNA by an as-yet undefined mechanism.

What is the utility of the added complexity of Kae1 relative to Qri7? tRNA processing events frequently occur in a hierarchical manner^40,41^. One possible advantage of the added complexity could be the temporal integration of the t^6^A modification with other tRNA processing events. By virtue of how KEOPS operates, this would entail the t^6^A modification occurring downstream of CCA tail addition, and likely prior to the charging of the tRNA with its respective amino acid (due to steric constraints). Also, because tRNA introns are located immediately 3′ to the anticodon, physically separating U36 and A37^42^ and disrupting the UAA-motif characteristic of KEOPS substrates^28,37^, it is likely that t^6^A modification occurs only after tRNA splicing.

An alternate possible advantage for added complexity is to allow the precise control of t^6^A levels in cells as a means to regulate gene expression. Whereas Qri7 activity appears to be limited by substrate availability^27,37^, the activity of Kae1 depends on the full composition of KEOPS and on the ATPase activity of Bud32. Each of these dependencies affords an opportunity for regulating KEOPS activity. A candidate regulation mechanism apparent from our study is the control of Cgi121 protein levels, to which KEOPS activity is highly responsive. In support of this proposed mechanism, yeast Cgi121 is an unstable protein with a half-life of ~30 min^23^ and its expression levels are sub-stoichiometric with respect to other KEOPS subunits^43^. Reduced t^6^A levels have been proposed to modulate a translational program in yeast in response to amino acid deprivation, primarily by selectively inhibiting the translation of ~300 mRNAs enriched with Arg AGA/G codons^44^. In support of the possibility for regulatory control, there are precedents for the regulation of translation by the modulation of tRNA modifications at position 34, which directly affects wobble position interactions with the codon^45,46^, and at position 37. For example, modulation of m^1^G37 levels is implicated in the control of Mg^2+^ homeostasis in *Salmonella enterica*, which greatly impacts virulence^47,48^. Likewise, modulation of i^6^A37 levels impacts global translation profiles and cellular metabolism in fission yeast^49^.

Interestingly, Qri7 and TsaD have not evolved a mechanism to recognize the CCA tail of tRNA, which raises the important question of why KEOPS specifically functions in this manner. However, mutational analyses reported here and elsewhere^33^ raises the enticing possibility that other aspects of tRNA binding by Kae1 have relevance for how Qri7 and TsaD recognize tRNA. Although Qri7 and TsaD do not rely on the CCA tail for function (Fig. 3h, i, Supplementary Fig. 5h, i), mutation of their predicted tRNA contact surfaces involving helix-α′1 adversely affected aspects in both enzyme’s activities (Supplementary Fig. 5g; ref. ^33^). Furthermore, modeling of tRNA binding to the Qri7 homo-dimer and TsaD–TsaB dimer reveals no steric conflicts (Supplementary Fig. 5h-i). In addition, modeling of tRNA binding to the TsaD–TsaB heterodimer instead revealed a potential role for helix-α7 of TsaB in tRNA contact (Supplementary Fig. 5i), possibly accounting for the finding that deletion of this helix prevented tRNA binding by TsaD–TsaB^33^. Our composite substrate-binding model might also explain the observation that TsaE competes with tRNA for binding to the TsaD–TsaB complex^34^ as the two molecules are predicted to sterically clash (Supplementary Fig. 5i). Thus, we posit that the model of KEOPS bound to tRNA could provide a useful platform for understanding and deciphering substrate binding and regulatory mechanisms for other t^6^A modifying enzymes.

## Methods

### Protein expression, purification and mutagenesis

For bacterial expression, plasmids were transformed into BL21-CodonPlus DE3-RIL *E. coli* (Agilent Technologies) grown in TB or M9 minimal media supplemented with ^15^N-NH_4_Cl (Cambridge Isotopes) for NMR spectroscopy purposes. Expression was induced by the addition of 0.3 mM IPTG to bacterial cultures at OD_600_ = 0.8–1 at 18 °C overnight.

The *M. jannaschii* Cgi121, Kae1, *mj*1130 (fusion Kae1-Bud32 protein), as well as *P. furiosus* Pcc1 and human TPRKB were expressed with the pGEX2T vector as GST fusion proteins with a TEV cleavage site between the GST and protein of interest coding regions. Cell pellets from overnight cultures were lysed via homogenization in lysis buffer (50 mM HEPES pH 7.5, 500 mM NaCl, 5 mM EDTA, 2 mM DTT) supplemented with 0.5 mM PMSF. Cleared lysates were run over GST-sepharose columns at room temperature. Bound protein fraction was eluted by TEV cleavage, followed by purification with an S200 sizing column (20 mM HEPES pH 7.5, 100 mM NaCl, 2 mM DTT).

The *M. jannaschii* Bud32 and *S. cerevisiae* Qri7 (residues 30-407) were expressed with pProEx plasmids as N-terminal hexahistidine-tagged proteins with a TEV cleavage site between the His-tag and protein-coding regions. Cells were lysed as described above in lysis buffer (25 mM HEPES pH 7.5, 500 mM NaCl, 25 mM imidazole). Lysates were run over a Ni-NTA column, bound fraction was eluted with a gradient of lysis buffer with 250 mM imidazole. Eluted fractions were pooled and dialyzed to reduce imidazole concentration to 25 mM in parallel to TEV cleavage, followed by subtraction of His-tagged TEV with an Ni-NTA column. Flow through protein fraction was further purified by an S200 sizing column (20 mM HEPES pH 7.5, 100 mM NaCl, 2 mM DTT).

Human PRPK was expressed with a pETM30 plasmid as N-terminal GST and hexahistidine-tagged protein with a TEV cleavage site between the His-tag and protein-coding region. TPRKB and PRPK expression plasmids were co-transformed into BL21 cells and the complex was co-purified by an Ni-NTA column purification followed by GST column as described above followed by TEV cleavage and sizing columns at 4 °C as described above.

The human 4-subunit KEOPS complex was expressed in Sf9 cells through a baculovirus expression system using the pACEBac-TPOLC plasmid^7^ and co-purified with His-tagged LAGE3 via an Ni-NTA column and S200 sizing column as described above.

The *E. coli* TsaB, and TsaE proteins were expressed in bacteria as previously described^18^ using the pCDII29 (TsaB), pCDII30 (TsaE) plasmids. *E. coli* TsaD was expressed in bacteria as an N-terminal hexahistidine-SUMO-tagged protein using the pCD174 plasmid^18^. Cells were grown in 250 mL overnight at room temperature to OD_600_ = 2–3 and were then pelleted and resuspended in 0.5 L LB and grown for 1 h at 20 °C (TsaD) or 37 °C (TsaB and TsaE). Expression was induced by addition of 1 mM IPTG to cultures and incubation for 4 h at 37 °C (TsaB and TsaE) or overnight at 20 °C (TsaD). TsaD expressing cultures were also supplemented with 0.5 µM ZnCl_2_. Following expression, cells were then pelleted and resuspended in lysis buffer (100 mM Tris pH 8, 2 mM 2-mercaptoethanol, 20 mM imidazole, 1 mM PMSF, 10% glycerol, and 100 mM KCl or 300 mM KCl for TsaD expression) and were lyzed by sonication. Lysates were run over a Ni-NTA column, bound fraction was eluted with lysis buffer (without PMSF) containing 200 mM imidazole. Fractions containing purified proteins were pooled and concentrated and dialyzed against 100 mM Tris pH 8, 2 mM 2-mercaptoethanol and 100 mM KCl or 300 mM KCl (TsaD) at 4 °C. In parallel to dialysis the N-terminal hexahistidine-SUMO tag of the TsaD protein was cleaved by addition of ULP1. TsaD samples were then applied to a pre-equilibrated Ni-NTA column. TsaD fractions were dialyzed overnight against 100 mM Tris pH 8, 2 mM 2-mercaptoethanol and 300 mM KCl at 4 °C. Before freezing at −80 °C, protein samples were supplemented with glycerol to a final concentration of 20%.

For all proteins and tRNAs, point mutations we generated by site-directed mutagenesis using standard protocols (for a full list of primers used in this study, see Supplementary Table 4).

### T7 in vitro transcription and purification of tRNA

tRNA sequences were obtained from GtRNAdb website (http://gtrnadb.ucsc.edu/)^50^ (a list of tRNA sequences used in this study are provided in Supplementary Table 5) and were synthesized and subcloned into a pUC19 plasmid fused at the 5′ end to a T7 promoter and at the 3′ end to a hepatitis delta virus ribozyme followed by a BamHI site. 250 µg plasmid DNA was linearized by BamHI digestion overnight and used as a template for T7 run-off transcription reaction (100 mM Tris-Cl pH 8.0, 4 mM ATP/GTP/CTP/UTP, 10 mM DTT, 1 mM spermidine, 0.1% Triton X-100, 25 mM MgCl_2_, 0.2 mg/mL T7 RNA polymerase, 10 U/mL thermostable inorganic phosphate (NEB) and 200 U/mL RiboLock (Thermo Scientific)) at 37 °C for 4 h. Nucleic acids were purified from transcription reaction by phenol-chloroform extractions followed by ethanol precipitation. Pellets were washed with 80% ethanol and air dried followed by solubilization with 5 mL 8 M urea. RNA was refolded in 45 mL of 10 mM Bis-Tris-Cl pH 6.5, 10 mM MgCl_2_. tRNA was purified on a 5 mL Q column (GE Healthcare) using DEPC-treated water (buffer A) and 2 M NaCl (buffer B) starting with 4 column volumes at 25% B followed by a linear gradient over four column volumes from 25% to 40% B. Fractions containing tRNA were analyzed via electrophoresis on TBE-urea polyacrylamide gels were pooled and treated with one volume of isopropanol to precipitate RNA. The precipitated RNA was washed with 80% ethanol and then air dried. The RNA pellet was resuspended in 10 mM Tris-Cl pH 8. Prior to use, the tRNA was refolded by (i) boiling at 95 °C for 2 min, (ii) flash cooling on ice, (iii) warming to 50 °C, (iv) addition of MgCl_2_ to a final concentration of 2 mM and (v) slow cooling to room temperature.

### In vitro t^6^A assays

In vitro t^6^A reactions were done at room temperature for *sc*Qri7, at 30 °C for *hs*KEOPS, 37 °C for *ec*TsaBDE and at 55 °C for *ar*KEOPS. Reactions where typically 20 min unless stated otherwise. Qri7 t^6^A reactions were done using 50 mM Tris-HCl, pH 8.0, 200 mM NaCl, 1 mM DTT, 1 mM threonine, 1 mM NaHCO_3_, 2 mM ATP, 5 mM MnCl_2_, 2 μM of Sua5/Qri7 and 80 μM of tRNA. *hs*KEOPS t^6^A reaction were done using 25 mM Tris-Cl pH 8, 150 mM NaCl, 5 mM DTT, 5 mM MgCl_2_, 1 mM threonine, 1 mM NaHCO_3_, 4 mM ATP, 5 mM spermidine and 0.5 µl thermostable inorganic pyrophosphatase (TIPP, NEB), 5 μM *sc*Sua5, 2 μM 4-subunit *hs*KEOPS and 80 μM tRNA. *ec*TsaDBE t^6^A reactions were done using 100 mM Tris-Cl pH 8, 300 mM KCl, 5 mM DTT, 5 mM MgCl_2_, 10 mM threonine, 10 mM NaHCO_3_, 2 mM ATP, 5 μM *sc*Sua5, 2.5 μM *ec*TsaDBE and 80 μM tRNA. *ar*KEOPS t^6^A reactions were done with 50 mM Tris–HCl pH 8.0, 150 mM NaCl, 2.5 mM DTT, 0.5 mM threonine, 0.5 mM NaHCO_3_, 2 mM ATP, 0.25 mM MnCl_2_, 0.25 mM MgCl_2_, 2.5 mM spermidine, 0.5 µL TIPP, 2 μM of *mj*Sua5/*mj*Cgi121/*mj*Kae1/*mj*Bud32, *pf*Pcc1 and 80 μM of tRNA.

### HPLC analysis of tRNA modifications

RNA was enzymatically digested according to ref. ^51^ by adjusting the volume to 50 µL, adding 10 µL of Nuclease P1 at 0.2 U/mL (Sigma, N8630) and 5 mL ZnSO_4_. Digestion reaction proceeded overnight at room temperature followed by dephosphorylation of nucleotides using 1 µL calf intestinal phosphatase (CIP, NEB) for 2 h at 37 °C. The resulting mononucleosides were analyzed using a Discovery C18 (15 cm × 4.6 mm, 5 µM) reverse-phase column (Supelco Analytical) on a Dionex Ultimate 3000 HPLC Unit (Thermo Scientific) with a linear gradient of 98:2 to 87.5:12.5 of 250 mM ammonium acetate pH 6.5 and 40% acetonitrile and data analysis was performed using the Chromeleon HPLC software v6.8. Data graphs were generated using GraphPad Prism v8.3 (GraphPad).

### tRNA labeling and filter binding assay

20 µM tRNA was 5′ dephosphorylated using 1 µL CIP (NEB) in NEB buffer 3 at a volume of 20 µL at 50 °C for 1 h. Dephosphorylated tRNA was purified using a standard phenol-chloroform extraction protocol and ethanol precipitation. The tRNA pellet was resuspended and labeled in a final volume of 30 µL with 1.5 µl T4 PNK and 30 µCi ATP-γ-^32^P (Perkin Elmar) for 30 min at 37 °C. Reactions were terminated by adding 1.5 µL 0.5 M EDTA pH 8 and heating at 70 °C for 10 min. Excess ATP was removed by a mini Quick Spin Column (Roche). Before use in binding assays, tRNA was reheated to 70 °C for 5 min and cooled back to room temperature for 10 min.

Binding reactions, unless indicated otherwise, were done at room temperature for 1 h at 50 µL with 50 nM labeled tRNA, 20 mM HEPES pH 7.5, 100 mM NaCl and 2 mM DTT. Reactions were loaded on a nitrocellulose membrane using a 96-well vacuum-operated filtration apparatus. The membrane was washed four times with 100 µL of 20 mM HEPES pH 7.5, 100 mM NaCl and 2 mM DTT and exposed to a PhosphorImager plate for 2–8 h. The plate was then scanned and visualized with a PhosphorImager (Molecular Dynamics).

### NMR experiments

NMR-heteronuclear single quantum coherence spectroscopy (HSQC) spectra were recorded at 25 ˚C on a 600-MHz Bruker AVANCE III spectrometer. The 600-MHz spectrometer was equipped with a 1.7-mm TCI CryoProbe. All NMR samples contained 150 µM of ^15^N-*mj*Cgi121 in 20 mM HEPES pH 7.5, 100 mM NaCl, 2 mM DTT and 10% D_2_O. Data processing was conducted using NMRviewJ v9.2.0.b11 and NMRpipe v20180523^52^ and NMR spectra were analyzed using Analysis v2.4.2^53^. Backbone resonance assignments for *mj*Cgi121 have been previously reported^23^ (BMRB: 15981). In all of the peak intensity analyses, HSQC peak heights were used. (^1^H, ^15^N) Chemical shift perturbations (CSPs) were calculated as a weighted average Δδ_av_ = [(Δδ_H_)^2^ + (Δδ_N x_ 0.15)^2^]^1/2^.

### Crystallization, data collection, and structure determination

Crystals of *mj*tRNA^Lys^_UUU_ were grown by mixing *mj*tRNA^Lys^_UUU_ at 600 µM in buffer (10 mM Tris pH 8 and 2 mM MgCl_2_) with an equal volume of 100 mM MES pH 6.0, 0.2 M ZnAcetate, 10% PEG8000. Crystals were grown in hanging drops at 20 °C and were cryoprotected with 20% glycerol and flash frozen in liquid N_2_. Diffraction data were collected at CLS-08-ID-1 and processed using XIA2-DIALS v1.14.5^54^. Molecular replacement was performed by Phaser v2.8.3^55^ using tRNA^Phe^ (PDB: 3L0U) as a search model. Model building and refinement was performed using Coot v0.9 and Phenix v1.16^56,57^. Crystals of the *mj*Cgi121–*mj*tRNA^Lys^_UUU_ complex were grown by mixing 600 µM of *mj*Cgi121 and *mj*tRNA^Lys^_UUU_ in buffer (20 mM HEPES pH 7.5, 100 mM NaCl, 2 mM DTT, 10 mM Tris pH 8 and 2 mM MgCl_2_) with an equal volume of 100 mM HEPES pH 7.5, 200 mM LiSO_4_ and 25% PEG 3000. Crystals were grown in hanging drops at 20 °C and were cryoprotected with 20% glycerol and flash frozen in liquid N_2_. Diffraction data were collected at APS IMCA-17ID and processed using AutoProc v1.0.5^58^. Molecular replacement was performed using Phaser v2.8.3 using *mj*Cgi121 (PDB:3ENH) and *mj*tRNA^Lys^_UUU_ as search models. Model building and refinement was performed using Coot v0.9 and Phenix v1.16.

### Pulldown assays

GST-pull down was done using 5 µg of GST-*mj*Cgi121 mixed with 5 µg of the target non-tagged proteins in 1 mL binding buffer (50 mM HEPES pH 7.5, 500 mM NaCl2, 5 mM EDTA, 2 mM DTT) supplemented with 25 µL of pre-equilibrated GSH-sepharose beads. Binding was allowed to proceed for 1 h at room temperature with mild agitation, followed by three rounds of spin-downs and 1 ml washes in binding buffer. Beads were resuspended in SDS PAGE loading buffer and supernatants were analyzed by gel electrophoresis.

His-pull downs were done in essentially the same manner, using 2 µg of His-*mj*Bud32 mixed with 2 µg of the target non-tagged proteins in 1 mL binding buffer (50 mM HEPES pH 7.5, 500 mM NaCl, 25 mM imidazole) supplemented with 25 µL of pre-equilibrated Ni-NTA-sepharose beads.

### Fluorescence polarization assays

The 647-CCA RNA probe was generated by fusion of an Alexa-647 fluorescent tag to the 5′ of the 5′-CCGCCA-3′ oligonucleotide (IDT Inc.). Proteins and RNA probes were mixed in FP buffer (20 mM HEPES pH 7.5, 100 mM NaCl, 2 mM DTT) in a 384-well flat bottom black plate (Corning 3573) at a final volume of 25 µL. For *K*_d_ extraction, the RNA probe was kept at 50 nM throughout experiments and each analyzed protein was added to the concentrations indicated in each figure. Unless stated otherwise, each data point was derived from a technical duplicate and each binding curve was performed in triplicate. For the competitive displacement assay, the RNA probe (50 nM) and protein concentrations (*mj*Cgi121 at 1.5 µM, *mj*Cgi121–*mj*Bud32 complexes at 0.8 µM, *hs*KEOPS at 4 µM) were kept constant throughout experiments, and non-fluorescent RNA molecules were added to final concentrations indicated in each figure. Fluorescence polarization measurements were done on BioTek Synergy Neo plate reader (BioTek) using Gen5 v2.05 software with excitation and absorbance at 620/680 nm, respectively. Binding graphs and the derived binding constants were generated using GraphPad Prism v8.1.2 and v8.3 (GraphPad). Error bars on binding graphs represent deviation between duplicate data points. *K*_d_ and IC_50_ SD values represent deviation between values obtained from three binding experiments.

The *pf*Pcc1 probe was generated by labeling 200 µM *pf*Pcc1 in 1 mL HEPES 20 mM pH 7.5, 100 mM NaCl, 2 mM DTT supplemented with 200 µM NHS-Fluorescein (Thermo Scientific 46410) at room temperature overnight. Excess labeling reagent was quenched and removed by dialysis against 1 L 50 mM Tris pH 7.5, 100 mM NaCl, 2 mM DTT followed by a S200 sizing column with 20 mM HEPES pH 7.5, 100 mM NaCl, 2 mM DTT. Fluorescent *pf*Pcc1 was kept at 50 nM throughout experiments, and all other protein concentrations are indicated in each figure. *mj*tRNA^Lys^, when added, was at 20 µM. Fluorescence polarization measurements were done on a BioTek Synergy Neo plate reader (BioTek) using Gen5 v2.05 software with excitation and absorbance at 485/528 nm, respectively. Binding graphs and the derived binding constants were generated using GraphPad Prism v8.3 (GraphPad).

### tRNA docking and molecular modeling

A model of the archaeal KEOPS complex model was built as previously described^23^, by structural alignment of previously solved binary and ternary subcomplexes of archaea KEOPS (PDB ID: 3ENO and 3ENH). Rigid body docking of the tRNA body and the KEOPS complex model was carried out by the Patchdock server v1.3^59^ (https://bioinfo3d.cs.tau.ac.il/PatchDock/index.html). Distance constraints were assigned to residues 74–76 of the tRNA (CCA tail) and residues Gln69, Gly78 from Cgi121 subunit which are within the tRNA–Cgi121 interface. The binding pose was exhaustively searched by using shape complementarity principles integrated in the Patchdock v1.3 docking algorithm. The resulting candidate complexes were filtered and ranked according to a geometric shape complementarity score. The best-docking model was then energy minimized using steepest descent algorithm in Gromacs 5.1.4 molecular simulation package.

### Immunoblot analysis

Yeast cell lysates were prepared using the TCA method. 30 mg of total protein from each sample was subjected to SDS-PAGE and immunoblotting using the anti-DDDDK tag (abcam, ab1257) and the anti-pGK1 (abcam, ab113687) antibodies at a 1:2,000 and 1:4,000 dilution, respectively.

### ADP Glo^TM^ assay

Recombinant components of the KEOPS complex were tested for ATPase activity using the ADP-Glo Kinase assay kit (Promega) as per manufacturer’s instructions. In a 20 µL reaction wild-type or mutant *ar*Bud32 (0.3 µM final) and other components of the *ar*KEOPS complex (0.35 µM final) and tRNA (3 µM final, except for the experiment shown in Supplementary Fig. 8e in which the tRNA concentration was 1 µM) were added where indicated. The buffer contained 40 mM Tris pH 7.5, 50 mM NaCl, 10 mM MgCl_2_, 2 mM MnCl_2_, 0.1 mg/ml BSA, 0.01% Brij 35, and 1 mM DTT. Reactions were initiated by adding ATP (10 µM final) and incubating at 55 °C for 60 min. Reactions were terminated by transferring 10 µL of the reaction mix to a 384 well white plate (Lumitrac 200, VWR) containing 10 µL of ADP-Glo^™^ Reagent and incubating at room temperature for 40 min. 20 µL of kinase detection reagent was then added and allowed to incubate at room temperature for 30 min. Luminescence was measured on a BioTek Synergy Neo plate reader (BioTek) with Gen5 v2.05 software using a 1 s integration time. Results were plotted in GraphPad Prism v8.3 (GraphPad). Experimental procedures using the human homologs were carried out using the identical procedures with the following exceptions. The concentrations of proteins and tRNA were 0.2 µM and 2 µM, respectively. The reactions were initiated with 100 µM ATP and all incubations were performed at room temperature.

### Electron microscopy

1 µM of reconstituted *ar*KEOPS co-purified from a S200 sizing column with or without 90 µM *mj*tRNA^Lys^. Samples were adsorbed to a glow-discharged carbon-coated copper grid and stained with 0.2 mg/mL uranyl acetate (Electron Microscopy Sciences) as previously described in ref. ^60^. Micrographs were recorded using a 4k CCD Orius Gatan camera attached to a FEI Tecani T20 transmission electron microscope operated at an accelerating voltage of 200 kV with a LaB6 filament, at a defocus value of −0.5 µm and magnification of 200kX. Collected images have a calibrated pixel size of 1.935 Å.

### Image processing

Data analysis (from particle picking to density map refinement) was performed using Cryosparc v2.13.0^61^. A total of 13233 particles of *ar*KEOPS with *mj*tRNA^Lys^ and 6148 of *ar*KEOPS without *mj*tRNA^Lys^ were selected for two-dimensional classification using a 200 Å inner mask into 20 classes. Particles belonging to classes of poor quality were discarded, and the remaining good particles were used to calculate an initial three-dimensional (3D) model using the ab initio reconstruction functionality of CryoSparc v2.13.0. The initial model was further refined using the CryoSparc heterogeneous refinement algorithm to generate four 3D classes. The reconstructions presented in the text consisted of 6395 and 2321 particles for *ar*KEOPS with and without bound tRNA, respectively. Molecular docking of structural models into the 3D reconstructions was performed using Pymol v1.7.4.3. For pairwise comparison of the negative stain 3D reconstruction with our proposed model, we calculated a theoretical envelope of 25 Å resolution based on the coordinates of our proposed atomic model using the e2pdb2mrc.py algorithm from the EMAN2 suite v1.9^62^.

### Yeast strains, expression plasmids, and growth assays

See Supplementary Table 6 for a list of yeast strains and Supplementary Table 7 for a list of yeast expression plasmids. For yeast expression plasmids, gene synthesis (Invitrogen) was used to create fusions of the wild-type coding sequence of *CGI121*, *KAE1* and *PCC1* genes with an in-frame FLAG tags (N-terminal 2XFLAG for *sc*Cgi121 and *sc*Kae1, a single C-terminal FLAG tag for *sc*Bud32 and *sc*Pcc1) that were flanked by 500 bp of up- and downstream genomic sequences of the respective genes. The tagged genes were subcloned into plasmid pRS415^63^, except for 2xFLAG-CGI121 which was subcloned into pRS414^63^. For the *sc*Bud32 expression plasmid, ~500 bp of genomic sequence upstream of the *BUD32* coding sequence was PCR-amplified from yeast genomic DNA and used to replace the *GAL1* promoter in pGAL-BUD32-FLAG^64^. Mutant alleles were generated by site-directed mutagenesis using standard protocols and were confirmed by plasmid sequencing. The pRS414-2xFLAG plasmid was created by deleting the CGI121 coding sequence from the pRS414-2xFLAG-CGI121 expression plasmid.

For growth assays, cells were typically grown at 30 °C overnight in 2% glucose-containing synthetic complete media lacking uracil, leucine or tryptophan as appropriate to maintain plasmid selection, and diluted to an OD_600_ of 0.2 the following morning. Cells were grown for 3–4 h, diluted to an OD_600_ of 1.0 in water followed by serial five-fold dilutions in water. Cell dilutions were spotted on the appropriate selective plates and incubated at 30 °C or as indicated in figure legends. For strains containing the *cdc13-1* allele, cells were freshly transformed with plasmids, transformants were precultured at 20 °C in liquid media and plates were incubated at either 20 °C or 26 °C. Where indicated, *URA3*-linked cover plasmids were counter-selected by growth on synthetic complete medium lacking leucine plus 0.8 mg/mL 5-fluoroorotic acid (5-FOA).

### tRNA isolation from yeast cells

Yeast cells were grown in 100 mL liquid cultures in selective synthetic media to an OD_600_ of 1–2. Cells were pelleted and tRNA was extracted using a NucleoBond RNA/DNA 400 (Macherey-Nagel) kit according to the manufacturer’s instructions.

### Immunoprecipitation of FLAG-tagged Cgi121 proteins and RNA extraction from immunoprecipitates for northern blotting

*bud32*Δ yeast cells were grown in 250 mL cultures of selective synthetic media until an OD_600_ of ~1 and harvested by centrifugation. Cell pellets were resuspended in 400 µL lysis buffer (50 mM NaCl, 100 mM Hepes pH 7.5, 55 mM KoAC, 0.5% Triton X, 0.1% Tween-20) supplemented with 0.2 mM PMSF, 1 tablet of protease inhibitor cocktail (Sigma, 05056489001) and 200 U/mL RiboLock (Thermo Scientific, EO0381). 100 µL acid washed glass beads were added to the resuspended cells and the mixture was vortexed for a total of 2 min (20 s vortex followed by 1 min on ice for a total of 6 times). Cell debris were pelleted (10,000 *g*, 2 min at 4 °C) and supernatant was collected. Total protein concentrations were measured using the Bradford method. Samples containing 500 µg of total protein were adjusted to a volume of 500 µL in lysis buffer, then precleared for background RNA binding by adding 25 µL protein G Sepharose beads (Pierce, #22851) followed by rotation at 4 °C for 30 min. Samples were spun down (500 g, 2 min at 4 °C) and supernatants were transferred to a fresh tube. 2 µg of anti-FLAG antibody (abcam, ab1257) was added to each sample followed by rotation at 4 °C for 60 min. 25 µL of protein G Sepharose beads were added to each sample followed by rotation at 4 °C for a further 60 min. The affinity resin was then washed four times with 1 mL lysis buffer. After washing, beads were either resuspend in SDS-PAGE protein loading buffer for immunoblot analysis or in 400 mL lysis buffer for RNA extractions. 400 µL of a 25:25:1 phenol: chloroform:isoamyl alcohol mixture was added to each RNA extraction sample. Samples were then vortexed for 15 s and centrifuged for 2 min for phase separation. The upper aqueous phase was transferred to a new tube and were mixed with 2 µL glycogen, 40 µL 3 M ammonium acetate pH 5.2 and 1100 µL ethanol and then incubated at −80 °C overnight to precipitate RNA. Precipitated RNA was collected by centrifugation (18,000 *g*, 30 min at 4 ˚C) washed once with 70% ethanol air dried, resuspended in water and subjected to northern blot analysis.

### RNA preparation from yeast cultures and positive hybridization (PHA37) northern blotting

Yeast strains were grown, after counterselection of a cover plasmid where applicable, at 30 °C or at 37 °C in synthetic selective medium and harvested at mid-log phase. Total RNA was isolated with hot acid phenol and northern blot analysis was performed as described^36,65^ using separation on 8% TBE-urea polyacrylamide gels. Band intensities were quantified with ImageQuant TL v2005 software. Relative modification index values represent the intensity of the TΨC signal divided by the intensity of the PHA37 signal, normalized to knockout strains expressing wild-type proteins. Probe sequences and annealing temperatures are provided in Supplementary Table 8.

### HDX analysis

A peptide map of the KEOPS complex was determined using data-dependent acquisition on a Sciex 5600 TripleTOF in a recursive manner, to obtain maximal peptide coverage. KEOPS was digested under HDX conditions for this map, for 2 min at 7 °C using nepenthesin II in 100 mM Gly-HCl (pH 2.3). Digests were injected into a chilled reversed-phase nanoLC system^66^ and separated with a short 10-min gradient of acetonitrile. Upon completion of the map, the optimum labeling timepoint for comparative analysis (KEOPS±tRNA) was determined by labeling KEOPS with 45% D_2_O (buffered with 20 mM Hepes pH 7, 100 mM NaCl and 2 mM DTT) and sampling at 60, 180, 300, 600, 3600 s. Samples were quenched and digested for 2 min at 7 °C using nepenthesin II in 100 mM Gly-HCl (pH 2.5), and peptides separated as above. Peptides were identified using HXpipe (using default parameter settings) and deuterium uptake for each peptide measured using HXdeal in Mass Spec Studio (v2) (manuscript in preparation). The 300 s labeling timepoint was chosen for comparative analysis, as the first timepoint with deuterium incorporation approaching 90% of the maximum for most peptides. KEOPS (10 µM) was then incubated with tRNA (10 µM) for 10 min in binding buffer (20 mM Hepes pH 7, 100 mM NaCl and 2 mM DTT), labeled with 45% D2O for 300 s, then quenched and digested as above. Bound KEOPS samples were collected in quadruplicate and interleaved with tRNA-free KEOPS samples and blanks (to prevent sample carryover). Deuterium uptake was measured using HXdeal and significant differences between states determined as previously described^67^. Summary statistics, as per community guidelines^68^, are found in Supplementary Tables 9 and in Supplementary data 1, and Supplementary Fig. 6d. The mass spectrometry proteomics data have been deposited to the ProteomeXchange Consortium via the PRIDE^69^ partner repository with the dataset identifier PXD018007.

### Statistics and reproducibility

All experiments in this study were repeated at least twice with essentially the same results, with the exception of those shown in Fig. 4b (for which ~50 micrographs were collected for each sample), supplementary figure 3a and supplementary figure 8a that were done once.

### Reporting summary

Further information on research design is available in the Nature Research Reporting Summary linked to this article.

## Supplementary information

Supplementary Information

Peer Review File

Reporting Summary

Description of Additional Supplementary Files

Supplementary Data 1

## Source data

Source Data

## Data Availability

Coordinates and structure factors for the structures of *mj*tRNA^Lys^_UUU_ and the *mj*Cgi121*–mj*tRNA^Lys^_UUU_ complex are available at the Protein Data Bank (https://www.rcsb.org/) with accession codes “7KJT“ and “7KJU”, respectively. NMR backbone resonance assignments for *mj*Cgi121 were obtained from the Biological Magnetic Resonance Data Bank with identifier “15981“. The mass spectrometry proteomics data have been deposited to the ProteomeXchange Consortium via the PRIDE partner repository (https://www.ebi.ac.uk/pride/archive/) with the dataset identifier “PXD018007“. tRNA sequences were obtained from GtRNAdb website (http://gtrnadb.ucsc.edu/). All relevant data supporting the findings in this study are provided within this article or its Supplementary files or from the corresponding author upon reasonable request. Source data are provided with this paper.
