# Author Correction: A substrate binding model for the KEOPS tRNA modifying complex

**DOI:** 10.1038/s41467-025-60136-2

**Published:** 2025-05-22

**Authors:** Jonah Beenstock, Samara Mishelle Ona, Jennifer Porat, Stephen Orlicky, Leo C. K. Wan, Derek F. Ceccarelli, Pierre Maisonneuve, Rachel K. Szilard, Zhe Yin, Dheva Setiaputra, Daniel Y. L. Mao, Morgan Khan, Shaunak Raval, David C. Schriemer, Mark A. Bayfield, Daniel Durocher, Frank Sicheri

**Affiliations:** 1 The Lunenfeld-Tanenbaum Research Institute, Mount Sinai Hospital, Toronto, ON Canada; 2 Department of Molecular Genetics, University of Toronto, Toronto, ON Canada; 3 Department of Biology, York University, Toronto, ON Canada; 4 Department of Biochemistry, University of Toronto, Toronto, ON Canada; 5 Department of Chemistry, University of Calgary, Calgary, AB Canada; 6 Department of Biochemistry and Molecular Biology, University of Calgary, Calgary, AB Canada

**Keywords:** Chemical modification, RNA modification, tRNAs, X-ray crystallography

Correction to: *Nature Communications* 10.1038/s41467-020-19990-5, published online 4 December 2020

In the version of the article initially published, there was a model building error in the reported crystal structure of an isolated tRNA molecule (PDB 7KJU) and the X-ray crystal structure of the same tRNA molecule bound to CGI121 (PDB 7KJT). The error consists of a register shift of one position for the first 16 nucleotides in the tRNA sequence. The two X-ray crystal structures have been corrected, and the data collection and refinement statistics listed in Supplementary Table 1 has been amended in the HTML version of the article. The error in tRNA structure did not materially affect any of the descriptions or conclusions drawn in the article.

## Supplementary information

Updated Supplementary Information

