# Supplementary material for: A substrate binding model for the KEOPS tRNA modifying complex
Source: Nat Commun. 2020 Dec 4;11:6233. doi: 10.1038/s41467-020-19990-5 (PMC7718258; doi:10.1038/s41467-020-19990-5)
Supplement: Supplementary file 1 — Supplementary Information [file 41467_2020_19990_MOESM1_ESM.pdf]

Supplementary information

A substrate binding model for the KEOPS tRNA modifying complex

Jonah Beenstock [1], Samara Mishelle Ona [1, 2], Jennifer Porat [3], Stephen Orlicky [1], Leo C.K. Wan [1,2], Derek F. Ceccarelli [1], Pierre Maisonneuve [1], Rachel K. Szilard [1], Zhe Yin [1, 4], Dheva Setiaputra [1], Daniel Y.L. Mao [1], Morgan Khan [5], Shaunak Raval [5], David C. Schriemer [5, 6], Mark A. Bayfield [3], Daniel Durocher [1, 2], and Frank Sicheri [1, 2, 4] *

Affiliations

[1] The Lunenfeld-Tanenbaum Research Institute, Mount Sinai Hospital, Toronto, Ontario, Canada, [2] Department of Molecular Genetics, University of Toronto, Toronto, Ontario, Canada, [3] Department of Biology, York University, Toronto, Ontario, Canada, [4] Department of Biochemistry, University of Toronto, Toronto, Ontario, Canada, [5] Department of Chemistry, University of Calgary, Calgary, Alberta, Canada, [6] Department of Biochemistry and Molecular Biology, University of Calgary, Alberta, Canada.

* Correspondence: sicheri@lunenfeld.ca

Supplementary Figure 1 (associated with Figure 1)

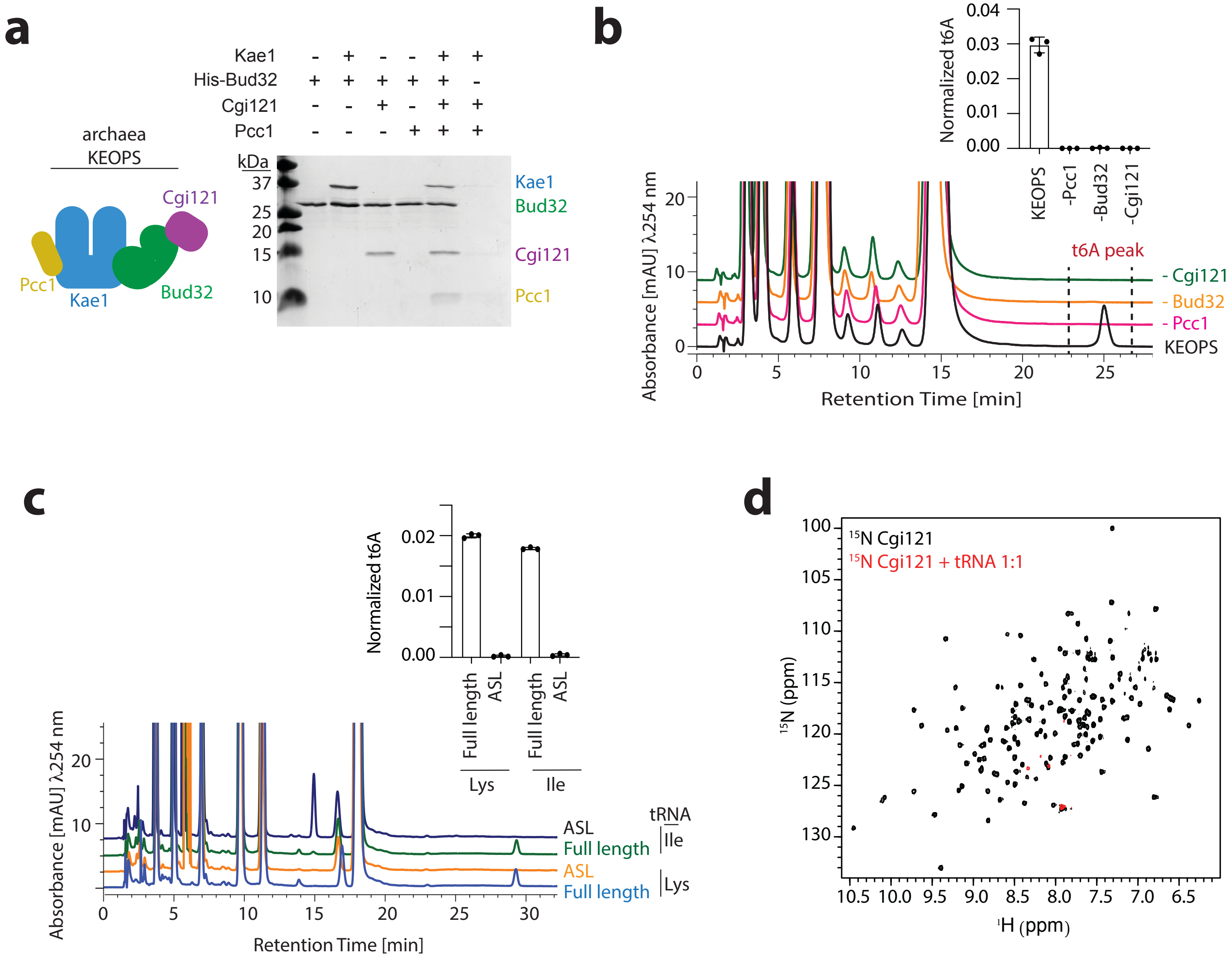

Supplementary Figure 1. *mj*Cgi121 is a tRNA binding protein.

a Schematic representation of the linear binding architecture of the KEOPS complex (left). The linear binding architecture of *ar*KEOPS can be recapitulated with purified proteins *in vitro*. His-*mj*Bud32 was used as bait followed by pull-down with nickel chelate resin and analyzed by SDS-PAGE and Coomassie staining (right)

b *In vitro* t^6A modification activity analysis of the indicated reconstituted *ar*KEOPS sub-complexes. Shown are representative HPLC profiles of nucleoside composition for each reaction (lower panels) together with quantification (upper panels) of average t^6A content normalized to the content of uridine (n=3 independent experiment samples, ±SD).

c *In vitro* t^6A modification activity analysis of reconstituted *ar*KEOPS towards full length tRNA and a fragment of the anticodon stem loop (ASL) of *mj*$tRNA^{Lys}$ and *sc*$tRNA^{Ile}$. Shown are representative HPLC profiles of nucleoside composition for each reaction together with quantification of average t^6A content normalized to the content of uridine (n=3 independent experiment samples, ±SD).

d Nuclear magnetic resonance spectroscopic ^1H-^{15}N-HSQC analysis of ^{15}N-labeled *mj*Cgi121 in the absence (black) or presence (red) of a 1:1 molar ratio of *mj*$tRNA^{Lys}$.

Supplementary Figure 2 (associated with Figure 2)

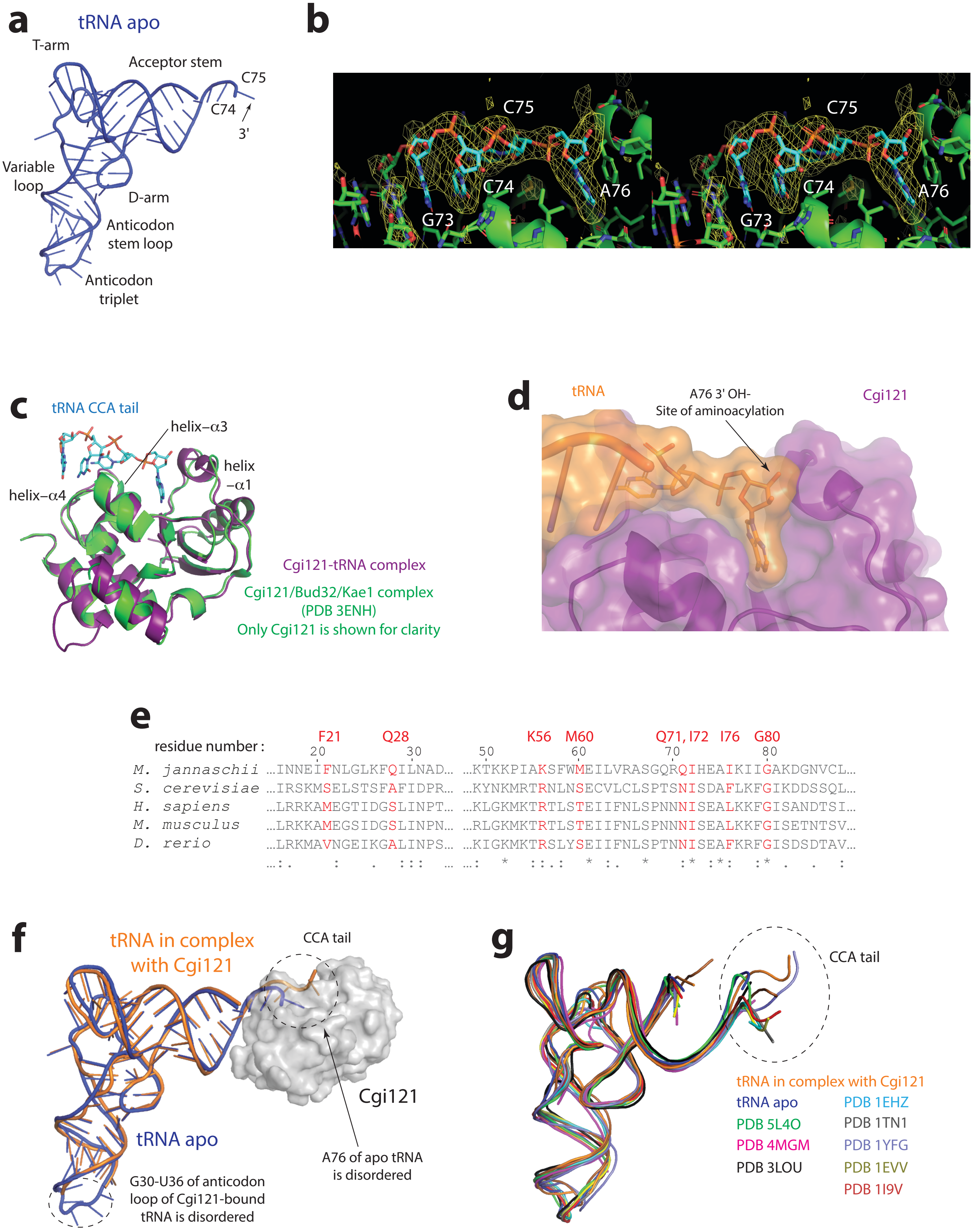

Supplementary Figure 2. Crystal structure of apo-*mj*tRNA$^{Lys}_{UUU}$ and comparison to previously determined tRNA and Cgi121 structures.

a The crystal structure of apo-*mj*tRNA$^{Lys}_{UUU}$ shown in cartoon representation, highlighting conserved elements.

b Stereo view of the binding interface between *mj*Cgi121 and *mj*tRNA$^{Lys}_{UUU}$ (similar to the orientation in **Fig. 2c**) showing un-biased Fo-Fc electron density of the tRNA CCA tail contoured at 2.0 σ. Individual chains of Cgi121 and tRNA (Δ72-76) modified from the final structure were used as molecular replacement search models using Phaser. The solution was refined in Phenix for three rounds with a simulated annealing temperature gradient from 5000 to 300K in 100K steps. The output model (Rfactor: 0.283 and Rfree: 0.316) was of lower statistical quality than the complete structure (Rfactor: 0.223, Rfree: 0.255). Fo-Fc map was generated by FFT (Fast Fourier Transform, CCP4 program) and displayed using Pymol.

c Superimposition of the structures of *mj*Cgi121 bound to *mj*tRNA$^{Lys}_{UUU}$ (purple) and that of *mj*Cgi121 bound to *mj*Bud32-*mj*Kae1 (PDB 3ENH, green). For clarity, only the CCA tail of *mj*tRNA$^{Lys}_{UUU}$ is shown (cyan), and *mj*Bud32 and *mj*Kae1 are omitted.

d Surface representation of the *mj*Cgi121-*mj*tRNA$^{Lys}_{UUU}$ binding interface highlighting the predicted site of steric clash arising from aminoacylation of tRNA.

e Sequence alignment of Cgi121 orthologs highlighting the conservation of the residues that participate in tRNA binding as shown in **Fig. 2b**.

f Superimposition of the structures of apo-*mj*tRNA$^{Lys}_{UUU}$ (blue) and *mj*tRNA$^{Lys}_{UUU}$ bound to Cgi121 (orange and grey, respectively). Differences in the conformations of the CCA tail and anticodon loop are highlighted.

g Superimposition of previously reported apo-tRNA crystal structures with the crystal structures of apo- and *mj*Cgi121-bound *mj*tRNA$^{Lys}_{UUU}$ structures reported here, highlighting the variability in conformation of the CCA tail. Cgi121 was omitted for clarity.

Supplementary Figure 3 (associated with Figure 3)

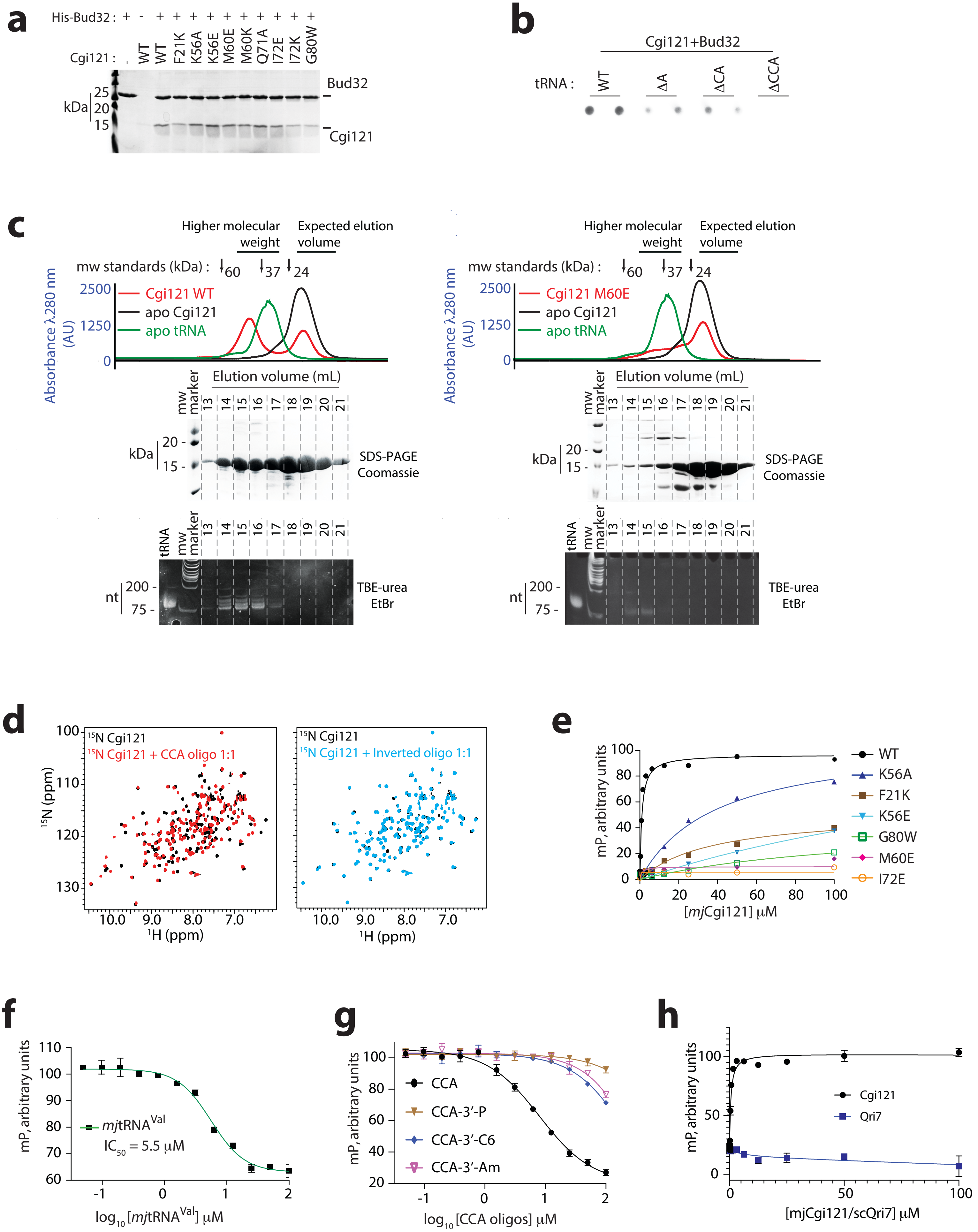

Supplementary Figure 3. Probing the tRNA binding activity of *mj*Cgi121.

a Binding analysis of wild-type *mj*Cgi121 or the indicated mutants to His-*mj*Bud32. His-*mj*Bud32 bait was pulled down using nickel chelate resin and interacting proteins were resolved by SDS-PAGE and visualized by Coomassie staining.

b Analysis of binding of the *mj*Cgi121-*mj*Bud32 complex to wild-type or indicated mutants of *mj*tRNA^{Lys} using a filter binding assay.

c Wild-type *mj*Cgi121, but not a Met60Glu mutant, co-purifies with endogenous tRNA when expressed in *E. coli*. (Top) Size exclusion chromatography profiles of *mj*Cgi121 proteins expressed and purified from bacteria. Wild-type protein purification is shown on the left and that of the Met60Glu mutant is shown on the right. Molecular weight (mw) marker positions highlighted by arrows correspond to *mj*Bud32, *mj*Kae1 and the *mj*Kae1-*mj*Bud32 fusion protein (24, 37 and 60 kDa respectively). The chromatograms of apo-tRNA and apo-Cgi121 are shown for comparison. (Middle) SDS-PAGE analysis of sizing column fractions. Proteins were visualized by Coomassie staining. (Bottom) Denaturing electrophoresis (TBE-urea PAGE) of nucleic acids purified from phenol-chloroform extractions of sizing column fractions and visualized by ethidium bromide (EtBr) staining.

d Nuclear magnetic resonance spectroscopic ^{1}H-^{15}N-HSQC analysis of ^{15}N-labeled *mj*Cgi121 in the presence or absence of a 1:1 molar ratio of the 5'-CCCGCCA-3' (CCA oligo; left panel) or 5'-ACCGCCC-3' (control inverted CCA oligo; right panel) oligonucleotides. Strong perturbation of resonance peaks consistent with binding to *mj*Cgi121 was observed for the CCA oligo but not the control inverted oligo.

e Binding analysis of wild-type *mj*Cgi121 or the indicated mutants to the 647-CCA probe measured by fluorescence polarization (n=2 independent experiment samples).

f Competitive displacement of the 647-CCA probe bound to *mj*Cgi121 by the KEOPS non-substrate *mj*tRNA^{Val}. Displacement of the 647-CCA probe was monitored by fluorescence polarization (n=3 independent experiment samples, ±SD).

g Competitive displacement of the 647-CCA probe bound to *mj*Cgi121 by CCA-oligos harboring 3' modifications comprised of phosphate (P), a six-carbon linear aliphatic chain (C6), or a 9-atom amino group (Am). See **Supplementary Table 2** for chemical structures. Displacement of the 647-CCA probe was monitored by fluorescence polarization (n=3 independent experiment samples, ±SD).

h Binding analysis reveals that *sc*Qri7, unlike *mj*Cgi121, does not bind to the 647-CCA probe as measured by fluorescence polarization (n=3 independent experiment samples, ±SD).

Supplementary Figure 4 (associated with Figure 3)

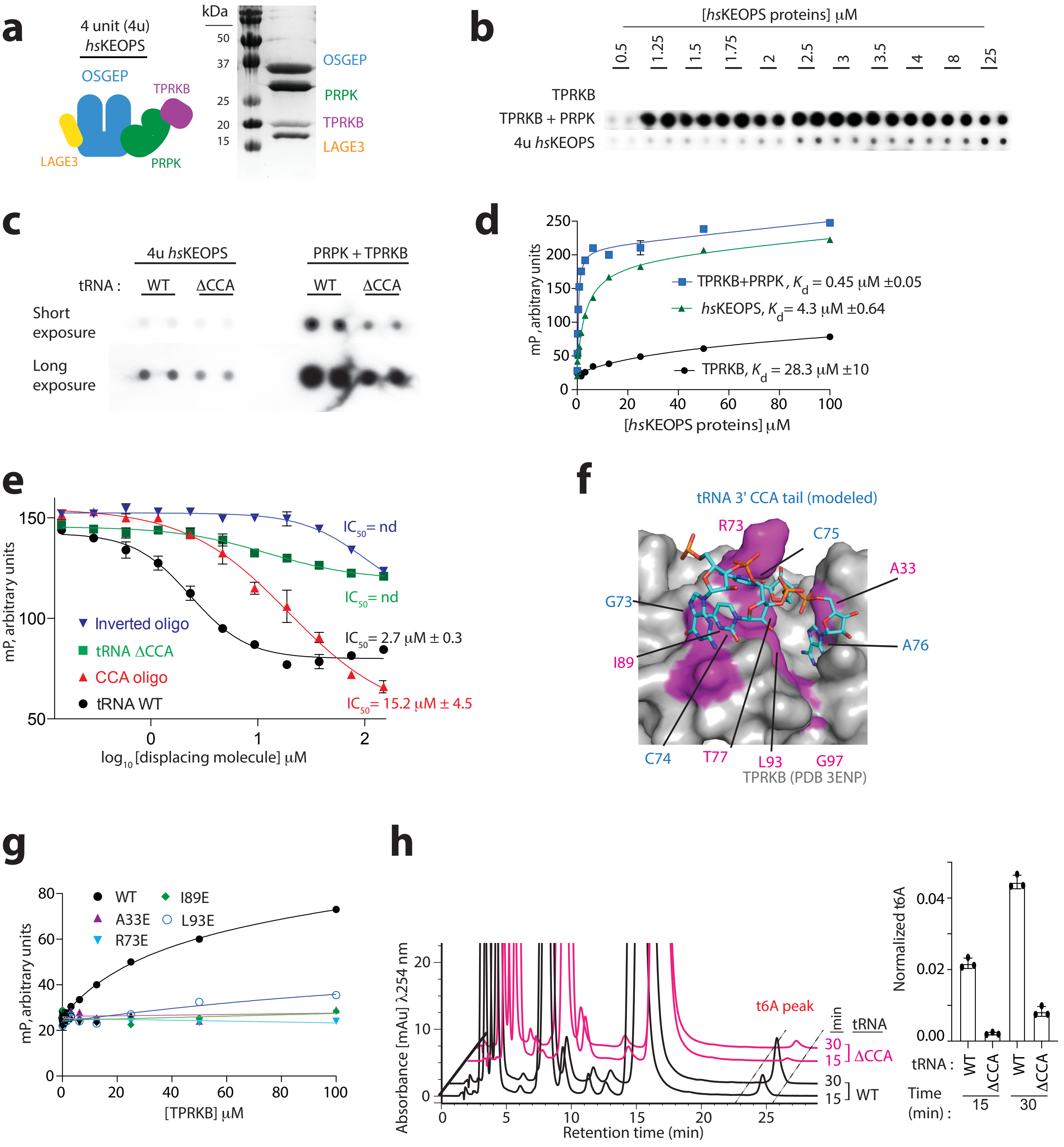

Supplementary Figure 4. The function of human KEOPS depends on CCA tail binding by TPRKB.

a (Left) Schematic representation of the four-subunit human KEOPS (4u *hs*KEOPS) core complex. (Right) SDS-PAGE analyses of co-purified TPRKB, PRPK, OSGEP and LAGE3 visualized by Coomassie staining.

b Binding analysis of the indicated human KEOPS proteins to wild-type *mj*tRNA^{Lys} using a filter binding assay.

c Binding analysis of the indicated human KEOPS proteins to *mj*tRNA^{Lys} or *mj*tRNA$^{Lys, \Delta CCA}$ using a filter binding assay. Concentrations of *hs*KEOPS and the TPRKB+PRPK complex were 3 μM and 0.8 μM respectively.

d Binding analysis of the indicated human KEOPS components towards the 647-CCA probe measured by fluorescence polarization. Representative profiles and average K_d values are shown (n=3 independent experiment samples, ±SD).

e Competitive displacement of a 647-CCA probe from human KEOPS (4 μM) with increasing concentrations of unlabeled wild-type *mj*tRNA^{Lys}, *mj*tRNA$^{Lys, \Delta CCA}$, CCA oligo (5'-CCCGCCA-3') or inverted CCA oligo (5'-ACCGCCC-3'). Displacement of the 647-CCA probe was monitored by fluorescence polarization (n=3 independent experiment samples, ±SD).

f Superimposition of *mj*tRNA^{Lys} (blue) bound to *mj*Cgi121 onto the structure of the human Cgi121 ortholog TPRKB (PDB 3ENP, grey). Only the CCA tail of the *mj*tRNA^{Lys}-*mj*Cgi121 structure is shown for clarity. tRNA-contacting residues on the surface of TPRKB are highlighted (magenta).

g Binding analysis of wild-type TPRKB or the indicated mutants to the 647-CCA probe measured by fluorescence polarization (n=2 independent experiment samples).

h *In vitro* t^6A modification activity analysis of *hs*KEOPS towards wild-type *mj*tRNA^{Lys} or *mj*tRNA$^{Lys, \Delta CCA}$. Reactions were stopped at the indicated time points. Shown are representative HPLC profiles of nucleoside composition (left) and a quantification (right) of average t^6A content normalized to uridine (n=3 independent experiment samples, ±SD).

Supplementary Figure 5 (associated with Figure 5)

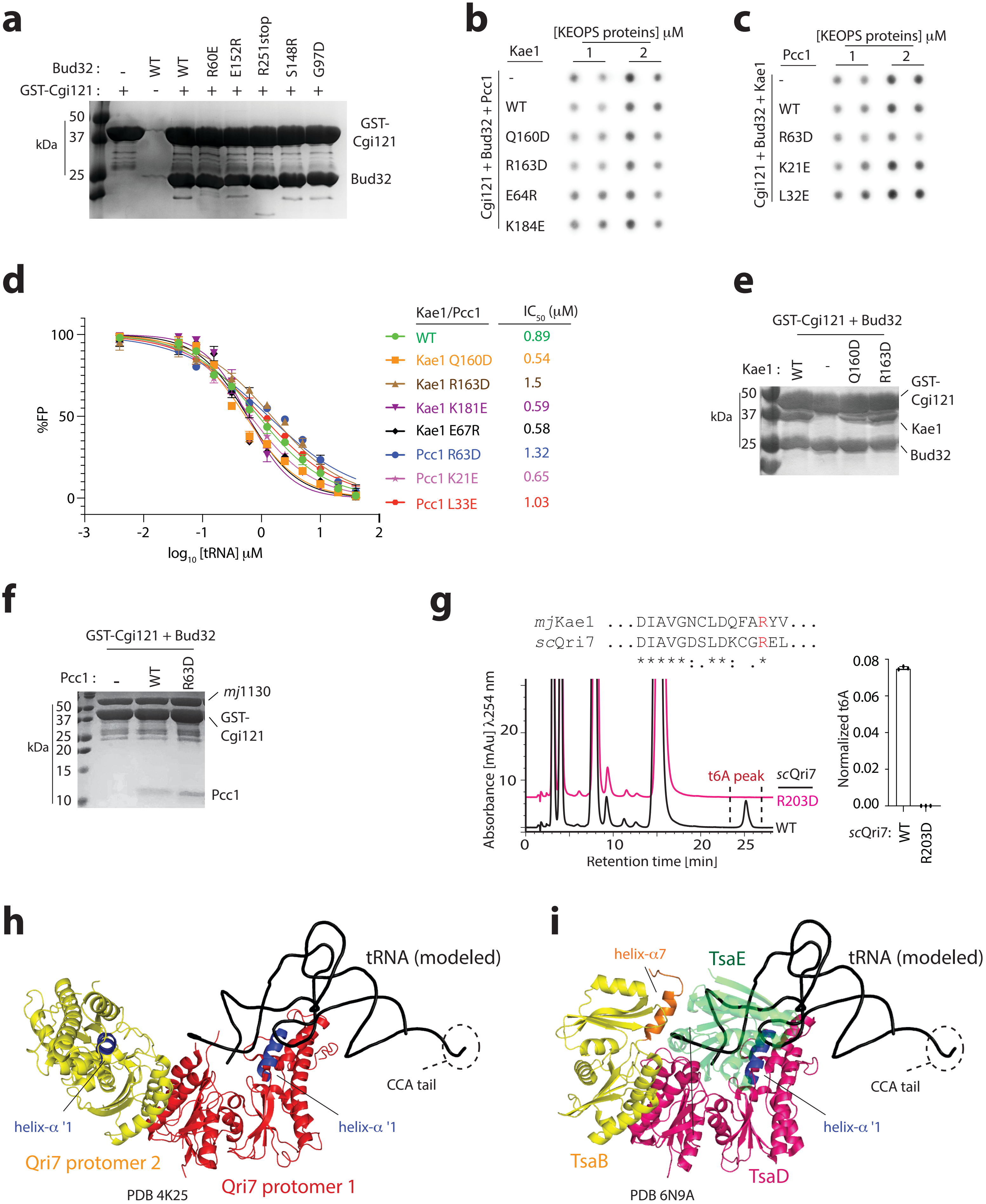

Supplementary Figure 5. Mutational analysis of the tRNA binding surface of KEOPS.

a Interaction analysis between GST-*mj*Cgi121 and wild-type *mj*Bud32 or the indicated mutants assessed by GST pull-down, SDS-PAGE and Coomassie staining.

b-c Analysis of *mj*tRNA^{Lys} binding to KEOPS reconstituted with wild-type or the indicated mutant **b** *mj*Kae1 and **c** *pf*Pcc1 subunits using a filter binding assay.

d Competitive displacement of a 647-CCA probe with increasing concentrations unlabeled *mj*tRNA^{Lys} from the indicated *ar*KEOPS complexes (at 0.75 μM) reconstituted with wild-type or the indicated mutant *mj*Kae1 and *pf*Pcc1 proteins. Displacement of the 647-CCA probe was monitored by fluorescence polarization (n=3 independent experiment samples, ±SD).

e Interaction analysis between GST-*mj*Cgi121-*mj*Bud32 complex and wild-type *mj*Kae1 or the indicated mutants assessed by GST pull-down, SDS-PAGE and Coomassie staining.

f Interaction analysis between GST-*mj*Cgi121-*mj*1130 (a natural fusion of Bud32-Kae1) complex with wild-type *pf*Pcc1 or the indicated mutants assessed by GST pull-down, SDS-PAGE and Coomassie staining.

g *In vitro* t^6A modification activity analysis of wild-type *sc*Qri7 or the Arg203Asp mutant towards *mj*tRNA^{Lys}. Shown are representative HPLC profiles of nucleoside composition for each reaction (left) and quantification (right) of the average t^6A content normalized to uridine (n=3 independent experiment samples, ±SD).

h-i Models of tRNA binding to (**h**) the *S. cerevisiae* Qri7 homodimer or (**i**) the *T. maritima* TsaD-TsaB-TsaE heterotrimer. Models were obtained by superimposition of *mj*Kae1 within the KEOPS-tRNA composite model (**Fig. 4a**) with one Qri7 protomer in a Qri7 dimer (PDB 4K25) or the TsaD subunit in the TsaD-TsaB-TsaE heterotrimer (PDB 6N9A). For clarity, only the tRNA is shown from the KEOPS-tRNA composite model.

Supplementary Figure 6 (associated with Figure 5)

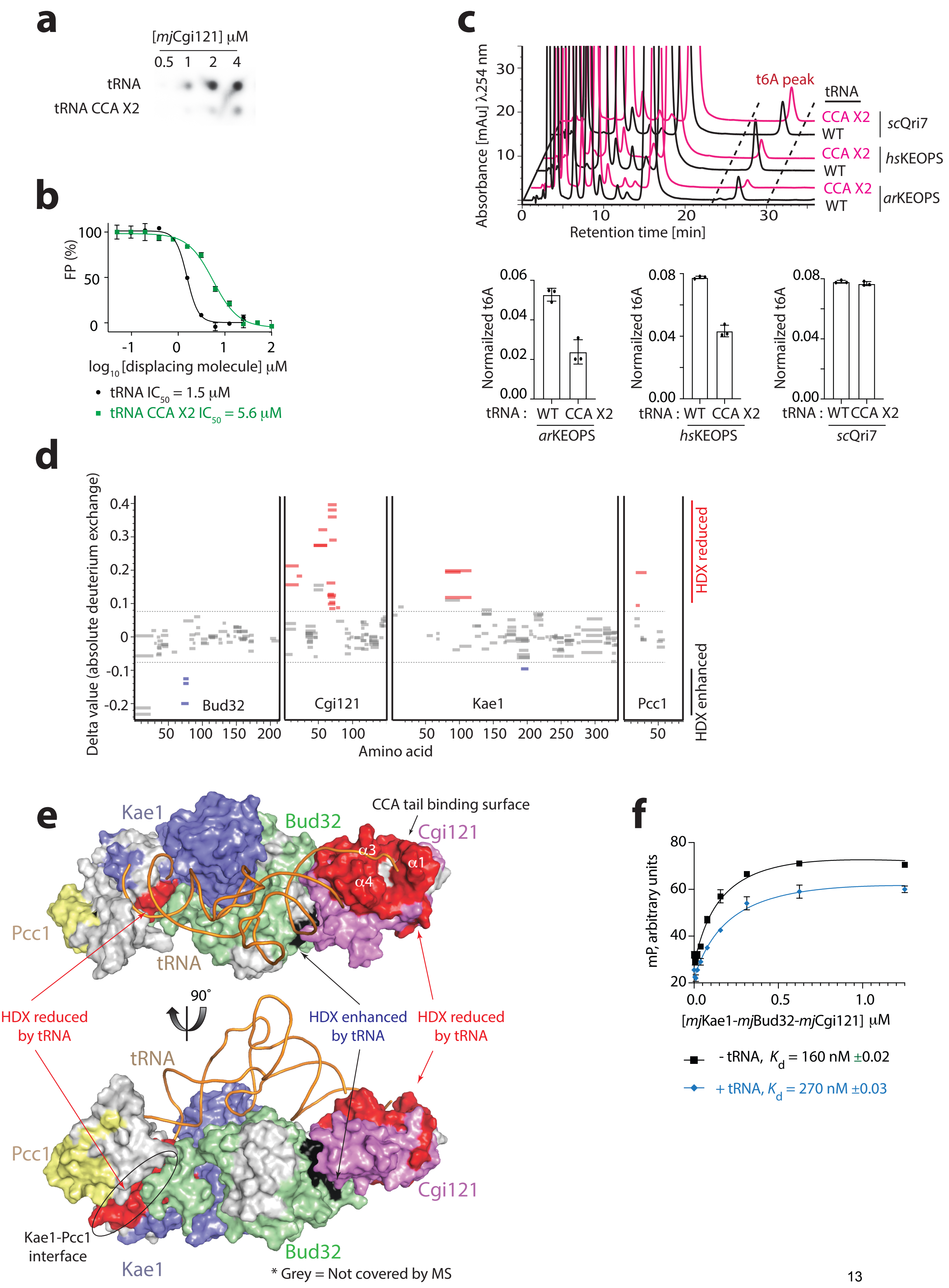

Supplementary Figure 6. Functional characterization of the tRNA binding surface of KEOPS.

a Analysis of *mj*Cgi121 binding to wild-type *mj*tRNA^{Lys} or *mj*tRNA$^{Lys, CCAx2}$ using a filter binding assay.

b Competitive displacement of a 647-CCA probe from *mj*Cgi121 (1.5 μM) by unlabeled wild-type *mj*tRNA^{Lys} or or *mj*tRNA$^{Lys, CCAx2}$. Displacement of the 647-CCA probe was monitored by fluorescence polarization (n=3 independent experiment samples, ±SD).

c *In vitro* t^{6}A modification activity analysis of *ar*KEOPS, *hs*KEOPS and *sc*Qri7 towards wild-type *mj*tRNA^{Lys} or *mj*tRNA$^{Lys, CCAx2}$. Shown are representative HPLC profiles of nucleoside composition for each reaction (upper panels) and quantification (lower panels) of the average t^{6}A content normalized to uridine (n=3 independent experiment samples, ±SD).

d-e HDX analysis of a reconstituted *ar*KEOPS complex in the presence and absence of *mj*tRNA^{Lys}. **d** Woods plot showing the Δvalue of the absolute deuterium exchange rate for each individual peptide in the HDX analysis. **e** Peptides with significantly reduced (red) or increased (blue) HDX in the presence of tRNA are highlighted accordingly on the molecular surface of KEOPS. Likewise, peptides that were not detected by this analysis are shown in black.

f Binding analysis of fluorescently labeled *pf*Pcc1 towards *mj*Kae1-*mj*Bud32-*mj*Cgi121 with or without *mj*tRNA^{Lys} measured by fluorescence polarization (n=3 independent experiment samples, ±SD).

Supplementary Figure 7 (associated with Figure 6)

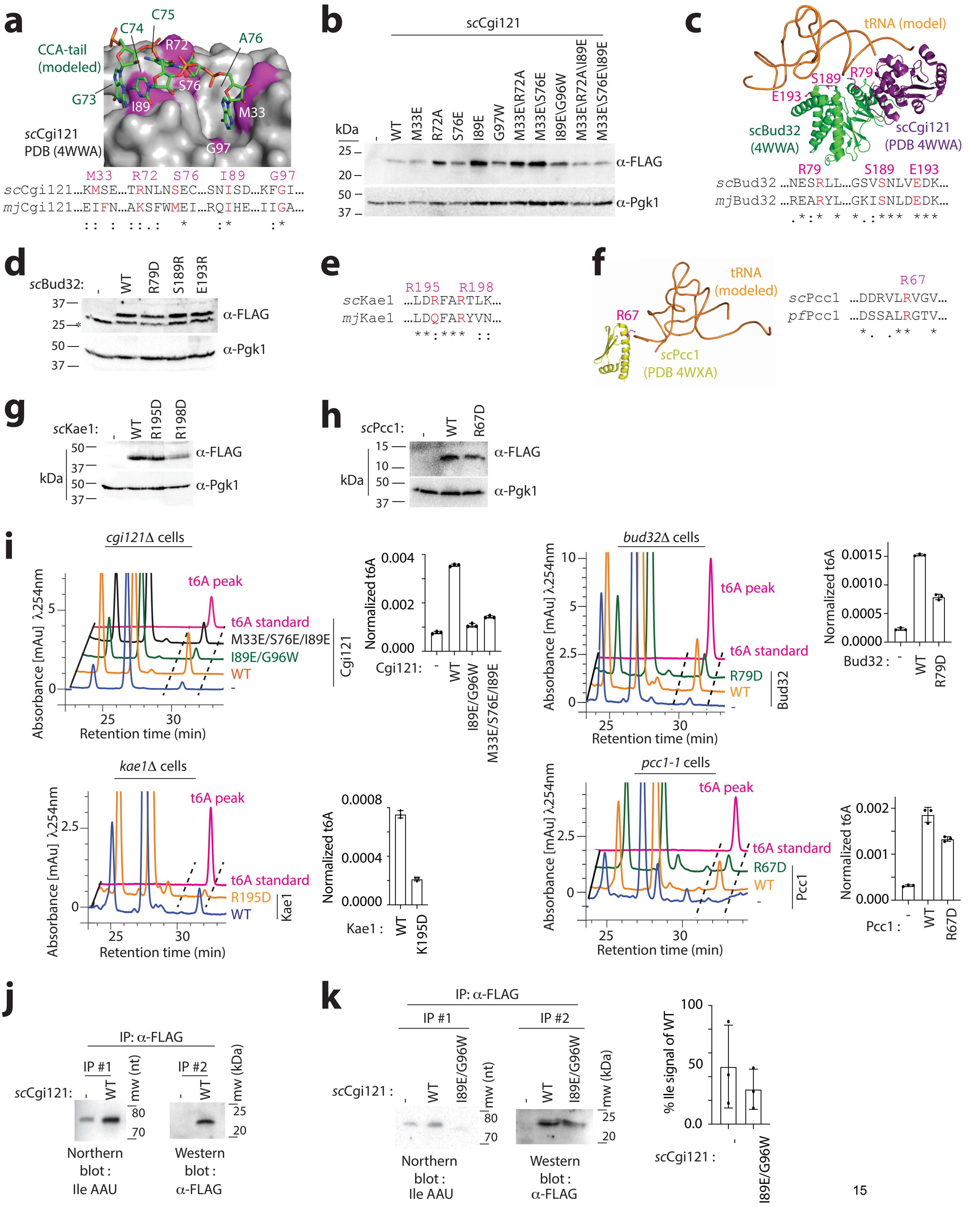

Supplementary Figure 7. Functional characterization of the predicted tRNA binding surfaces of yeast KEOPS.

a (Top) Superimposition of *mj*tRNA^{Lys} (green) bound to *mj*Cgi121 onto the structure of *sc*Cgi121 (PDB 4WWA). tRNA-contacting residues on the surface of *sc*Cgi121 are highlighted in red. Only the CCA tail of *mj*tRNA^{Lys} is shown for clarity. (Bottom) Sequence alignment of *mj*Cgi121 and *sc*Cgi121 over the region implicated in tRNA binding. Residues predicted to directly contact the CCA tail are shown in red.

b Immunoblot analysis of wild-type and mutant FLAG-tagged *sc*Cgi121 proteins expressed in a *cdc13-1, cgi121*Δ yeast strain. Expression levels of Pgk1 were probed as a loading control.

c (Top) Superimposition of *mj*tRNA^{Lys} (orange) from the *ar*KEOPS-tRNA composite model onto the structure of the *sc*Cgi121-*sc*Bud32 complex (PDB 4WWA). tRNA-contacting residues on the surface of *sc*Bud32 are shown in stick representation. For clarity, only tRNA is shown from the KEOPS-tRNA composite model. (Bottom) Sequence alignment of *mj*Bud32 and *sc*Bud32 over the regions implicated in tRNA binding. Residues predicted to directly contact the tRNA are shown in red.

d Immunoblot analysis of wild-type and mutant FLAG-tagged *sc*Bud32 proteins expressed in a *bud32*Δ yeast strain. Expression levels of Pgk1 were probed as a loading control.

e Sequence alignment of *mj*Kae1 and *sc*Kae1 over the regions implicated in tRNA binding. Residues predicted to directly contact the tRNA are shown in red.

f (Left) Superimposition of *mj*tRNA^{Lys} from the KEOPS-tRNA composite model (orange) onto the structure of *sc*Pcc1 (PDB 4WWA). tRNA-contacting residues on the surface of *sc*Pcc1 are shown in stick representation. For clarity, only tRNA is shown from the KEOPS-tRNA composite model. (Right) Sequence alignment of *pf*Pcc1 and *sc*Pcc1 over the regions implicated in tRNA binding.

g Immunoblot analysis of wild-type and mutant FLAG-tagged *sc*Kae1 proteins expressed in a *kae1*Δ yeast strain. Expression levels of Pgk1 were probed as a loading control.

h Immunoblot analysis of wild-type and mutant FLAG-tagged *sc*Pcc1 proteins expressed in a *pcc1-4* yeast strain. Expression levels of Pgk1 were probed as a loading control.

i Analysis of the total cellular t^6A composition in tRNA purified from yeast cells. Bulk tRNAs purified from the indicated yeast strains expressing the wild-type or the indicated mutant KEOPS proteins was digested to nucleosides and analyzed by HPLC. For each strain are shown representative HPLC profiles of nucleoside composition for each reaction (left) and quantification (right) of the average t^6A content was normalized to uridine (n=3 samples from technical replicates, ±SD). *pcc1-4* cells were grown at the non-permissive temperature of 34°C.

j-k Analysis of binding to *sc*$tRNA^{Ile}_{AAU}$ by the indicated FLAG-*sc*Cgi121 proteins expressed in *bud32*Δ yeast cells using co-immunoprecipitation (IP). Binding of *sc*$tRNA^{Ile}_{AAU}$ to immunoprecipitated proteins was analyzed by northern blot and protein content was analyzed by immunoblot. **j** Immunoprecipitates from cells expressing an empty plasmid (-) or FLAG-*sc*$Cgi121^{WT}$. **k** (Left) Immunoprecipitates from cells expressing an empty plasmid (-), FLAG-*sc*$Cgi121^{WT}$ or FLAG-*sc*$Cgi121^{I89E/G96W}$. (Right) The mean of the ratio of quantified band intensities for immunoprecipitate samples from cells expressing an empty plasmid or FLAG-*sc*$Cgi121^{I89E/G96W}$ mutant relative to cells expressing FLAG-*sc*$Cgi121^{WT}$ (n=3 experiments with biologically independent samples, ±SD). mw- molecular weight, nt-nucleotide.

Supplementary Figure 8 (associated with Figure 7)

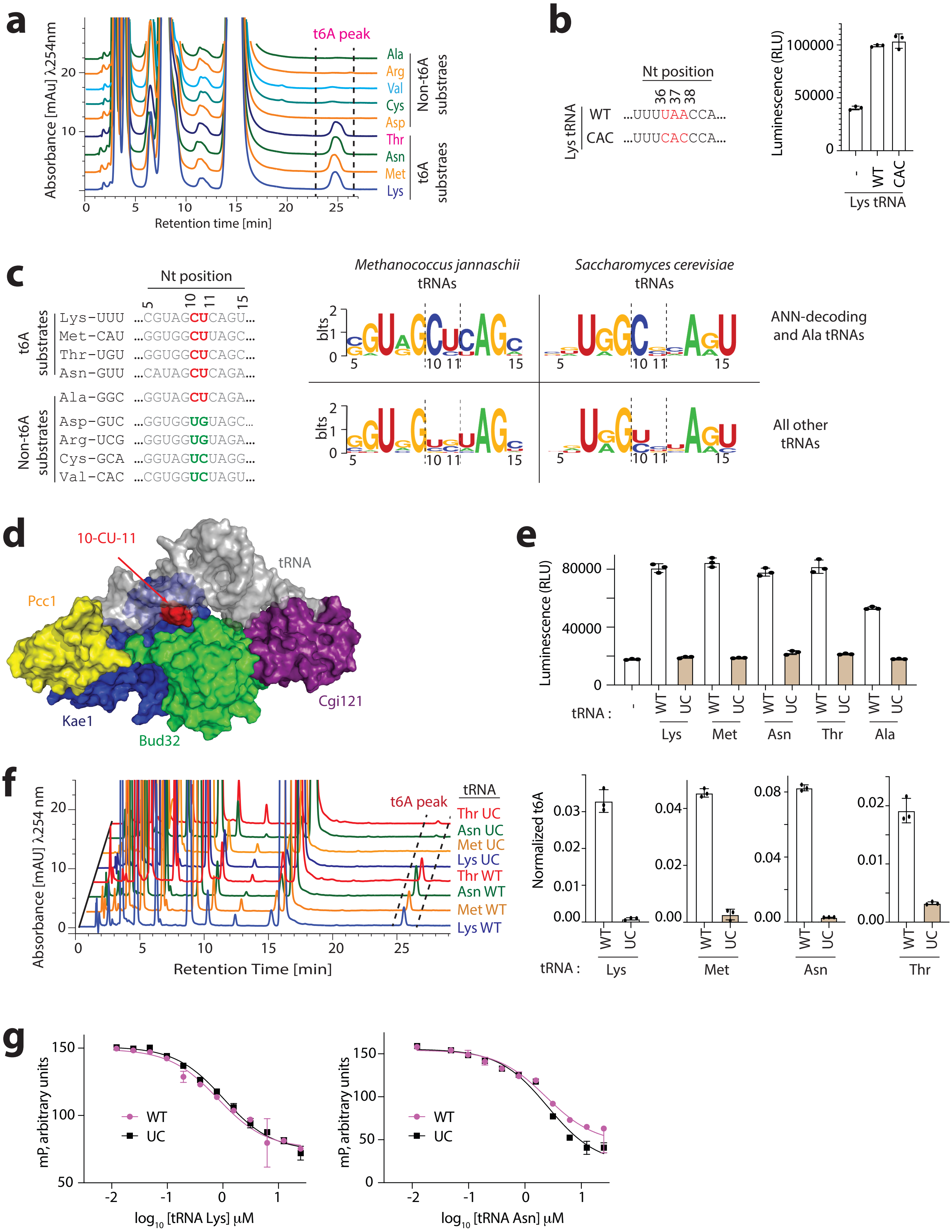

Supplementary Figure 8. tRNA activates the ATPase activity of Bud32.

a *In vitro* t^6A modification activity analysis of *ar*KEOPS towards *mj*$tRNA^{Lys}_{UUU}$, *mj*$tRNA^{Met}_{CAU}$, *mj*$tRNA^{Asn}_{GUU}$, *mj*$tRNA^{Thr}_{GGU}$, *mj*$tRNA^{Asp}_{GUC}$, *mj*$tRNA^{Arg}_{GCG}$, *mj*$tRNA^{Val}_{CAC}$ and *mj*$tRNA^{Ala}_{GGC}$. Shown are representative HPLC profiles of nucleoside composition for each reaction.

b Mutation of the 36-UAA-38 motif of *mj*$tRNA^{Lys}$ does not disrupt ATPase activation. (Left) sequence alignment of wild-type *mj*$tRNA^{Lys}$ and the *mj*$tRNA^{Lys\ CAC}$ mutant. Nt- nucleotide. (Right) ATPase activity analysis of the *ar*KEOPS complex in the presence and absence of wild-type *mj*$tRNA^{Lys}_{UUU}$ or the CAC mutant. Activity was monitored using the ADP Glo assay. Displayed results represent the average luminescence (n=3 independent experiment samples, ±SD)

for each reaction condition.

c The 10-CU-11 motif is characteristic of ANN-decoding and Ala tRNAs in *M. jannaschii*. (Left) Sequence alignments of the indicated *mj*tRNAs analyzed in **Fig. 7c**. Highlighted in red is the CU motif at positions 10 and 11. Nt- nucleotide. (Right) Logo sequences generated from all 9 ANN-decoding and 3 Ala tRNA sequences (top), and all 23 other tRNA sequences obtained from the GtRNAdb (http://gtrnadb.ucsc.edu/).

d The predicted position of the 10-CU-11 motif (highlighted in red) in *mj*$tRNA^{Lys}$ lies adjacent to Bud32 in the KEOPS-tRNA holo-enzyme substrate model.

e-f Mutation of the 10-CU-11 motif in *mj*$tRNA^{Lys}$ disrupts ATPase activation and t6A tRNA modification. **e** ATPase activity analysis of the *ar*KEOPS complex in the presence and absence of the wild-type tRNA or the corresponding indicated 10-UC-11 mutants. Activity was monitored using the ADP Glo assay. Displayed results represent the average luminescence (n=3, ±SD) for each reaction condition. **f** *In vitro* t^6A modification activity analysis of *ar*KEOPS towards the wild-type or the corresponding indicated 10-UC-11 mutant tRNAs. Shown are representative HPLC profiles of nucleoside composition for each reaction (left) and quantifications (right) of the average t^6A content normalized to uridine (n=3 independent experiment samples, ±SD).

g Competitive displacement of a 647-CCA probe from *ar*KEOPS (0.75 μM) by unlabeled wild-type or the 10-UC-11 mutants of *mj*$tRNA^{Lys}$ (left) or *mj*$tRNA^{Asn}$ (right). Displacement

of the 647-CCA probe was monitored by fluorescence polarization (n=3 independent experiment samples, ±SD).

Supplementary Figure 9 (associated with Figure 7)

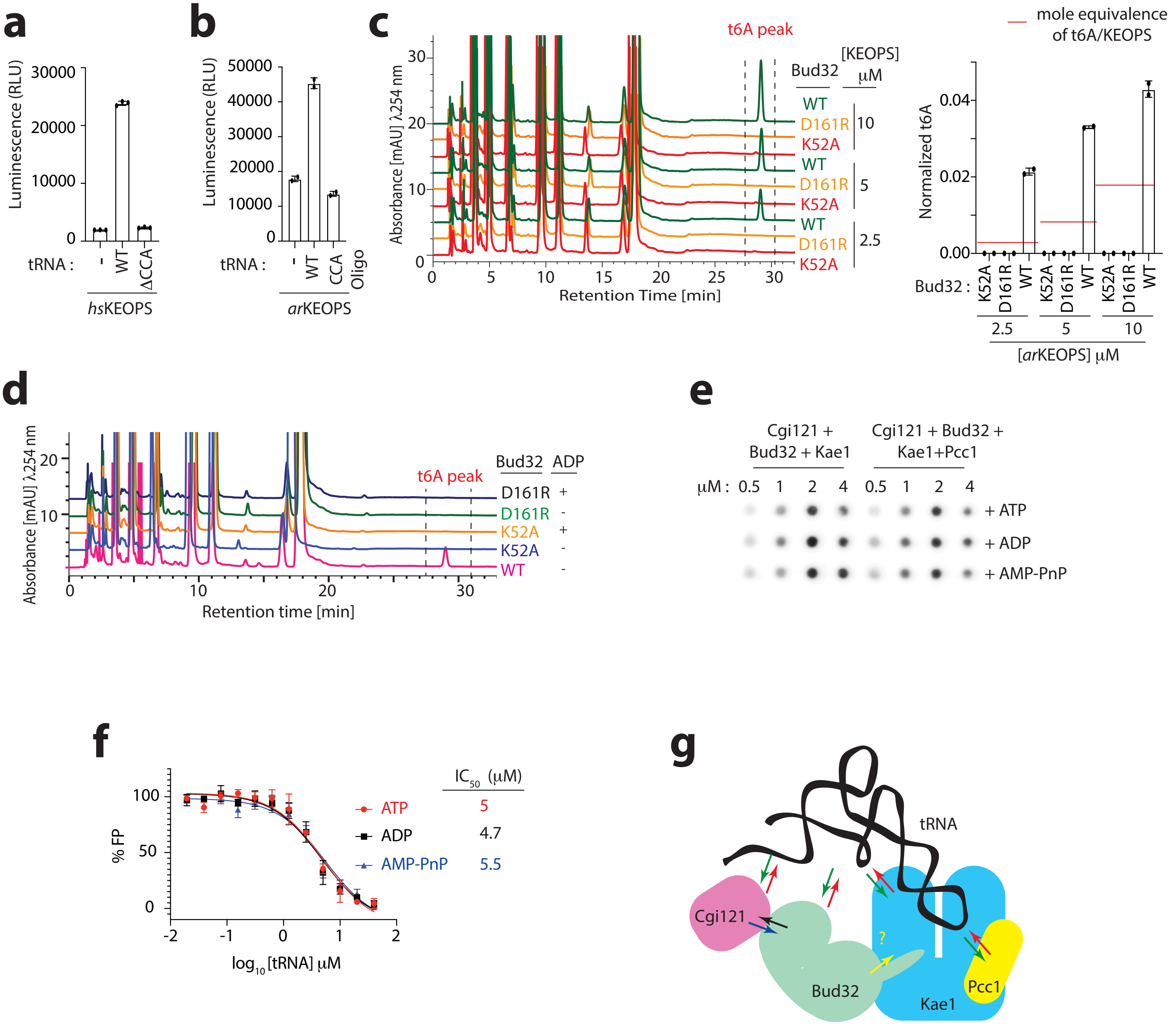

1a. Black arrow.
Bud32 binding to Cgi121 boosts the afinity of Cgi121 for the CCA tail (3-60 fold)

1b. Blue arrow.
Cgi121 binding to Bud32 boosts the ATPase activity of Bud32 (10 fold)

2. Green arrows.
tRNA binding to the Cgi121-Bud32-Kae1-Pcc1 complex boosts the ATPase activity of Bud32 (5-12 fold)

3. Red arrows.
Mutations on the tRNA contact surface of KEOPS subunits prevents the activation of Bud32 by tRNA

4. Yellow arrow.
Bud32 ATPase activates Kae1 t6A modification activity

Supplementary Figure 9. Bud32 ATPase activity is essential for KEOPS tRNA modification activity and does not influence tRNA substrate release.

a ATPase activity analysis of the *hs*KEOPS complex in the presence or absence of wild-type *mj*tRNA$^{Lys}_{UUU}$ or *mj*tRNA$^{Lys}_{UUU}$$^{\Delta CCA}$. Activity was monitored using the ADP Glo assay. Displayed results represent the average luminescence (n=3 independent experiment samples, ±SD) for each reaction condition.

b ATPase activity analysis of the *ar*KEOPS complex in the presence or absence of wild-type *mj*tRNA$^{Lys}_{UUU}$ or the CCA oligo. Activity was monitored using the ADP Glo assay. Displayed results represent the average luminescence (n=3 independent experiment samples, ±SD) for each reaction condition.

c *In vitro* t^6A modification activity analysis of *ar*KEOPS reconstituted with wild-type *mj*Bud32 or the indicated mutants. Shown on left are representative HPLC profiles of nucleoside composition for each reaction (left) and quantifications (right) of average t^6A content normalized to the content of uridine (n=3 independent experiment samples, ±SD). Red lines on the graph indicate the t^6A content in each reaction if KEOPS modified one mole equivalence of tRNA.

d *In vitro* t^6A modification activity analysis of *ar*KEOPS reconstituted with wild-type *mj*Bud32 or the indicated mutants, with or without 2 mM ADP (in addition to the ATP in the reaction mix). Shown are representative HPLC profiles of nucleoside composition for each reaction.

e-f Analysis of *ar*KEOPS binding to *mj*tRNA^{Lys} in the presence of 1 mM $MgCl_2$, 1 mM $MnCl_2$ and either 1 mM ATP, ADP or AMP-PnP using (**e**) the filter binding assay or (**f**) the competitive displacement of a 647-CCA probe with increasing concentrations of unlabeled *mj*tRNA^{Lys} from *ar*KEOPS (at 0.75 μM). Displacement of the 647-CCA probe was monitored by fluorescence polarization (n=3 independent experiment samples, ±SD).

g An illustration of the allosteric relations between KEOPS subunits and tRNA.

SUPPLEMENTARY TABLES

Supplementary Table S1. Data collection and refinement statistics.

	*mj*tRNA$^{Lys}_{UUU}$	*mj*Cgi121+*mj*tRNA$^{Lys}_{UUU}$
**Data collection**	CLS 08-ID-1	IMCA-CAT-17ID
Space group	C222	P3221
Cell dimensions		
a, b, c (Å)	100.50 168.98 68.87	121.42 121.42 91.84
α, β, γ (°)	90.0, 90.0, 90.0	90.0 90.0 120.0
Resolution (Å)	49-3.11	36-3.34
R_{pim}	0.025 (0.330)	0.032 (0.471)
$I/\sigma I$	13.3 (2.1)	13.6 (1.8)
$CC_{1/2}$	0.999 (0.956)	0.999 (0.847)
Completeness (%)	99.9 (100)	100 (100)
Reflections (unique)	128102 (10895)	111555 (11643)
Redundancy	11.8 (8.9)	9.6 (9.9)
**Refinement**		
Resolution (Å)	49-3.11	36-3.34
No. reflections	10765	11611
R_{work}/ R_{free}	0.228/0.246	0.206/0.237
No. atoms		
Protein/RNA	2472	4580
B-factors		
Protein	-	117.5
RNA	157.8	175.6
R.M.S deviations		
Bond lengths (Å)	0.002	0.002
Bond angles (º)	0.456	0.430
MolProbity		
Clashscore	0.81	4.36
Ramachandran Plot		
Favored	-	94.4
Allowed	-	5.6
Outliers	-	0

Supplementary Table 2. Structure of 3’ modifications to CCA oligos

Unmodified	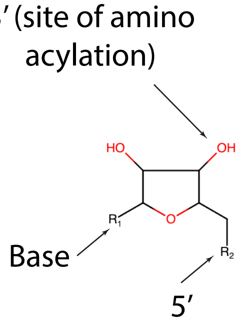
3' phosphate (3'-P)	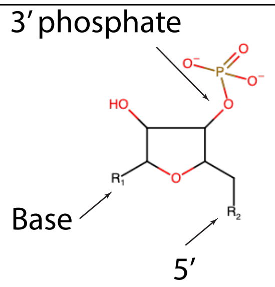
3'-C6	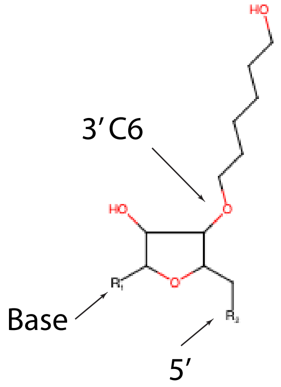
3' amine-modifier (3'-Am)	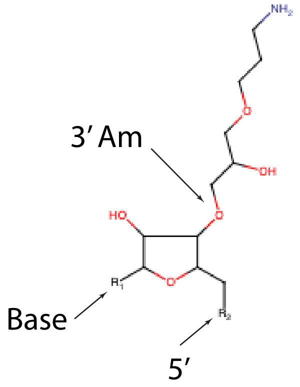

Supplementary Table 3. tRNA binding surface mutation in KEOPS components homologs.

KEOPS subunit	Species														
	M. jannaschii			*H. sapiens (cytoplasm)*			*S. cerevisiae* (cytoplasm)			*S. cerevisiae* (mitochondria)			*T. maritima*		
	Residue	mutation	Function	Residue	Mutation	Function	Residue	mutation	Function	Residue	mutation	Function	Residue	mutation	Function
Cgi121	Phe21	Lys	-	Ala33	Glu	-	Met33	Glu	-		na			na	
	Lys56	Ala	-	Arg73	Glu	-	Arg72	Ala	-						
	Met60	Glu	-	Thr77		nd	Ser76	Glu	-						
	Gln71	Ala	-	Asn88		nd	Asn88		nd						
	Ile72	Lys, Glu	-	Ile89	Glu	-	Ile89	Glu	-						
	Ile76	-	nd	Leu93	Glu	-	Phe91		nd						
	Gly80	Trp	-	Gly97		nd	Gly96	Trp	-						
Bud32	Arg60	Glu	-	Arg86		nd	Arg79	Asp	-		na			na	
	Ser148	Arg	+	Ser190		nd	Ser189	Arg	+						
	Glu152	Arg	-	Glu194		nd	Glu193	Arg	+						
	Arg251	deletion	-	Arg245		nd	Arg253	deletion	-*						
	Arg253	deletion	-	Arg247		nd	Arg255	deletion	-*						
Kae1 /Qri7 /TasD	Gln160	Glu	-	Arg163		nd	Arg195	Asp	-	Lys199		nd	Lys166	Ala	-**
	Arg163	Glu	-	Arg166		nd	Arg198	Asp	-	Arg203	Asp	-	Arg170		nd
Pcc1	Arg63	Glu	-	Arg117		nd	Arg67	Asp	-		na			na	

nd- not determined,na- non-applicable
* According to (Mao et al.; ref[23])
** According to (Luthra et al.; ref[33])

Supplementary Table 4. List of primers used in this study

mjCgi121_F21K_F	GGGGCAAGGATAAATAATGAAATTAAAAATTTAGG
mjCgi121_F21K_R	CCTAAATTTTTAATTTCATTATTTATCCTTGCCCC
mjCgi121_I72K_F	GCTTCTGGACAGAGGCAGAAACATGAGGC
mjCgi121_I72K_R	GCCTCATGTTTCTGCCTCTGTCCAGAAGC
mjCgi121_I72E_F	GGCAGATACATGAGGCAGAAAAGATTATTGGAGC
mjCgi121_I72E_R	GCTCCAATAATCTTTTTTGCCTCATGTATCTGCC
mjCgi121_G80W_F	CATGAGGCAATAAAGATTATTTGGGCTAAAGATGGG
mjCgi121_G80W_R	CCCATCTTTAGCCCAAATAATCTTTATTGCCTCATG
mjCgi121_K56A_F	CCAATAGCAGCGAGTTTTTGGATGGAAATTTTGG
mjCgi121_K56A_R	CCAAAATTTCCATCCAAAAACTCGCTGCTATTGG
mjCgi121_K56E_F	CCAATAGCAGAGAGTTTTTGGATGGAAATTTTGG
mjCgi121_K56E_R	CCAAAATTTCCATCCAAAAACTCTCTGCTATTGG
mjCgi121_M60K_F	CAAAGAGTTTTTGGAAGGAAATTTTGGTTAGAGC
mjCgi121_M60K_R	GCTCTAACCAAAATTTCCTTCCAAAAACTCTTTG
mjCgi121_M60E_F	CAAAGAGTTTTTGGGAGGAAATTTTGGTTAGAGC
mjCgi121_M60E_R	GCTCTAACCAAAATTTCCTCCCAAAAACTCTTTG
mjCgi121_Q71A_F	CTTCTGGAGCGAGGCAGATACATGAGGC
mjCgi121_Q71A_R	GCCTCATGTATCTGCCTCGCTCCAGAAG
mjCgi121_I76K_F	GGCAGATACATGAGGCAAAAAAGATTATTGGAGC
mjCgi121_I76K_R	GCTCCAATAATCTTTTTTGCCTCATGTATCTGCC
mjLys_tRNA_CCA_delete_F	CAAATCCCTTCGGGCCCGGGCCGGCATGGTCCC
mjLys_tRNA_CCA_delete_R	GGGACCATGCCGGCCCGGGCCCGAAGGGATTTG
mjLys_tRNA_CA_delete_F	CAAATCCCTTCGGGCCCGCGGCCGGCATGGTCCC
mjLys_tRNA_CA_delete_R	GGGACCATGCCGGCCGCGGGCCCGAAGGGATTTG
mjLys_tRNA_A_delete_F	CAAATCCCTTCGGGCCCGCCGGCCGGCATGGTCCC
mjLys_tRNA_A_delete_R	GGGACCATGCCGGCCGGCGGGCCCGAAGGGATTTG
mjBud32_K52A_F	GATGTAATTATTGCGGAGAGAGTTAAAAAAGGC
mjBud32_K52A_R	GCCTTTTTTAACTCTCTCCGCAATAATTACATC
mjBud32_D161R_F	CGATGTAATTCATAATCGCTTAACTACATCCAAC
mjBud32_D161R_R	GTTGGATGTAGTTAAGCGATTATGAATTACATCG
mjBud32_R60E_F	GCAAGAGAGGCAGAGTATTTAGCATTGG
mjBud32_R60E_R	CCAATGCTAAATACTCTGCCTCTCTTGC
mjBud32_S148R_F	GGTTTAGGAAAGATTAGAAATCTTGATGAAGATAAGG
mjBud32_S148R_R	CCTTATCTTCATCAAGATTTCTAATCTTTCCTAAACC
mjBud32_E152R_F	GAAAGATTTCAAATCTTGATAGAGATAAGGCAGTTG
mjBud32_E152R_R	CAACTGCCTTATCTCTATCAAGATTTGAAATCTTTC
mjBud32_R250Stop_F	GGAGTTAATGAAGGATGTTGAATGAAGAGCAAG
mjBud32_R250Stop_R	CTTGCTCTTCATTCAACATCCTTCATTAACTCC
mjBud32_G67D_F	GGTTAAAGATTTTGATATCCCAGCTCCATACATATTTG
mjBud32_G67D_R	CAAATATGTATGGAGCTGGGATATCAAAATCTTTAACC

hCgi121_A33E_F	GACTTGAGAAGAAAGGAAATGGAAGGCACCATCG
hCgi121_A33E_R	CGATGGTGCCTTCCATTTCCTTTCTTCTCAAGTC
hCgi121_R73E_F	CTGGGAAAAATGAAGACAGAAACTCTATCTACTG
hCgi121_R73E_R	CAGTAGATAGAGTTTCTGTCTTCATTTTTCCCAG
hCgi121_I89E_F	CCTTTCCCCAAATAACAATGAGTCAGAGGCTTTG
hCgi121_I89E_R	CAAAGCCTCTGACTCATTGTTATTTGGGGAAAGG
hCgi121_G97W_F	GGCTTTGAAAAAATTTTGGATCTCAGCAAATGAC
hCgi121_G97W_R	GTCATTTGCTGAGATCCAAAATTTTTTCAAAGCC
mjLys_tRNA_2nd CCA_F	GCCCGCCACCAGGCCGGCATGGTCC
mjLys_tRNA_2nd CCA_R	GGACCATGCCGGCCTGGTGGCGGGC
mjKae1_Q160D_F	GTAACTGCTTAGACGACTTTGCAAGATATGTGAATTTGC
mjKae1_Q160D_R	GCAAATTCACATATCTTGCAAAGTCGTCTAAGCAGTTAC
mjKae1_R163D_F	CTTAGACCAGTTTGCAGACTATGTGAATTTGCCACATCC
mjKae1_R163D_R	GGATGTGGCAAATTCACATAGTCTGCAAACTGGTCTAAG
mjKae1_E64R_F	GAAGTAGTTGATAAAAATAGAATTGATTTAATTGCATTCTCCC
mjKae1_E64R_R	GGGAGAATGCAATTAAATCAATCCTATTTTTATCAACTACTTC
mjKae1_K184E_F	GGCAAGGAAAGGGAAAGAACTTGTTGATTTACCTTAC
mjKae1_K184E_R	GTAAGGTAAATCAACAAGTTCTTTCCCTTTCCTTGCC
scQri7_R207D_F	GACTCATTAGATAAATGTGGCGACGAGCTTGGATTCAAG
scQri7_R207D_R	CTTGAATCCAAGCTCGTCGCCACATTTATCTAATGAGTC
scCgi121_M32E_F	GCTAAAGAAATACGCTCTAAAGAGAGTGAATTGTCG
scCgi121_M32E_R	CGACAATTCACTCTCTTTAGAGCGTATTTCTTTAGC
scCgi121_R72A_F	CAAGATGAGAACAGCAAATTTGAATTCCGAGTGCG
scCgi121_R72A_R	CGCACTCGGAATTCAAATTTGCTGTTCTCATCTTG
scCgi121_S76E_F	GAAATTTGAATGAAGAGTGCGTACTATGTCTTTCACCC
scCgi121_S76E_R	GGGTGAAAGACATAGTACGCACTCTTCATTCAAATTTC
scCgi121_I89E_F	CCCACTTCCAATGAGAGTGATGCTTTCCTCAAATTCGG
scCgi121_I89E_R	CCGAATTTGAGGAAAGCATCACTCTCATTGGAAGTGGG
scCgi121_G96W_F	GCTTTCCTCAAATTCGGAATCAAAGACGATTCG
scCgi121_G96W_R	CGAATCGTCTTTGATTCCGAATTTGAGGAAAGC
pfuPCC1_R63D_F	CTTCCGCACTGGATGGCACTGTGAACTC
pfuPCC1_R63D_R	GAGTTCACAGTGCCATCCAGTGCGGAAG
pfuPCC1_K21E_F	CCCGTCTGAAGATGTGGCTGAAGTTGTTTACG
pfuPCC1_K21E_R	CGTAAACAACTTCAGCCACATCTTCAGACGGG
pfuPCC1_L32E_F	GTTCTGTACGAACACGAATCCGTGCCGTATCG
pfuPCC1_L32E_R	CGATACGGCACGGATTCGTGTTCGTACAGAAC
scKae1_R195D_F	CGGTAATTGTCTTGATGACTTTGCAAGAACTC
scKae1_R195D_R	GAGTTCTTGCAAAGTCATCAAGACAATTACCG
scKae1_R198D_f	GATAGATTTGCAGACACTCTGAAGATACCTAATG
scKae1_R198D_R	CATTAGGTATCTTCAGAGTGTCTGCAAATCTATC
scBud_S189E_F	GGGCTCAGTTGAAAACCTGGTCGAAGATAAAGG
scBud_S189E_R	CCTTTATCTTCGACCAGGTTTTCAACTGAGCCC

scBud32_R79D_F	CGTTGAATGAGTCGGACTTATTGGCCAAATTATAC
scBud32_R79D_R	GTATAATTTGGCCAATAAGTCCGACTCATTCAACG
scBud32_E193R_F	GGGCTCAGTTTCAAACCTGGTCAGGGATAAAGG
scBud32_E193R_R	CCTTTATCCCTGACCAGGTTTGAAACTGAGCCC
scPCC1_R67D_F	GCATTGATGATAGGGTGCTTGACGTGGGAGTTAGCAG
scPCC1_R67D_R	CTGCTAACTCCCACGTCAAGCACCCTATCATCAATGC
mjtRNA_Asn_UC_F	GCCCCCATAGTCCAGATGGTAGAGCG
mjtRNA_Asn_UC_R	CGCTCTACCATCTGGACTATGGGGGC
mjtRNA_Met_UC_F	GCCGAGGTGGTCTAGCTGGTTATAGCG
mjtRNA_Met_UC_R	CGCTATAACCAGCTAGACCACCTCGGC
mjtRNA_Lys_UC_F	GGGCCCGTAGTCCAGTCTGGCAGAGC
mjtRNA_Lys_UC_R	GCTCTGCCAGACTGGACTACGGGCCC
mjtRNA_Ala_UC_F	GGGCTGGTAGTCCAGACTGGGAGAGCG
mjtRNA_Ala_UC_R	CGCTCTCCCAGTCTGGACTACCAGCCC
mjtRNA_Thr_UC_F	GCCTCGGTAGTCCAGCCTGGCGG
mjtRNA_Thr_UC_R	CCGCCAGGCTGGACTACCGAGGC

Supplementary Table 5. tRNA sequences used in this study.

species	tRNA	sequence
mj	Lys UUU	GGGCCCGUAGCUCAGUCUGGCAGAGCGCCUGGCU**UUU**AACCGGUGGUCGAGGGUUCAAATCCCUUCGGGCCCGCCA
mj	Lys UUU (ASL)	CAGAGCGCCUGGCU**UUU**AACCGGUGGG
mj	Met CUA	CGACTCACTATAGCCGAGGUGGCUUAGCUGGUUAUAGCGCCCGGCU**CAU**AACCGGGAGGUCGAGGGUUCGAAUCCCUCCCUCGGCACCA
mj	Asn GUU	CGACTCACTATAGCCCCCAUAGCUCAGAUGGUAGAGCGACGGACU**GUU**AAUCCGUAGGUCGCAGGUUCGAGUCCUGCUGGGGGCGCCA
mj	Ile GAU	AGGGCGGUGGCUCAGCCUGGUUAGAGUGCUCGGCU**GAU**AACCGAGUGGUCCGGGGUUCGAAUCCCCGCCGCCCUACCA
mj	Thr GGU	GCCUCGGUAGCUCAGCCUGGCGGAGCGCCUGCUU**GGU**AAGCAGGAGGUCGCGGGUUCAAACCCCGCCCGAGGCUCCA
mj	Arg GCG	GCCCGGGUCGCCUAGCCAGGAUAGGGCGCUGGCCU**GCG**GAGCCAGUUUUUUCAGGGGUUCAAAUCCCCUCCCGGGCGCCA
mj	Cys GCA	GCCGGGGUAGUCUAGGGGCUAGGCAGCGGACU**GCA**GAUCCGCCUUACGUGGGUUCAAAUCCCACCCCCGGCUCCA
mj	Asp GUC	GCCCUGGUGGUGUAGCCCGGCCUAUCAUACGGGACU**GUC**ACUCCCGUGACUCGGGUUCAAAUCCCGGCCAGGGCGCCA
mj	Val CAC	GGGCUCGUGGUCUAGAUGGCUAUGATGCCGCCCTGACACGGCGGTGGTCGGGAGTTCGAATCTCCCCGAGCCCACC

mj	Ala GGC	GGGCUGGUAGCUCAGACUGGGAGAGCGCCGCAUUGGCUGUGCGGAGGCCGCGG GUUCAAAUCCCGCCCAGUCCACCA
sc	Ile AAU	AGGUCUCUUGGCCCAGUUGGUUAAGGCACCGUGCU**AAU**AACGCGGGGAUCAGCG GUUCGAUCCCGCUAGAGACCA
sc	Ile AAU (ASL)	GUAAGGCACCGUGCU**AAU**AACGCGGGGC

Supplementary Table 6. Yeast strains used in this study.

Strain	Background	Genotype	Source
DDY2067	W303	*MATa ade2-1 trp1-1 can1-100 leu2-3,112 his3-11,15 ura3-1 GAL+ psi+ ssd1-d2 RAD5+ bud32::NATMX [pBUD32-URA3]*	Mao et al., ref[23]
DDY4813	W303	DDY2607 + *[pRS415]*	This study
DDY4814	W303	DDY2067 + *[pRS415-BUD32-FLAG]*	This study
DDY4815	W303	DDY2067 + *pRS415-BUD32-R79D-FLAG]*	This study
DDY4816	W303	DDY2067 + *[pRS415-BUD32-S189E-FLAG]*	This study
DDY4839	W303	*MATa ade2-1 trp1-1 can1-100 leu2-3,112 his3-11,15 ura3-1 GAL+ psi+ ssd1-d2 RAD5+ bud32::NATMX [pRS414-2xFLAG]*	This study
DDY4840	W303	*MATa ade2-1 trp1-1 can1-100 leu2-3,112 his3-11,15 ura3-1 GAL+ psi+ ssd1-d2 RAD5+ bud32::NATMX [pRS414-2xFLAG-CGI121]*	This study
DDY4841	W303	*MATa ade2-1 trp1-1 can1-100 leu2-3,112 his3-11,15 ura3-1 GAL+ psi+ ssd1-d2 RAD5+ bud32::NATMX [pRS414-2xFLAG-CGI121-I89E, G96W]*	This study
DDY4819	W303	DDY2068 + *[pRS415-2xFLAG-KAE1]*	This study
DDY4820	W303	DDY2068 + *[pRS415-2xFLAG-KAE1-R195D]*	This study
DDY4821	W303	DDY2068 + *[pRS415-2xFLAG-KAE1-R198D]*	This study
DDY1872	W303	*MATa ade2-1 trp1-1 can1-100 leu2-3,112 his3-11,15 ura3-1 pcc1-4::HIS3*	Kisseleva-Romanova et al., ref[6]
DDY4822	W303	DDY1872 + *[pRS415]*	This study
DDY4823	W303	DDY1872 + *[pRS415-PCC1-FLAG]*	This study
DDY4824	W303	DDY1872 + *[pRS415-PCC1-R67D-FLAG]*	This study
DDY572	W303	*MATa ade2-1 trp1-1 can1-100 leu2-3,112 his3-11,15 ura3-1 GAL+ psi+ ssd1-d2 RAD5+ cgi121::KANMX*	Mao et al., ref[23]

DDY4825	W303	DDY572 + *[pRS414-2xFLAG]*	This study
DDY4826	W303	DDY572 + *[pRS414-2xFLAG-CGI121]*	This study
DDY4827	W303	DDY572 + *[pRS414-2xFLAG-CGI121-I89E,G96W]*	This study
DDY4828	W303	DDY572 + *[pRS414-2xFLAG-CGI121-M33E,S76E,I89E]*	This study
DDY546	W303	*MATα ade2-1 trp1-1 can1-100 leu2-3,112 his3-11,15 ura3-1 GAL+ psi+ ssd1-d2 RAD5+ LYS+ cdc13-1 int cgi121::KANMX*	Downey et al., ref[5]

Supplementary Table 7. Yeast expression plasmids used in this study.

**Plasmid ID**	**Plasmid name**
pDD5755	pRS414-2xFLAG
pDD5756	pRS414-2xFLAG-CGI121
pDD5757	pRS414-2xFLAG-CGI121-M33E
pDD5758	pRS414-2xFLAG-CGI121-R72E
pDD5759	pRS414-2xFLAG-CGI121-S76E
pDD5760	pRS414-2xFLAG-CGI121-I89E
pDD5761	pRS414-2xFLAG-CGI121-G96W
pDD5762	pRS414-2xFLAG-CGI121-M33E, R72E
pDD5763	pRS414-2xFLAG-CGI121-M33E, S76E
pDD5764	pRS414-2xFLAG-CGI121-I89E, G96W
pDD5765	pRS414-2xFLAG-CGI121-M33E, R72E, I89E
pDD5766	pRS414-2xFLAG-CGI121-M33E, S76E, I89E
pDD5767	pRS415-BUD32-FLAG
pDD5768	pRS415-BUD32-R79D-FLAG
pDD5769	pRS415-BUD32-S189E-FLAG
pDD5770	pRS415-BUD32-E193R-FLAG
pDD5771	pRS415-2xFLAG-KAE1
pDD5772	pRS415-2xFLAG-KAE1-R195D
pDD5773	pRS415-2xFLAG-KAE1-R198D
pDD5774	pRS415-PCC1-FLAG
pDD5775	pRS415-PCC1-R67D-FLAG

Supplementary Table 8. Probe sequences used for positive hybridization assays.

.Probe	Sequence	Annealing temperature
Ile^{AAU} ASL	GATCCCCGCGTTATTAGCA	56°C
Ile^{AAU} TΨC	TGGTCTCTAGCGGGATCGAA	48°C

Met^{CAU} ASL	CGACCTTCAGATTATGAGACTG	54°C
Met^{CAU} TΨC	TGCTCCAGGGGAGGTTCGA	51°C
Val^{UAC} ASL	ATCTTCGCCGTGTAAAGGC	51°C
Val^{UAC} TΨC	TGATCCAACCGAGGTTCGAA	47°C
U5 snRNA	AAGGCCCACAGTTCTTGATGT	48°C

Supplementary Table 9. HDX data summary.

Data Set	KEOPS protein complex	
	Without tRNA	With tRNA
HDX reaction details	20 mM HEPES, 100 mM NaCl, 2 mM DTT, pD_{read} = 7.00, 25°C	
HDX time point	300 sec	
HDX control sample(s)	Please refer to the HDX-MS experimental procedure	
#of peptides	250	
Sequence Coverage	81.61%	
Average peptide length / Redundancy	11.76 / 3.43	
Replicates	4	
Repeatability	0.021 (average std. deviation in absolute deuterium uptake)	0.029 (average std. deviation in absolute deuterium uptake)
Significant differences in HDX	(+/-) 0.0763 Deuterons, 95% confidence interval	
