# Supplementary material for: A substrate binding model for the KEOPS tRNA modifying complex
Source: Nat Commun. 2020 Dec 4;11:6233. doi: 10.1038/s41467-020-19990-5 (PMC7718258; doi:10.1038/s41467-020-19990-5)
Supplement: Supplementary file 3 — Reporting Summary [file 41467_2020_19990_MOESM3_ESM.pdf]

nature research

Corresponding author(s): Frank Sicheri

Last updated by author(s): Oct 25, 2020

Reporting Summary

Nature Research wishes to improve the reproducibility of the work that we publish. This form provides structure for consistency and transparency in reporting. For further information on Nature Research policies, see our Editorial Policies and the Editorial Policy Checklist.

Statistics

For all statistical analyses, confirm that the following items are present in the figure legend, table legend, main text, or Methods section.

n/a	Confirmed	
☐	☒	The exact sample size (*n*) for each experimental group/condition, given as a discrete number and unit of measurement
☐	☒	A statement on whether measurements were taken from distinct samples or whether the same sample was measured repeatedly
☒	☐	The statistical test(s) used AND whether they are one- or two-sided<br>*Only common tests should be described solely by name; describe more complex techniques in the Methods section.*
☒	☐	A description of all covariates tested
☒	☐	A description of any assumptions or corrections, such as tests of normality and adjustment for multiple comparisons
☐	☒	A full description of the statistical parameters including central tendency (e.g. means) or other basic estimates (e.g. regression coefficient) AND variation (e.g. standard deviation) or associated estimates of uncertainty (e.g. confidence intervals)
☒	☐	For null hypothesis testing, the test statistic (e.g. *F*, *t*, *r*) with confidence intervals, effect sizes, degrees of freedom and *P* value noted<br>*Give P values as exact values whenever suitable.*
☒	☐	For Bayesian analysis, information on the choice of priors and Markov chain Monte Carlo settings
☒	☐	For hierarchical and complex designs, identification of the appropriate level for tests and full reporting of outcomes
☒	☐	Estimates of effect sizes (e.g. Cohen's *d*, Pearson's *r*), indicating how they were calculated

Our web collection on statistics for biologists contains articles on many of the points above.

Software and code

Policy information about availability of computer code

Data collection	Gen5 v2.05 (Fluorescence data collection), PhosphorImager (imaging)
Data analysis	Excel v16.16.26, GraphPad Prism v8.1.2 and v8.3 (curve fitting), PyMol v1.7.4.3 (structure rendering), XIA2-DILAS v1.14.5 (X-ray data processing), COOT v0.9 (X-ray structure model building), PHENIX v1.16 (X-ray structure model refinement), PHASER v2.8.3 (molecular replacement), AutoProc v1.0.5 (X-ray data processing), IImageQuant TL software v2005 (Image processing), NMRviewJ v9.2.0.b11 (NMR data processing), NMRpipe v20180523 (NMR data processing), Analysis v2.4.2 (NMR data analyzing), Patchdock (molecular docking), Gromacs 5.1.4 (molecular docking) , Cryosparc v2.13.0 (EM data analysis), HXpipe (MS peptide identification, available in Mass Spec Studio v2.4.0.3486), HXdeal in Mass Spec Studio v2 (MS deuterium uptake analysis), Chromeleon v6.8 (HPLC data analysis software)

For manuscripts utilizing custom algorithms or software that are central to the research but not yet described in published literature, software must be made available to editors and reviewers. We strongly encourage code deposition in a community repository (e.g. GitHub). See the Nature Research guidelines for submitting code & software for further information.

Data

Policy information about availability of data

All manuscripts must include a data availability statement. This statement should provide the following information, where applicable:
- Accession codes, unique identifiers, or web links for publicly available datasets
- A list of figures that have associated raw data
- A description of any restrictions on data availability

Coordinates and structure factors for the structures of mjtRNALysUUU and the mjCgi121-mjtRNALysUUU complex (PDB 7KJT and 7KJU respectively) are available at the Protein Data Bank as well as all other previously published PDB files that were used for this publication (https://www.rcsb.org/). NMR backbone resonance assignments for mjCgi121 were obtained from the Biological Magnetic Resonance Data Bank (https://bmrb.io/data_library/summary/?bmrbId=15981). The mass

spectrometry proteomics data have been deposited to the ProteomeXchange Consortium via the PRIDE partner repository (https://www.ebi.ac.uk/pride/archive/) with the dataset identifier PXD018007. tRNA sequences were obtained from GtRNAdb website (http://gtrnadb.ucsc.edu/). All relevant data supporting the findings in this study are provided within this article or its Supplementary files or from the corresponding author upon reasonable request. Source data are provided with this paper.

Field-specific reporting

Please select the one below that is the best fit for your research. If you are not sure, read the appropriate sections before making your selection.

- [x] Life sciences
- [ ] Behavioural & social sciences
- [ ] Ecological, evolutionary & environmental sciences

For a reference copy of the document with all sections, see nature.com/documents/nr-reporting-summary-flat.pdf

Life sciences study design

All studies must disclose on these points even when the disclosure is negative.

Sample size	Statistical methods were not used to determine sample sizes.<br><br>However, for quantification purposes, a sample size of n = 2 unless otherwise stated (reproduced two or more independent times) was used to assess reproducibility and robustness of each experiment performed. Sample sizes were based on prior experience in the field.
Data exclusions	No data was excluded from analysis
Replication	To ensure reproducibility of experimental findings, all biochemical or cellular assays were repeated independently at least two times. One representative result for each experiment is presented in the main figures or the supplementary figures. The results from all experiments repeating those in the paper were essentially the same as those presented in the paper.
Randomization	Randomization for experiments was not relevant because all protein and RNA samples and yeast cells used for analysis were from the same initial stocks.
Blinding	Blinding for experiments was not relevant because all data collection and analysis were quantitative and not qualitative in nature.

Reporting for specific materials, systems and methods

We require information from authors about some types of materials, experimental systems and methods used in many studies. Here, indicate whether each material, system or method listed is relevant to your study. If you are not sure if a list item applies to your research, read the appropriate section before selecting a response.

Materials & experimental systems

n/a	Involved in the study	
[ ]	[x]	Antibodies
[ ]	[x]	Eukaryotic cell lines
[x]	[ ]	Palaeontology and archaeology
[x]	[ ]	Animals and other organisms
[x]	[ ]	Human research participants
[x]	[ ]	Clinical data
[x]	[ ]	Dual use research of concern

Methods

n/a	Involved in the study	
[x]	[ ]	ChIP-seq
[x]	[ ]	Flow cytometry
[x]	[ ]	MRI-based neuroimaging

Antibodies

Antibodies used	anti-DDDDK tag (abcam, ab1257) and anti-pGK1 (abcam, ab113687).
Validation	Antibodies were validated for their specificity by the source companies

Eukaryotic cell lines

Policy information about cell lines

Cell line source(s)	Yeast strains that were used in this study were generated in the Sicheri and Durocher labs based on a W303 background.
Authentication	Strains were authenticated by PCR genotyping. Plasmids used for strain transformation were verified by sequencing.
Mycoplasma contamination	Not relevant for yeast cell cultures.

Commonly misidentified lines (See ICLAC register)	No Commonly misidentified lines were used in this study.
