# Supplementary material for: A substrate binding model for the KEOPS tRNA modifying complex
Source: Nat Commun. 2020 Dec 4;11:6233. doi: 10.1038/s41467-020-19990-5 (PMC7718258; doi:10.1038/s41467-020-19990-5)
Supplement: Supplementary file 4 — Description of Additional Supplementary Files [file 41467_2020_19990_MOESM4_ESM.pdf]

Description of Additional Supplementary Files

File name: Supplementary Data 1
Description: Differential deuteration data collected from the KEOPS complex, with and without added tRNA. All detectable peptides for each of the four subunits are shown, along with measured charge state (z), m/z and retention time (RT). Deuteration data show raw output (deuterium shift, in Da) with standard deviations (n=4 independent labeling experiment using the same protein stock). Data presented according to Gothenburg Guidelines (Masson GR et al, 2019, Nat Methods, 16, 595-602).
