# Supplementary material for: A substrate binding model for the KEOPS tRNA modifying complex
Source: Nat Commun. 2020 Dec 4;11:6233. doi: 10.1038/s41467-020-19990-5 (PMC7718258; doi:10.1038/s41467-020-19990-5)
Supplement: Supplementary file 2 — Peer Review File [file 41467_2020_19990_MOESM2_ESM.pdf]

Reviewer #1 (Remarks to the Author):

Comments to the authors:

This paper presents a series of biochemical and structural analysis on the KEOPS complex, which is responsible for introducing the t5A37 modification to tRNA species with the ANN anticodon. The t5A37 modification is important for human health, because mutations in various components of the KEOPS complex lead to Galloway-Mawat syndrome, which is a severe autosomal-recessive disease that manifests in developmental defects, leading to early childhood death.

The KEOPS complex consists of 4 core components in archaea, Kae1 (responsible for the t5A37 synthesis), Bud32 (an ATPase), Cgi121, and Pcc1. Each component of the complex has a homolog in human (TPRKB, PRPK, OSGEP, and LAGE3, respectively), with the addition of the Gon7 component to the human complex. The t5A37 modification is performed by a single homodimer enzyme Ori7 in mitochondria, and by a simpler complex of TsaD (homolog of Kae1) and TsaB-TsaE in bacteria. The complexity of the various complexes makes the study of the t5A37 modification challenging. This work focuses on the archaeal KEOPS complex, and draws in comparison with the human, mitochondrial, and bacterial complexes. Overall, the paper provides a convincing story, based on the solved crystal structure of the Cgi121 component in complex with a tRNA substrate, that the Cgi121 of the archaeal complex is responsible for recognizing the CCA end of the tRNA substrate as a mechanism to recruit the substrate to the complex and to align all other components to position the target nucleotide A37 to the active site of the Kae1 enzyme for t5A37 modification.

Several critical areas need to be clarified and addressed.
1. The Introduction section should include a statement on what the function is for each component of the KEOPS complex. Without knowing this information, it is hard to follow the manuscript.

2. The data in Figure 1d are difficult to understand. Residues that are hyper-perturbed by tRNA binding in the profile of chemical shift perturbation (CSP) and in the profile of peak intensity changes do not correlate.

3. In Supplementary Figure 1b, the plot of normalized t5A vs. each component of the complex, what is the unit of the normalized t5A? Is it a percent, or a concentration unit? This problem applies to all of the mass spec analysis of the modification.

4. Page 5, lines 132-138, some sentences are missing here. Please revise this paragraph.

5. A major issue of this work is that most of the tRNA binding data are not quantitative, based on the filter binding assay (e.g., Figures 1 and 2, and others). This lack of quantification makes the work very “descriptive” and not precise. The authors did have a quantitative assay, using the Alex647-CCA substrate. Is it possible to adopt the Alex647 strategy to validate some of the filter binding assay data?

6. In the discussion of Bud32 to tRNA binding (line 194), the authors stated that “mjBud32 may also participate in direct binding to tRNA”. This is ambiguous. Bud32 by itself does not bind tRNA (Figure 1A), but it promotes Cgi121 to bind tRNA.

7. The human homolog TPRKB binds to the Alex647-CCA substrate much weaker (Kd = 28 μM) than the Cgi protein (Kd = 3.9 μM), but the effect of PRPK on enhancing tRNA binding is stronger (60-fold) than Bud32 (5-fold). A comment should be made on how this is the case. What is the difference between the human protein and the archaeal protein?

8. The composite KEOPS-tRNA model, together with the single-molecule negative stain EM, is very nice.

9. The comment that the KEOPS-tRNA composite model resembles the TobZ-tobramycin complex is unclear. Is the 7.3 A distance between the N6 acceptor of A37 and the donor? This distance appears too long for a molecular interaction to enable chemistry to take place (Figure 4d).

10. It was mentioned that mito Qri7, or the bacterial TsaB-TsaD complex, is unlikely to bind to the CCA sequence. This statement should be verified by experiments.

11. The rationale for testing the function of Cgi121 in yeast is not easy to understand. What is the biology that cgi121 deletion suppresses the temperature-sensitive growth phenotype of the cdc13-1 allele?

12. In vivo data (Figure 6e-f) should be complemented by mass spec analysis of the t5A37 modification.

13. The relationship between the ATPase of Bud32 and the t5A37 modification of Kae1 can be more clearly presented. If the ATPase of Bud32 is not to assist in product release, then provide a clear hypothesis or model how it is associated with the t5A37 modification of Kae1. Also, make a clear presentation of how the ATPase of bud32 is coupled to the t5A37 modification.

14. The last paragraph of Discussion, the closing paragraph, is hard to read. The main message of the paragraph is buried in details and is distracted. It should be rewritten.

15. Many figures have no error bars, yet an error "±" is presented. For example, Figure 3E, F, and G, there are no error bars, but each Kd value is presented with a "±". Is the ± from curve fitting?

16. Figure 7A, Y axis is luminescence. It may be easier to show relative units.

Reviewer #2 (Remarks to the Author):

In this manuscript, Beenstock et al determined the crystal structure of the Cgi121-tRNA complex, and investigated how the KEOPS complex recognizes its substrate tRNA, especially how Cgi121, a domain of the KEOPS complex, recruits tRNA by binding its 3′-CCA end. The experiments and the point of an argument are clear, and, therefore, this manuscript is suitable to be published in Nature Communications after minor revisions.

Minor points
1. In line 187, there is a text "through an allosteric effect". However, the experiment did not reject a possibility that Bud32 directly binds to the 5′ end of 647-CCA. In addition, the effect of an Alexa-647 tag should be also incorporated.
2. In the paragraph from line 314, tRNA recognitions by the KEOPS orthologues, the Qri7 dimer and TsaD-TsaB, is discussed. The discussion itself looks reasonable, but there is no discussion about how similar the homologous proteins are by any values. Not only showing their ribbon models, but also showing any calculated values (e.g. r.m.s.d.) should be favorable.

Other very minor points
1. In the words of "5′" and "3′", "prime" and "apostrophe" are mixed. All should be "prime".
2. All "Extended Data Fig." should be replaced by "Supplementary Fig.".
3. Line 294, not "Fig. 5a", but "Supplementary Fig. 5a".
4. Line 302, not "Fig. 5b", but "Supplementary Fig. 5b".
5. In Supplementary Table 1, "mjtRNALysUU" should be "mjtRNALysUUU".

6. In Supplementary Table 1, “C”of the space group “C222” should be italic. The same for “P3221”. In addition, “2” next to “3” should be subscript.
7. In supplementary Table 1, the resolution regions of parentheses are not shown.
8. In all text and Figures, “K” of “Kd” (dissociation constant) should be italic.

Reviewer #3 (Remarks to the Author):

The article titled “A substrate binding model for the KEOPS tRNA modifying complex” by Beenstock et al., has uncovered how KEOPS modifying enzyme operates. The authors have provided one of the first models of how tRNA is recognized by the KEOPS complex using multiple techniques (in vitro and in vivo). Interestingly, the authors also highlighted the differences and the commonalities of t6A modifying enzymes across domains of life. Overall, the work provides key insights into the understanding of how KEOPS machinery operates. However, certain proposals in the manuscript are not well supported and needs to be addressed before publication as given below.

Major concerns:

1. In figure 7G, the authors summarize the overall work using a model. According to which Cgi121 first binds to the tRNA and recruits itself along with tRNA to the Bud32-Kae1-Pcc1 complex. However, this model needs supporting evidence that physiologically Cgi121 exists independently and forms complex only upon binding to tRNA.

2. In line 125, the authors state “The ribose 3' hydroxyl group of A76 is tightly packed against Cgi121, which may disfavor the binding of charged tRNAs”. However, in figure 2 it appears that the 2’ and 3’ hydroxyl groups are projecting away from the Cgi121 surface. More importantly, the authors therefore need to experimentally validate the statement by showing that Cgi121 cannot bind aa-tRNA. This is a relatively straightforward experiment and would go a long way in proving the validity of the mode of tRNA binding. Otherwise, the major result of the manuscript i.e. the crystal structure of Cgi121 with tRNA could turn out to be a crystallographic artifact!

3. In the same vein, since the authors have already established the methodology of obtaining different complexes, a relatively simpler SAXS based analysis could have helped to validate the proposed model.

4. Also, the authors seems to have summarized all the experiments that they have performed rather than the final critical ones, for example the initial NMR data is not strong enough and it is not required in the context of the crystal structure/model as described later. Same is true for their mutational data where they could use a logic for the mutation(see below) and only summarize the critical ones!

Minor comments:

1. One of the major findings of this work is the crystal structure of Cgi121 in complex with tRNA, on which the entire story builds. Therefore, the authors need to provide a stereo image of an electron density (unbiased) map for the CCA end of the tRNA.

2. In line 98, the authors state “Furthermore, sub-complexes containing mjCgi121 demonstrated tRNA binding, while those lacking mjCgi121 did not (Fig. 1b)”. However, the figure does not have the tRNA binding data with Cgi121 in complex with Kae1.

3. In Supplementary Table 1, the following changes are required:
a) The space group should be written as ‘P 3221’ instead of ‘P3221’.
b) Units of B factors?

c) Also, describe Rfree

4. In line 120 the authors state "…compared to the structure of mjCgi121 without tRNA (RMSD =1.6 Å)", the number of Cα atoms used for the superpositioning is not mentioned.
5. The authors have done extensive mutational studies.
The rationale for Cgi121 mutations such as M60E, F21K, G80W, I72K is not obvious and needs to be explained. However, throughout the article, the authors have not commented on the conservation of these residues, and no structure-based sequence alignments are provided.

6. In line 265 the authors state "the N6 acceptor site of A37 lies only 7.3 Å from the corresponding acceptor site (hydroxyl group of C6'') of tobramycin". What is the distance from the donor atom? this needs to be sown in the figure as well.

7. In multiple instances (line 276, 277, 299, 301), the authors specify the exact atoms of tRNA interacting with the KEOPS complex. Since the mutational studies of the full complex is based on a model supported with negative stain, and in the absence of an atomic structure, the authors may avoid specifying such intricate interactions.

8. In figure 7c, along with the ANN containing tRNAs the Cgi121-Bud32-Kae1 complex shows equal ATPase activity also with the non-ANN containing tRNAAla, which is perplexing and needs an explanation for this erroneous and non-specific binding and indicative of ANN is not the sole discriminator.

9. Can the authors comment on what is the exact role of Pcc1?

10. Throughout the article, the authors have subdivided the section into small paragraphs. The authors may consider avoiding too small (3 lines) paragraphs by merging with the earlier or later.

11. As per the current model, the KEOPS recognizes the CCA but not ΔCCA and 2X-CCA, which is essential to modify only the correctly folded tRNAs. However, the mitochondrial (Qri7) and bacterial (TsaB, D, E) systems modify tRNAs irrespective of the CCA terminal which can be erroneous. Can the authors explain the physiological relevance of such a mechanism in bacteria and mitochondria?

12. In extended data Fig 3C, the authors need to provide SEC profiles for apo protein and free tRNA for comparison.

Reviewer #4 (Remarks to the Author):

Author Comments:

Overall, the authors have conducted an excellent and thorough analysis, and the methods employed and the means of reporting the data are all adherent to community HDX guidelines. Furthermore, the authors are applauded for making their data available on PRIDE.

A couple of queries:
1) From the Woods Plot in figure SF6D, it appears that there is little peptide coverage of the first 50 amino-acids of Kae1. Do the authors believe this could impact on their interpretation of the data?
2) Also from SF6D, there are 2 peptides at the very N-terminus of Bud32 which see significant enhancements of HDX (>-0.2 delta value). This seems at odds with the other peptides within this region. The authors have not shown these as significant changes (as they are not coloured blue (as is my understanding). Why were these peptides excluded from the results? Given the magnitude of the difference, I would expect these to be significant - Why were these peptides excluded from the

results?
Minor:
1) Supplementary Table S8: Table headers are not visible (charge, Sequence Start).
2) Supplementary Table S8: Units of RT are not shown

Glenn Masson

Reviewer #1 (Remarks to the Author):

Comments to the authors:

This paper presents a series of biochemical and structural analysis on the KEOPS complex, which is responsible for introducing the t5A37 modification to tRNA species with the ANN anticodon. The t5A37 modification is important for human health, because mutations in various components of the KEOPS complex lead to Galloway-Mawat syndrome, which is a severe autosomal-recessive disease that manifests in developmental defects, leading to early childhood death.

The KEOPS complex consists of 4 core components in archaea, Kae1 (responsible for the t5A37 synthesis), Bud32 (an ATPase), Cgi121, and Pcc1. Each component of the complex has a homolog in human (TPRKB, PRPK, OSGEP, and LAGE3, respectively), with the addition of the Gon7 component to the human complex. The t5A37 modification is performed by a single homodimer enzyme Ori7 in mitochondria, and by a simpler complex of TsaD (homolog of Kae1) and TsaB-TsaE in bacteria. The complexity of the various complexes makes the study of the t5A37 modification challenging. This work focuses on the archaeal KEOPS complex, and draws in comparison with the human, mitochondrial, and bacterial complexes. Overall, the paper provides a convincing story, based on the solved crystal structure of the Cgi121 component in complex with a tRNA substrate, that the Cgi121 of the archaeal complex is responsible for recognizing the CCA end of the tRNA substrate as a mechanism to recruit the substrate to the complex and to align all other components to position the target nucleotide A37 to the active site of the Kae1 enzyme for t5A37 modification.

Several critical areas need to be clarified and addressed.
1. The Introduction section should include a statement on what the function is for each component of the KEOPS complex. Without knowing this information, it is hard to follow the manuscript.

The functional roles of the KEOPS subunits, with exception of the tRNA modifying enzyme Kae1, was almost completely unknown until this study. We have emphasized this point further in the introduction (page 3 lines 72-77). Specifically, we state:

> ‘ Interestingly, despite being required for tRNA modification, the precise roles of Bud32, Cgi121 and Pcc1 are barely known. The ATPase activity of Bud32 is potentiated by tRNA[21,22] and by direct binding to Cgi121[23] and is essential for t^6A catalytic activity[22]. Pcc1, which binds directly to Kae1, can mediate the dimerization of KEOPS[23,24]. However this activity of Pcc1 is not required for t^6A catalytic activity[24] and is obstructed in eukaryotes by Gon7 binding to Pcc1[7,8,25].’

2. The data in Figure 1d are difficult to understand. Residues that are hyper-perturbed by tRNA binding in the profile of chemical shift perturbation (CSP) and in the profile of peak intensity changes do not correlate.

To address the issue raised, in revised **Fig. 1d** we now highlight the cohort of residues in red that meet the criteria of being hyper-perturbed by tRNA. Hyper-perturbed residues include those with a CSP ≥ 0.02 ppm (upper graph) as well as residues with a peak intensity change of ≤ 0.4 (lower graph).

In addition, we now provide a schematic of the secondary structure of mjCgi121 beneath the graphs highlighting the position of the hyper-perturbed residues in mjCgi121. For further clarification, we have added the residue numbers of α-helices 1, 3 and 4 to **Fig. 1e** to ease comparison with the data shown in **Fig. 1d**.

3. In Supplementary Figure 1b, the plot of normalized t5A vs. each component of the complex, what is the unit of the normalized t5A? Is it a percent, or a concentration unit? This problem applies to all of the mass spec analysis of the modification.

We believe that this comment refers to the HPLC analysis of nucleoside composition we conducted. In this analysis, nucleosides were separated based on hydrophobicity on a reverse phase column and elution profiles were obtained by measuring absorption at 254 nm. The peak corresponding to t6A is identified by retention time using a commercially available t6A nucleoside as an external standard. In previous publications from our lab we have verified by mass spectrometry identification that the peak that elutes according to the reference molecule is indeed t6A (Wan et al., NAR, 2013).

Normalized t6A presented in the paper is the ratio between the t6A and uridine content in each sample analyzed. Uridine content was chosen as a reference as an internal standard for each HPLC run because it is not influenced by KEOPS activity (which affects unmodified adenosine composition) or deletion of the CCA tail (which affects the composition of cytosine and adenosine). Thus the t6A/uridine ratio allows us to directly compare experiments using different tRNA samples. Assay conditions are set so that only 40-60% of the substrate is modified by the KEOPS complex containing WT proteins and thus substrate is not limiting under reaction conditions.

4. Page 5, lines 132-138, some sentences are missing here. Please revise this paragraph.

We have modified the paragraph in question (page 5 lines 142-143, page 6 lines 144-151) to be clearer.

5. A major issue of this work is that most of the tRNA binding data are not quantitative, based on the filter binding assay (e.g., Figures 1 and 2, and others). This lack of quantification makes the work very “descriptive” and not precise. The authors did have a quantitative assay, using the Alex647-CCA substrate. Is it possible to adopt the Alex647 strategy to validate some of the filter binding assay data?

We have validated our data from the filter-binding assay using the 647-CCA probe as suggested by the reviewer. The new data are shown in **Fig. 3e**, **Supplementary Fig. 5d** and **Supplementary Fig. 9f**. All new data is fully consistent with our previous results.

6. In the discussion of Bud32 to tRNA binding (line 194), the authors stated that “mjBud32 may also participate in direct binding to tRNA”. This is ambiguous. Bud32 by itself does not bind tRNA (Figure 1A), but it promotes Cgi121 to bind tRNA.

We thank the reviewer for pointing out this ambiguity. A revised statement can be found on page 7 lines 205-206.

7. The human homolog TPRKB binds to the Alex647-CCA substrate much weaker (Kd = 28 μM) than the Cgi protein (Kd = 3.9 μM), but the effect of PRPK on enhancing tRNA binding is stronger (60-fold) than Bud32 (5-fold). A comment should be made on how this is the case. What is the difference between the human protein and the archaeal protein?

We agree with this reviewer that although the archaeal and human proteins have the same trend, i.e. Bud32 enhances the binding affinity of Cgi121 to tRNA, the effect in the human proteins in much stronger. We currently do not know why this effect is more pronounced with the human proteins. We have revised the text accordingly to emphasize this point (page 9 lines 245-247).

8. The composite KEOPS-tRNA model, together with the single-molecule negative stain EM, is very nice.

We thank the reviewer for this comment.

9. The comment that the KEOPS-tRNA composite model resembles the TobZ-tobramycin complex is unclear. Is the 7.3 A distance between the N6 acceptor of A37 and the donor? This distance appears too long for a molecular interaction to enable chemistry to take place (Figure 4d).

We agree with the reviewer's comment that the position of A37 in our model is not favorable for theronycarbamoyl transfer activity and we accordingly do not propose details of the catalytic mechanism of Kae1. However, our model positions A37 7.3 Å away from the corresponding accepting site in tobramycin. We have reasoned that this distance could be accommodated by local conformational changes of the tRNA. We have revised our statements for clarity (page 10 lines 287-289).

10. It was mentioned that mito Qri7, or the bacterial TsaB-TsaD complex, is unlikely to bind to the CCA sequence. This statement should be verified by experiments.

In our current submission we show that Qri7 (unlike Cgi121 that serves as a control) does not bind to 647-CCA probe (**Supplementary Fig. 3h**). This result complements our data from the original submission showing that Qri7 binds tRNA regardless of the CCA tail deletion using the filter binding assay (**Fig. 3h**) and ultimately can t6A-modify tRNA WT and tRNA ΔCCA in a comparable manner (**Fig. 3i**).

We further provide new results showing that recombinant purified *E. coli* TsaB-TsaD-TsaE can t6A modify tRNA WT and tRNA ΔCCA in a comparable manner (**Fig. 3i**).

These results support our claim that dependency on the CCA tail is a unique feature of KEOPS within the family of t6A modifying enzymes.

11. The rationale for testing the function of Cgi121 in yeast is not easy to understand. What is the biology that cgi121 deletion suppresses the temperature-sensitive growth phenotype of the cdc13-1 allele?

We chose to use yeast cells as a cellular system for analyzing the effect of KEOPS mutations in vivo since the knockout and mutation of KEOPS genes in yeast have well established phenotypes, unlike the case with human genes for example. The biology that underlies the suppression of the temperature-sensitivity of the *cdc13-1* allele by knockout of the *CGI121* gene is interesting but largely unknown apart from the fact that it impacts the exonucleolytic degradation of uncapped telomeres (see Downey et al., Cell, 2006). We have added this clarification to the manuscript (page 13 line 387).

The growth phenotypes of the *cdc13-1*, *cgi121*Δ strain are used as a readout for the functionality of Cgi121 proteins in vivo.

12. In vivo data (Figure 6e-f) should be complemented by mass spec analysis of the t5A37 modification.

As requested, we now provide a global analysis of t6A levels in the yeast strains using the HPLC assay to complement **Fig. 6e-f**. We chose to use an HPLC analysis rather than mass spectrometry as this method is widely used to assess t6A levels in the field (examples include Deutsch et al., J Biol Chem, 2012; Wan et al., NAR, 2013, Thiaville et al., Microb Cell, 2016). In addition, as noted above in response to comment #3 made by this reviewer, we believe that the reviewer meant the HPLC assay when he/she referred to the “mass spec” assay (**Supplementary Fig. 7i**). The results from these experiments confirm the findings from the positive hybridization assays.

13. The relationship between the ATPase of Bud32 and the t5A37 modification of Kae1 can be more clearly presented. If the ATPase of Bud32 is not to assist in product release, then provide a clear hypothesis or model how it is associated with the t5A37 modification of Kae1. Also, make a clear presentation of how the ATPase of bud32 is coupled to the t5A37 modification.

We now provide a clear hypothesis in response to this comment (page 18 lines 544-551). Briefly, we hypothesize that Bud32 ATPase activity serves to regulate Kae1 activity. A possible mechanism would be that following activation of Bud32 by substrate binding, Bud32 ATPase activity in turn activates Kae1 thereby enabling the t6A modification of the bound tRNA substrate by Kae1.

14. The last paragraph of Discussion, the closing paragraph, is hard to read. The main message of the paragraph is buried in details and is distracted. It should be rewritten.

We have revised this paragraph according to the reviewer’s comments. The main message of the paragraph in question is that some aspects of how KEOPS engages tRNA substrates, specifically the Kae1 subunit, may have relevance for how other t6A modifying enzymes (Qri7 and TsaD) engage tRNA substrates

15. Many figures have no error bars, yet an error “±” is presented. For example, Figure 3E, F, and G, there are no error bars, but each Kd value is presented with a “±”. Is the ± from curve fitting?

We thank the reviewer for pointing out this confusing point.

Each binding experiment was performed with two technical replicates for every data point. For each pair of technical replicates, the average value and error bars are displayed on the graphs. However, the error value in most cases was small and therefore the bars are “too small” to visualize.

For *K*d and IC_{50} assignments, the binding profiles (performed in technical duplicate) were each repeated in triplicate. A *K*d or IC_{50} was assigned for each separate binding profile. K_d and IC_{50} values presented in the paper are the average ± SD of the three independent binding experiments. A clarification on how K_d and IC_{50} values were calculated is added to the revised materials and methods section (page 47 lines 1239-1240 and lines 1246-1248).

16. Figure 7A, Y axis is luminescence. It may be easier to show relative units.

In the revised version of **Fig. 7, Supplementary Fig. 8** and **Supplementary Fig. 9,** we now present the units of the ADP Glo assay as "Luminescence (RLU)".

Reviewer #2 (Remarks to the Author):

In this manuscript, Beenstock et al determined the crystal structure of the Cgi121-tRNA complex, and investigated how the KEOPS complex recognizes its substrate tRNA, especially how Cgi121, a domain of the KEOPS complex, recruits tRNA by binding its 3’-CCA end. The experiments and the point of an argument are clear, and, therefore, this manuscript is suitable to be published in Nature Communications after minor revisions.

We thank the reviewer for the kind statement in qualified support for the publication of our manuscript in Nature Communications.

Minor points

1. In line 187, there is a text “through an allosteric effect”. However, the experiment did not reject a possibility that Bud32 directly binds to the 5’ end of 647-CCA.

To eliminate the possibility that the enhanced affinity of the Bud32-Cgi121 complex towards the 647-CCA probe relative to Cgi121 alone is not a result of Bud32 directly binding to the probe, we show in **Fig. 3e** that Bud32 does not bind the 647-CCA probe when Cgi121 is absent. In this same new experiment, we also show that Kae1 and Pcc1 alone do not bind the 647-CCA probe either. Together, these experiments rule out the possibility that any of the KEOPS subunits besides Cgi121 binds to the 5’ end of 647-CCA on their own, and thus enhanced binding is likely attributable to an allosteric effect of Bud32 on Cgi121.

In addition, the effect of an Alexa-647 tag should be also incorporated.

To further support our contention that the binding mechanism of Cgi121 and larger assemblies of KEOPS subunits for the 647-CCA probe is reflective of how these complexes actually bind tRNA and to ensure that the presence of the Alexa-647 tag does not cause artifactual binding that is not reflective of tRNA binding, we showed that mutations in Cgi121 that disrupt full length tRNA binding in the filter binding assay (**Fig. 3A**) also disrupt the ability of Cgi121 to bind the 647-CCA probe (**Supplementary Fig. 3e**).

2. In the paragraph from line 314, tRNA recognitions by the KEOPS orthologues, the Qri7 dimer and TsaD-TsaB, is discussed. The discussion itself looks reasonable, but there is no discussion about how similar the homologous proteins are by any values. Not only showing their ribbon models, but also showing any calculated values (e.g. r.m.s.d.) should be favorable.

We agree that adding the r.m.s.d values contribute to critical reading of the paper. These values are now added (page 12 lines 346-347).

Other very minor points

1. In the words of “5’” and “3’”, “prime” and “apostrophe” are mixed. All should be “prime”.

We have fixed this in the current version. We thank the reviewer for pointing this out.

2. All “Extended Data Fig.” should be replaced by “Supplementary Fig.”.

We have fixed this in the current version. We thank the reviewer for pointing this out.

3. Line 294, not “Fig. 5a”, but “Supplementary Fig. 5a”.

We have fixed this in the current version. We thank the reviewer for pointing this out.

4. Line 302, not “Fig. 5b”, but “Supplementary Fig. 5b”.

We have fixed this in the current version. We thank the reviewer for pointing this out.

5. In Supplementary Table 1, “mjtRNALysUU” should be “mjtRNALysUUU”.

We have fixed this in the current version. We thank the reviewer for pointing this out.

6. In Supplementary Table 1, “C”of the space group “C222” should be italic. The same for “P3221”. In addition, “2” next to “3” should be subscript.

We have fixed this in the current version. We thank the reviewer for pointing this out.

7. In supplementary Table 1, the resolution regions of parentheses are not shown.

We have added the missing resolution range information in the revised manuscript.

8. In all text and Figures, “K” of “Kd” (dissociation constant) should be italic.
We have fixed this in the current version. We thank the reviewer for pointing this out.

Reviewer #3 (Remarks to the Author):

The article titled “A substrate binding model for the KEOPS tRNA modifying complex” by Beenstock et al., has uncovered how KEOPS modifying enzyme operates. The authors have provided one of the first models of how tRNA is recognized by the KEOPS complex using multiple techniques (in vitro and in vivo). Interestingly, the authors also highlighted the differences and the commonalities of t6A modifying enzymes across domains of life. Overall, the work provides key insights into the understanding of how KEOPS machinery operates. However, certain proposals in the manuscript are not well supported and needs to be addressed before publication as given below.

Major concerns:

1. In figure 7G, the authors summarize the overall work using a model. According to which Cgi121 first binds to the tRNA and recruits itself along with tRNA to the Bud32-Kae1-Pcc1 complex. However, this model needs supporting evidence that physiologically Cgi121 exists independently and forms complex only upon binding to tRNA.

We proposed that Cgi121 in isolation might exist in the cell bound to tRNA because of the finding that Cgi121 expressed in bacteria co-purifies with tRNA (**Supplementary Fig. 3c**). To further test this model, we now show using immunoprecipitation that Cgi121 is associated with tRNA in *bud32*Δ yeast cells (**Supplementary Fig. 7j-k**). Since Bud32 "links" Cgi121 to the rest of the KEOPS complex, the absence of Bud32 would prohibit association of Cgi121 with KEOPS and thus shows that Cgi121 can exist in a tRNA-bound state independent of KEOPS in yeast cells.

However, we do not rule out the possibility (particularly in species where Bud32 and Kae1 are not fused into a single poly peptide) that Cgi121 and Bud32 work together to bind tRNA prior to assembling into KEOPS since this binary complex displays tighter binding affinity for tRNA than Cgi121 alone.

In regard to the point that Cgi121 forms a complex with KEOPS only upon binding to tRNA, we show in **Supplementary Fig. 1a** and **Supplementary Fig. 3a** that Cgi121 can form a tight stoichiometric complex with Bud32 and the other KEOPS subunits independent of tRNA (page 18 lines 526-529).

2. In line 125, the authors state “The ribose 3' hydroxyl group of A76 is tightly packed against Cgi121, which may disfavor the binding of charged tRNAs”. However, in figure 2 it appears that the 2’ and 3’ hydroxyl groups are projecting away from the Cgi121 surface. More importantly, the authors therefore need to experimentally validate the statement by showing that Cgi121 cannot bind aa-tRNA. This is a relatively straightforward experiment and would go a long way in proving the validity of the mode of tRNA binding. Otherwise, the major result of the manuscript i.e. the crystal structure of Cgi121 with tRNA could turn out to be a crystallographic artifact!

In the revised manuscript we have added a surface representation of the binding interface between Cgi121 and $tRNA^{Lys}_{UUU}$ (**Supplementary Fig. 2d**). This image better highlights the predicted steric clash between the binding pocket for A76 and bulky groups added to the amino-acylation site. We thank the reviewer for pointing this out.

To test whether a bulky amino acid attached to the 3’ hydroxyl of the tRNA does in fact hinder binding to Cgi121, we used a competitive displacement assay using fluorescence polarization. Specifically, we now show that oligonucleotides corresponding to the CCA tail cannot displace the 647-CCA probe if they harbor 3’ chemical modifications. All 3’ modifications tested are either comparable in size to amino acids or smaller including a 3’ phosphate, a 3’ hexanediol (a chain of

six carbons that ends with an OH group) and a 3’ amine modifier (a chain of six carbons with an internal ether bond that ends with a primary amine) (**Supplementary Fig. 3g**). This result supports the contention that aminoacylation will decrease the ability of Cgi121 (and hence KEOPS) to bind tRNA.

3. In the same vein, since the authors have already established the methodology of obtaining different complexes, a relatively simpler SAXS based analysis could have helped to validate the proposed model.

We found that a major limitation of using SAXS for our study was that the binding affinity between KEOPS and tRNA was not sufficiently high to produce a single species in solution required for SAXS analysis. KEOPS samples with tRNA exist as a mixture of KEOPS alone, tRNA alone and KEOPS bound to tRNA species. This complexity prohibits accurate modeling of molecular envelopes. Therefore we used the orthogonal methods of single particle negative stain EM, HDX MS and mutational analysis to validate our model.

4. Also, the authors seems to have summarized all the experiments that they have performed rather than the final critical ones, for example the initial NMR data is not strong enough and it is not required in the context of the crystal structure/model as described later. Same is true for their mutational data where they could use a logic for the mutation (see below) and only summarize the critical ones!

We respectfully disagree with this point. In our view, one of the strengths of this study in our view is that the findings from many different techniques point to the same conclusions. However, if there is consensus by the reviewers and the editor for a shortening of the manuscript, we would consider doing so.

Minor comments:

1. One of the major findings of this work is the crystal structure of Cgi121 in complex with tRNA, on which the entire story builds. Therefore, the authors need to provide a stereo image of an electron density (unbiased) map for the CCA end of the tRNA.

As suggested we now provide an unbiased stereo image of the CCA tail electron density in **Supplementary Fig. 2b**.

2. In line 98, the authors state “Furthermore, sub-complexes containing mjCgi121 demonstrated tRNA binding, while those lacking mjCgi121 did not (Fig. 1b)”. However, the figure does not have the tRNA binding data with Cgi121 in complex with Kae1.

The rationale of the binding experiments was to test the binding activity of KEOPS proteins that directly associate with each other. As Kae1 and Cgi121, or Pcc1 and Cgi121, do not directly bind to each other we did not see a reason for doing the experiments.

3. In Supplementary Table 1, the following changes are required:
a) The space group should be written as ‘P 3221’ instead of ‘P3221’.

We have corrected this error in the revised manuscript.

b) Units of B factors?

The units are $Å^2$. We have added the B factor units in the revised manuscript.

c) Also, describe Rfree

We have added the missing details for the calculation of Rfree in **Supplementary table 1**.

The table now reads:

**Refinement**		
Resolution (Å)	49-3.11	36-3.34
No. reflections (work/free)	10765/611	11611/593
R_{work}/ R_{free}	0.248/0.266	0.223/0.255

The legend of the table now reads:

' 5% of reflections were set aside for Rfree calculations. '

4. In line 120 the authors state "…compared to the structure of mjCgi121 without tRNA (RMSD =1.6 Å)", the number of Cα atoms used for the superpositioning is not mentioned.

The information of number of Cα atoms used for each superposition is now provided in the revised manuscript (page 5 line 128, page 5 line 143).

5. The authors have done extensive mutational studies.
The rationale for Cgi121 mutations such as M60E, F21K, G80W, I72K is not obvious and needs to be explained.

As requested, we have elaborated on the rationale for Cgi121 mutations in the revised manuscript as follows (page 6 lines 155-157).

' We next mutated a selection of tRNA contact surface residues on *mj*Cgi121 to alter either the charge or size properties (Phe21Lys, Lys56Ala/Glu, Met60Glu/Lys, Gln71Ala, Ile72Glu/Lys, and Gly80Trp) to examine effects on tRNA binding (**Fig. 3a**). '

However, throughout the article, the authors have not commented on the conservation of these residues, and no structure-based sequence alignments are provided.

We have added a new figure panel showing the sequence conservation of the Cgi121 residues selected for mutational analysis (**Supplementary Fig. 2e**). To compliment partial sequence alignments of the predicted tRNA contact residues of archaeal Bud32, Kae1 and Pcc1 with the

corresponding proteins in yeast (**Supplementary Fig. 7c**, **e** and **f**), we have now added a reference to detailed sequence alignments presented in a previous publication from our lab for these subunits (Mao et al., Mol Cell, 2008, see page 10 lines 297-298).

6. In line 265 the authors state "the N6 acceptor site of A37 lies only 7.3 Å from the corresponding acceptor site (hydroxyl group of C6'') of tobramycin". What is the distance from the donor atom? this needs to be sown in the figure as well.

The distances between the predicted position of the N6 acceptor site and the donor atom of TC-AMP is now shown in revised **Fig. 4d**.

7. In multiple instances (line 276, 277, 299, 301), the authors specify the exact atoms of tRNA interacting with the KEOPS complex. Since the mutational studies of the full complex is based on a model supported with negative stain, and in the absence of an atomic structure, the authors may avoid specifying such intricate interactions.

In the revised manuscript, we now make it clearer that the interactions between Bud32, Kae1 and Pcc1 with the tRNA are predicted based on a model. For example, added text on page 10 lines 287-289 now reads as follows (text added in the revised form of the manuscript are highlighted in bold):

> '**Based on modelling**, in *mj*Bud32, Arg60 in helix-αC **is predicted** to make attractive interactions with G68-G69 while Glu152 in the activation loop **is predicted** to make repulsive interactions with C10-U11 of the tRNA (**Fig. 5a**).'

8. In figure 7c, along with the ANN containing tRNAs the Cgi121-Bud32-Kae1 complex shows equal ATPase activity also with the non-ANN containing tRNAAla, which is perplexing and needs an explanation for this erroneous and non-specific binding and indicative of ANN is not the sole discriminator.

We agree with this reviewer that it is perplexing that tRNA Ala activates Bud32 ATPase activity even though it is not an ANN-decoding tRNA. In new experiments, we now explore the determinants of tRNA that appear to contribute to Bud32 activation.

We first tested the hypothesis that ATPase activation is not related directly to being a KEOPS substrate (new **Supplementary Fig. 8b**). We specifically show that in *mj*$tRNA^{Lys}$, mutation of the 36-UAA-38 motif (which is a hallmark of ANN-decoding tRNAs) did not disrupt ATPase activation by this tRNA. Thus, ATPase activation is not directly related to the ability to accept the t6A modification.

Next, we generated a detailed sequence alignment comparing ANN-decoding tRNAs and Ala tRNAs, members of which we experimentally showed to activate Bud32 ATPase activity, with non-ANN-decoding tRNAs that do not activate Bud32. Through these alignments, we have identified a new feature in *M. jannaschii* tRNAs, consisting of the dinucleotide pair CU at positions 10 and 11, that is strongly conserved in tRNAs predicted to activate Bud32 (new **Supplementary Fig. 8c**).

Interestingly, this motif is located on the predicted contact surface between the tRNA and the active site region of Bud32 in the KEOPS-tRNA holo-enzyme substrate model (new **Supplementary Fig. 8d**). Strikingly, mutation of the 10-CU-11 motif in Lys, Met, Thr, Asn and Ala tRNAs disrupted their ability to activate Bud32 when part of KEOPS. Mutation of this element also inhibited the t6A modification of tRNA substrates by KEOPS (new **Supplementary Fig. 8c-f**, page 15 lines 30-32 and page 16 lines 1-21). To verify that the mutant tRNAs are properly folded, we showed that Lys and Asn tRNA mutants maintained an ability to bind KEOPS using the 647-CC probe competitive displacement assay (**Supplementary Fig. 8g**). Thus, we have identified a new element present in a subset of *M. jannaschii* tRNAs that is required for the activation of Bud32 ATPase activity and for the t6A modification of ANN-decoding tRNAs.

Sequence alignments of the corresponding tRNAs in yeast shows that the aforementioned relationships may have additional complexity since only the C at position 10 is an invariant feature of ANN-decoding and Ala tRNAs. In addition, a sizable portion of non-ANN decoding tRNAs (72 out of 173) also harbor a C at positions 10. Thus, while C at position 10 may be required for the Bud32 activation potential of tRNAs it may not be sufficient in yeast. Further testing, which is beyond the scope of this study, is required to solidify our understanding of the mechanism of ATPase activation by tRNA.

9. Can the authors comment on what is the exact role of Pcc1?

To address this point, we have added the following text to the revised manuscript (Page 18 lines 547-551).

> ' Pcc1 is not required for tRNA binding by KEOPS or for the activation of Bud32 by tRNA, but it is required for tRNA modification by KEOPS (**Supplementary Fig. 1b**). While the precise role of this subunit also remains an open question, we speculate that based on the proximity to both Kae1 and tRNA, Pcc1 and perhaps the Bud32 tail may help guide A37 to the active site of Kae1. '

10. Throughout the article, the authors have subdivided the section into small paragraphs. The authors may consider avoiding too small (3 lines) paragraphs by merging with the earlier or later.

As suggested, we have merged seven short paragraphs with the following or previous paragraph (see page 5 line 122, page 6 line 148 and line 163, page 7 line 180, page 10 line 298, page 13 line 391, page 15 line 446).

11. As per the current model, the KEOPS recognizes the CCA but not ΔCCA and 2X-CCA, which is essential to modify only the correctly folded tRNAs. However, the mitochondrial (Qri7) and bacterial (TsaB, D, E) systems modify tRNAs irrespective of the CCA terminal which can be erroneous. Can the authors explain the physiological relevance of such a mechanism in bacteria and mitochondria?

We agree that this is an interesting question. One possible explanation might be that in eukaryotes translation within the cytoplasm is more complex and subject to more regulation than translation in bacteria or in the mitochondria. Another possible explanation is that in bacteria, some tRNA genes are transcribed directly with a CCA (for example, see Ardell and Hou, BMC Genomics, 2016) and therefore the presence of a CCA tail is not a distinct hallmark of a mature tRNA.

Due to the speculative nature of these points we have not included this commentary in the revised manuscript. Instead we simply raise this very thought-provoking question in the discussion (page 20, lines 589-590).

12. In extended data Fig 3C, the authors need to provide SEC profiles for apo protein and free tRNA for comparison.

SEC profiles of apo-tRNA and apo-Cgi121 are now shown on revised **Supplementary Fig. 3c**.

Reviewer #4 (Remarks to the Author):

Author Comments:

Overall, the authors have conducted an excellent and thorough analysis, and the methods employed and the means of reporting the data are all adherent to community HDX guidelines. Furthermore, the authors are applauded for making their data available on PRIDE.

We thank the reviewer for their kind statements.

A couple of queries:
1) From the Woods Plot in figure SF6D, it appears that there is little peptide coverage of the first 50 amino-acids of Kae1. Do the authors believe this could impact on their interpretation of the data?

The missing sequence in Kae1 is between the two extremes of HDX "footprints". While having coverage of the missing area would be useful, it would not affect our conclusion of a binding-induced effect spanning the complex.

2) Also from SF6D, there are 2 peptides at the very N-terminus of Bud32 which see significant enhancements of HDX (>-0.2 delta value). This seems at odds with the other peptides within this region. The authors have not shown these as significant changes (as they are not coloured blue (as is my understanding). Why were these peptides excluded from the results? Given the magnitude of the difference, I would expect these to be significant - Why were these peptides excluded from the results?

As noted in the methods, according to ref[63] (Bennett et al., 2010, Chem Biol.), two criteria requirements apply for significance. First, a peptide must have a delta value in excess of two StDev based on mass shift noise (measured from the non-significant subset of data – set by the dashed horizontal lines in **Supplementary Fig. 6d**). Second, a peptide must also have a p value of less than 0.05 in a test of the null hypothesis, within the set of replicates (bound vs free). So in these two cases, the average shifts were large enough (first criteria) but the variance too high to be significant (second criteria). This is likely to have been caused by spectral overlap issues.

Minor:
1) Supplementary Table S8: Table headers are not visible (charge, Sequence Start).

Table headers are now visible in the updated version of **Supplementary Table 9**. We thank the reviewer for pointing this out.

2) Supplementary Table S8: Units of RT are not shown

The units of RT are minutes. We have revised **Supplementary Table 9** accordingly. We thank the reviewer for noticing that the information was missing.

Glenn Masson

Reviewer #1 (Remarks to the Author):

The authors have addressed my concerns and comments well. I am satisfied with the current state of the manuscript.

Reviewer #3 (Remarks to the Author):

The authors have addressed all my concerns and suggestions satisfactorily.
